# Predicting rifampicin resistance mutations in bacterial RNA polymerase subunit beta based on majority consensus

**DOI:** 10.1186/s12859-021-04137-0

**Published:** 2021-04-22

**Authors:** Qing Ning, Dali Wang, Fei Cheng, Yuheng Zhong, Qi Ding, Jing You

**Affiliations:** Guangdong Key Laboratory of Environmental Pollution and Health, School of Environment, Jinan University, Guangzhou, 511443 China

**Keywords:** Resistance mutation, Machine learning, Classifier, Rifampicin, Prediction

## Abstract

**Background:**

Mutations in an enzyme target are one of the most common mechanisms whereby antibiotic resistance arises. Identification of the resistance mutations in bacteria is essential for understanding the structural basis of antibiotic resistance and design of new drugs. However, the traditionally used experimental approaches to identify resistance mutations were usually labor-intensive and costly.

**Results:**

We present a machine learning (ML)-based classifier for predicting rifampicin (Rif) resistance mutations in bacterial RNA Polymerase subunit β (RpoB). A total of 186 mutations were gathered from the literature for developing the classifier, using 80% of the data as the training set and the rest as the test set. The features of the mutated RpoB and their binding energies with Rif were calculated through computational methods, and used as the mutation attributes for modeling. Classifiers based on five ML algorithms, i.e. decision tree, k nearest neighbors, naïve Bayes, probabilistic neural network and support vector machine, were first built, and a majority consensus (MC) approach was then used to obtain a new classifier based on the classifications of the five individual ML algorithms. The MC classifier comprehensively improved the predictive performance, with accuracy, F-measure and AUC of 0.78, 0.83 and 0.81for training set whilst 0.84, 0.87 and 0.83 for test set, respectively.

**Conclusion:**

The MC classifier provides an alternative methodology for rapid identification of resistance mutations in bacteria, which may help with early detection of antibiotic resistance and new drug discovery.

**Supplementary Information:**

The online version contains supplementary material available at 10.1186/s12859-021-04137-0.

## Background

Antibiotic resistance has become one of the greatest threats to public health all over the world. Pathogens with antibiotic resistance add difficulty to deal with infections and lead to increasing mortality. As stated by the United Nations in 2019 [1], at least 700 thousands of deaths are caused by infections of resistant pathogens every year, and this number will soar to 10 million annually by 2050 if no action is taken. Among the ever-growing resistant pathogens, *Mycobacterium tuberculosis* (MTB) is of particular concern because this species is a causative agent of tuberculosis, a highly-ranked death cause worldwide nowadays [1]. Rifampicin (Rif), an antibiotic of rifamycin class, has been extensively used to treat tuberculosis. However, there has been an increasing occurrence of Rif resistance in MTB, raising emerging health concerns [2, 3]. It was estimated that approximately 484,000 new cases of Rif-resistant tuberculosis and 214,000 Rif-resistant tuberculosis related deaths occurred in 2018 [4].

Antibiotic resistance in bacteria can originate from multiple sources, such as acquiring antibiotic resistance genes (ARGs) carried by mobile genetic elements (e.g. plasmids and transposons) [5], overexpression of multidrug efflux [6] and de novo resistance mutations in bacterial genomes [7]. For Rif, resistance is primarily caused by single point mutations in RNA polymerase (RNAP), an enzyme that is essential for RNA synthesis [8, 9]. Rif typically binds to the β subunit of RNAP (RpoB) and blocks RNA synthesis, leading to the death of bacterial cells. Mutations in RpoB might cause changes of RpoB conformation and prevent Rif from binding to RpoB, resulting in loss of bactericidal activity of Rif. It should be noted that mutations occur randomly at any site of RpoB and do not always cause detrimental outcomes, instead, only those inducing resistance phenotypes (known as resistance mutations) are undesired and are more noteworthy. Currently, resistance mutations in bacteria are mostly identified through experimental approach, for example, to extract and sequence the DNA segments in the mutants, which are time- and labor-consuming. Since there are lots of probabilities for mutations in a given protein, it is of great significance to develop predictive, other than experimental, approaches for quick screening of the resistance mutations.

Machine learning (ML) is a branch of artificial intelligence (AI), which learns from massive amounts of data and reveals patterns and features in the data for predictions and decision making based on new data. Nowadays, ML algorithms have found applications in a variety of fields, such as speech recognition, traffic prediction and recommender systems [10–13]. In particular, ML algorithms have been increasingly used for solving classification problems in molecular biology and toxicology. For example, Murakami and Mizuguchi [14] developed a naïve Bayes (NB) classifier for predicting the protein–protein interaction sites and Zhang et al. [15, 16] constructed NB classifiers for predicting drug-induced liver injury and mitochondrial toxicity in human. Classification is an important issue to understand the question whether mutations occurring in bacterial RpoB could lead to Rif resistance, therefore, ML algorithms would be a useful tool for predicting the outcomes of these mutations, yet there have been rare such attempts in the literature.

In this paper, we reported a novel ML-based method for predicting the Rif resistance mutations in bacterial RpoB. Mutations that have been validated experimentally to confer (positive) or not to confer (negative) Rif resistance were collected from the literature and Genome-wide Mycobacterium tuberculosis Variation (GMTV) database. Five ML algorithms, i.e. decision tree (DT), k nearest neighbors (kNN), NB, probabilistic neural network (PNN) and support vector machine (SVM) were employed for modeling using the collected data with both internal and external validations. A majority consensus (MC) classifier was finally obtained based on the classification results of five individual ML algorithms, which showed improved predictive performance. The ML-based classifier provides an alternative approach for quickly identifying Rif resistance mutations in bacteria.

## Results

### Negative and positive mutations in RpoB of MTB

**Fig. 1:**
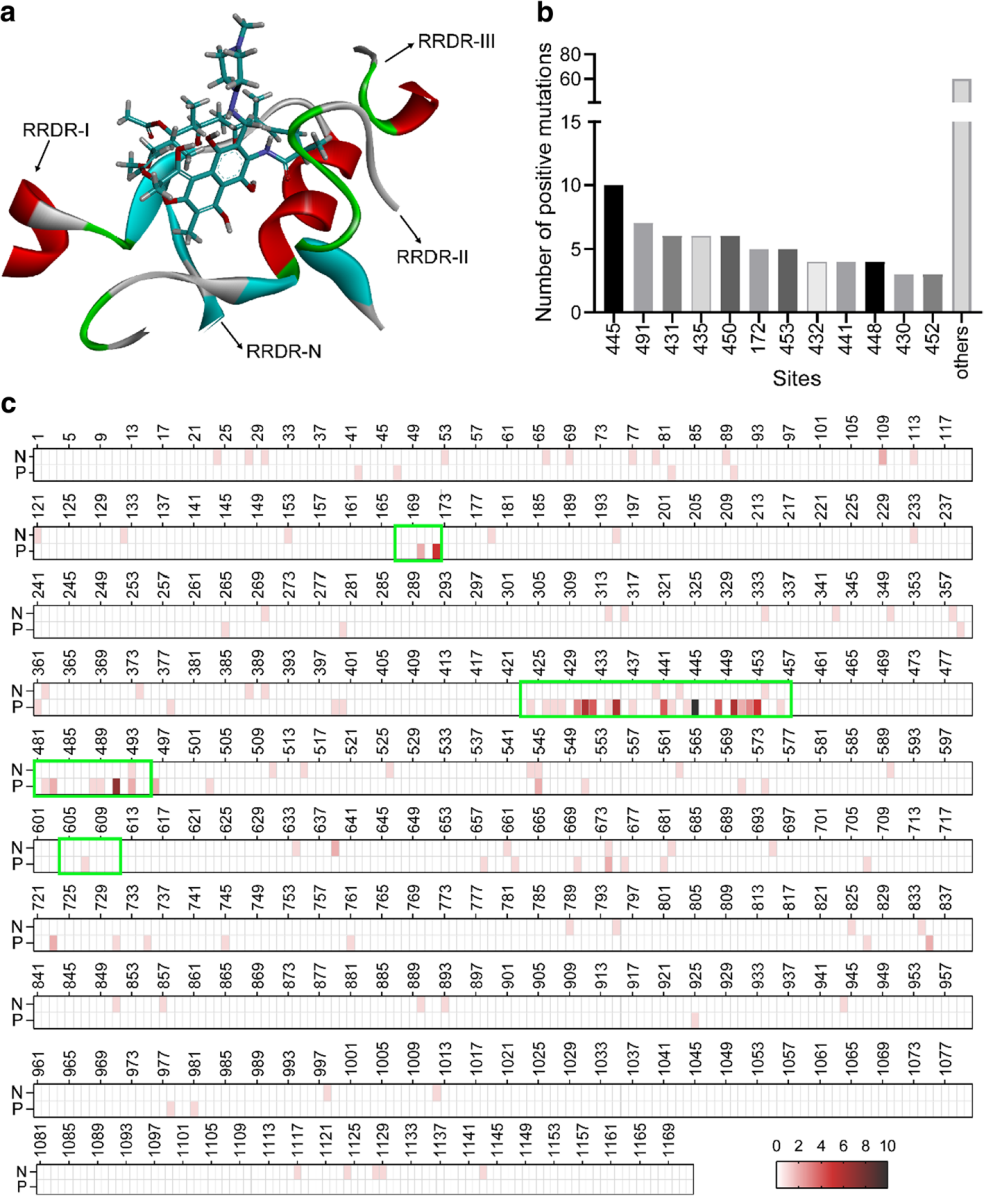
**a** A side view of the structure of the rifampin (Rif) binding pocket formed by the Rif resistance-determining regions (RRDRs) of RpoB from MTB (PDB ID: 5UHC). **b** Number of positive mutations obtained at different sites of RpoB. **c** Distribution of the negative (N) and positive (P) mutations in the protein sequence of RpoB from MTB. The color gradients represent the different number of mutations at the indicated sites. The regions in the green frames are the RRDR-N, RRDR-I, RRDR-II and RRDR-III, respectively

Mutations occur both spontaneously and under stresses during DNA replication and repair processes. Herein, mutations that confer bacterial resistance against antibiotics are referred to as positive mutations, while those do not induce any changes in bacterial resistance phenotype are assigned as negative mutations. Resistance to Rif in bacteria is thought to be solely attributed to single point mutations of *rpoB* gene that encodes RpoB, the target of the Rif molecules [17]. Rif resistance mutations primarily occur within Rif resistance-determining regions (RRDRs, Additional file 1: Table S1) of RpoB that are involved in the formation of the Rif-binding pocket (Fig. 1a) [18]. In the literature, the positive mutations were usually determined by sequencing the *rpoB* gene of the isolated strains and comparing it with the wild type sequence. In the present research, a total of 186 RpoB mutations (Table 1) were obtained from the literature [18–22] and GMTV database. Specifically, 123 positive mutations were collected at 63 amino acid positions, among which H445 had the maximum number (10) of mutations, followed by I491, S431, D435 and S450 (Fig. 1b). A majority (78 of 123) of the positive mutations were within the RRDRs, especially in RRDR-I (Fig. 1c), suggesting this region was the hotspot where resistance mutations occurred the most frequently. As for the negative mutations, 63 amino acid changes were gathered from 61 sites, which were more decentralized in the protein sequence of RpoB (Fig. 1c). In particular, only 4 negative mutations were found within RRDRs (3 in RRDR-I and 1 in RRDR-II) (Fig. 1c), which were much fewer than the positive mutations. This supported the fact that mutations occurring in RRDRs were more likely to become resistant.

**Table 1:** Negative and positive mutations in RpoB of *M. tuberculosis* (MTB)

Type	Amino acid change
Positive (123)	L42F L47R E82G I90L V170F V170G Q172H Q172K Q172L Q172P Q172R D265G P280S V359A T361I L378R T399A T400I F424L G426D T427P S428R L430P L430Q L430R S431A S431F S431P S431R S431T S431Y Q432K Q432L Q432P Q432R M434I D435A D435F D435G D435N D435V D435Y N437D S441F S441L S441Q S441Y G442V T444R H445C H445D H445F H445G H445L H445N H445P H445Q H445R H445Y R448C R448H R448L R448S S450F S450L S450P S450Q S450W S450Y A451E A451V L452H L452P L452R G453A G453C G453D G453S G453V G456S T482P P483L P483R I488V G489C I491F I491L I491M I491N I491S I491T I491V S493F S493Y V496L V496M F503S D545E D545N P551S D571A D574E R607H N658D R662H A670D H674R H674Y T676P C681W M707T H723D H723Y L731P L735Q H745Y E761D R827C H835P H835R I925V E978D G981D
Negative (63)	N24D G28R P30S D53N E66K A69P V77M L80V P89L V109I D109E V113I M121I E132D M153T V179A S195R P233Q D270E L314V L316V A334D H343Q T350I P358L D362H G374S S388L M390T M440V L443F P454L N493S R511L D515Y T526S A544V D545A E563D A590G D634G E639G E639Q R661Q H674Q P682T V695L I789V E795V E825G P834L D851G A857T G890D L893R K944E A998V D1012G V1117L S1124A L1128Q V1129A A1143T

### Evaluation of the mutated structures

**Fig. 2:**
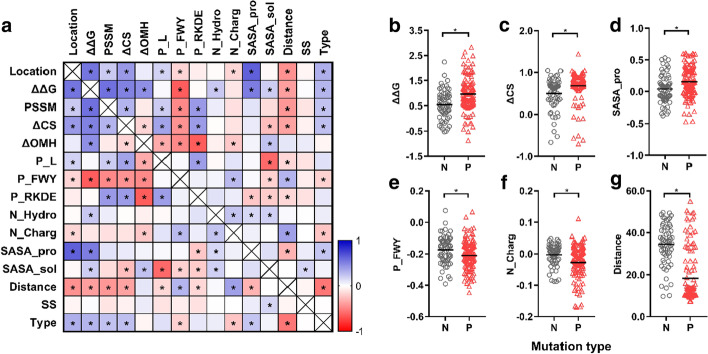
**a** Correlation matrix for the features of the mutated RpoB. The correlation of each pair of the features was quantified by a Pearson’s correlation coefficient, which ranges from  − 1 to 1. The asterisk indicates a significant difference (*p* < 0.05). **b**–**g** Distributions of ΔΔG (**b**), ΔCS (**c**), SASA_pro (**d**), P_FWY (**e**), N_Charg (**f**) and distance values (**g**) for the two mutation phenotypes (N stands for negative and P for positive). Significant differences (*p* < 0.05) are indicated by asterisks (*). The straight lines in **b**–**g** indicate the medians

Twelve features (Additional file 1: Table S2) were obtained by PremPS Server for each mutated RpoB using 5UHC as a template. Besides, the distance between each amino acid and the active site of 5UHC was calculated, which, together with the secondary structure (denoted as SS) of the amino acid, were also employed as the attributes of the mutations. Pearson’s correlation coefficient of each pair of the computational features was shown in Fig. 2a. Among these features, ΔΔG is usually used for predicting the stability of the protein caused by mutations [23]. It is obtained by quantifying the change of unfolding Gibbs free energy (ΔG) of a protein after a single point mutation. According to the results, a majority (166) of the 186 mutated RpoB had greater ΔGs than the wild type RpoB (i.e., ΔΔG > 0), suggesting destabilizing effects of these point mutations (Additional file 1: Table S3). Moreover, the phenotypes (positive or negative) of the mutations were found significantly correlated (*p* < 0.05) to ΔΔG. As shown in Fig. 2b, ΔΔG values of the negative mutations fell into the range from − 0.53 to 2.24 kcal mol^−1^, with a median of 0.50 kcal mol^−1^, whereas ΔΔG values of the positive mutations distributed in a larger range (− 0.87 to 2.2 kcal mol^−1^) and with a greater median (0.92 kcal mol^−1^). Statistical analysis with an unpaired t test (Mann Whitney test) suggested that the difference of the ΔΔG values between negative and positive mutations were statistically significant (*p* < 0.05) (Fig. 2b). This result implied that a mutant with a higher ΔΔG value had a greater inclination to become resistant. The same cases were found with ΔCS (the changes of conservation after mutation) and SASA_pro (the solvent accessible surface area of the mutated residue in the protein), which were both significantly correlated with the mutation phenotype and presented greater values in positive mutations (Fig. 2c, d). For P_FWY (the fraction of aromatic residues F, W or Y buried in the protein core) and N_Charg (the number of changed amino acids R, K, D or E in the protein), however, they were correlated with the mutation phenotype in an opposite pattern to ΔΔG, ΔCS and SASA_pro, i.e. the negative mutations tended to have greater P_FWY and N_Charg values (Fig. 2e, f). With respect to the distance, it was found that the negative mutations had greater distances than the positive ones (Fig. 2g), which suggested that the positive mutations were more likely to occur at amino acids that are closer to the active site.

### Interactions between mutated RpoB and Rif molecule

**Fig. 3:**
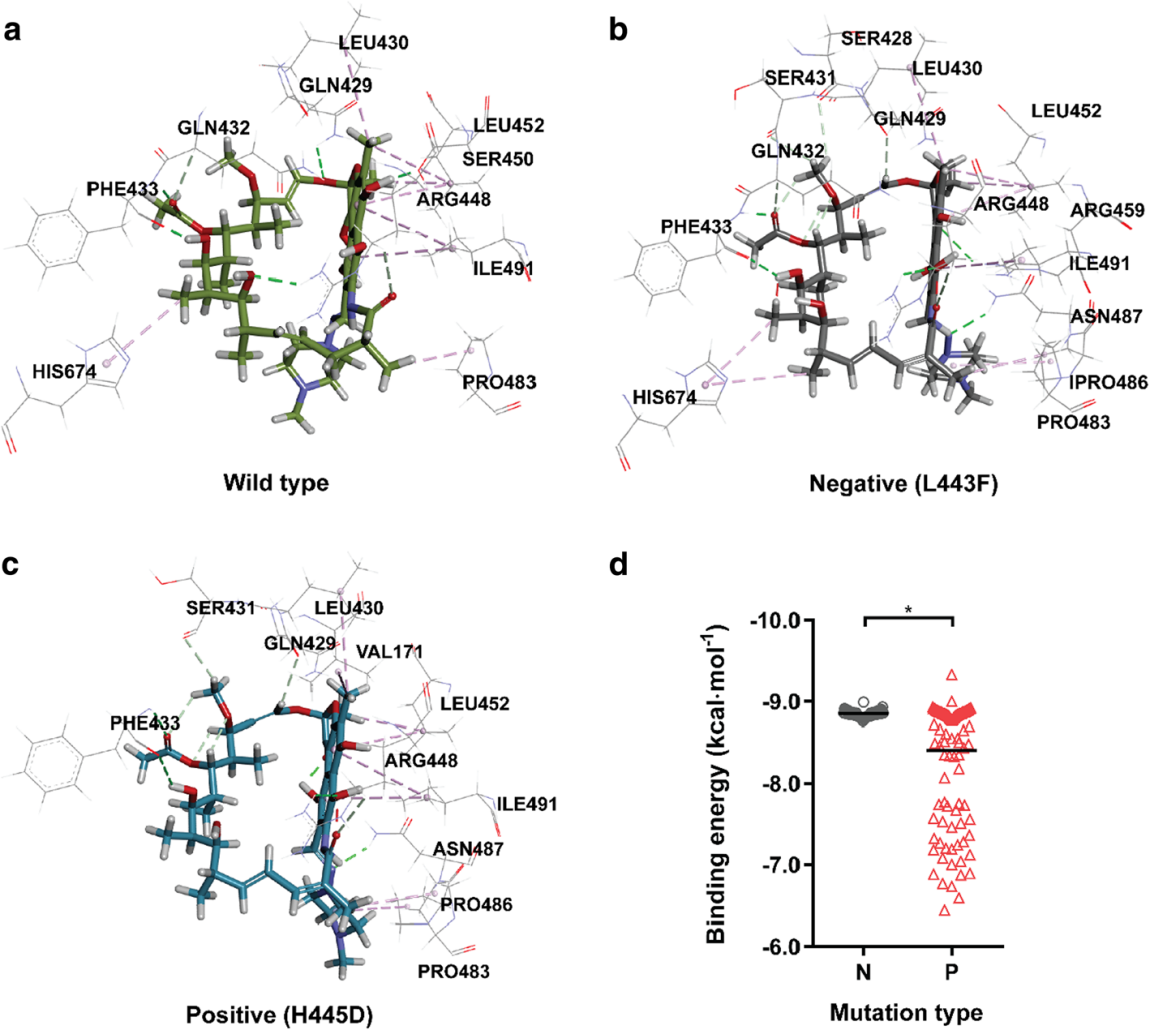
Interactions between the rifampin molecule and wild type (**a**), negative (**b**) and positive mutated RpoB (**c**). The wild type RpoB-Rif complex were obtained from the Protein Database (5UHC), while L443F and H445D were chosen as representatives of negative and positive mutations, respectively. **d** Distributions of the binding energies of Rif with RpoB mutants. N stands for negative and P for positive

The combination of Rif molecule with the mutated RpoB (both positive and negative) was simulated by molecular docking. According to the results, the docked Rif molecules in the negative and positive mutants presented similar poses with only slight differences in the orientation of the 1-methylpiperazine tail. The docked poses also resembled the Rif pose in the wild type RpoB (5UHC). As shown in Additional file 1: Fig. S1, the macrocyclic moieties of Rif in wildtype RpoB, L443F and H445D (representatives of negative and positive mutations, respectively) presented a good overlap, while the 1-methylpiperazine tails of Rif in the three proteins had different orientations. Then, the interactions between RpoB and Rif molecules were analyzed and compared. In the wild type RpoB, most of the residues interacting with the Rif molecule were within RRDRs (Fig. 3a). Specifically, ARG448, GLN429, GLN432, PHE433 and SER450 were involved in hydrogen bonds with Rif, while ILE491, LEU430, LEU452 and PRO483 played important roles in hydrophobic interactions with Rif. Apart from the residues within RRDRs, HIS674 was found to form hydrogen bond with Rif in the wild type RpoB. In the cases of negative (L443F) and positive mutations (H445D) (Fig. 3b, c), most of the interactions found in wildtype RpoB were also observed, but the hydrogen bond between HIS674 and Rif disappeared in the positive mutant H445D. The interactions between RpoB and Rif molecules were quantitatively characterized by the binding energies (Additional file 1: Table S3) obtained by docking. As shown in Fig. 3d, the binding energies for negative RpoB mutants were highly centralized and close to that of the wild type RpoB (− 8.88 kcal mol^−1^) while for the positive mutants distributed in a wider range (from − 9.33 to − 6.44 kcal mol^−1^). This suggested that the positive mutants tended to present greater changes in the binding energies with Rif than the negative mutants.

### Classifiers using different machine learning algorithms

**Fig. 4:**
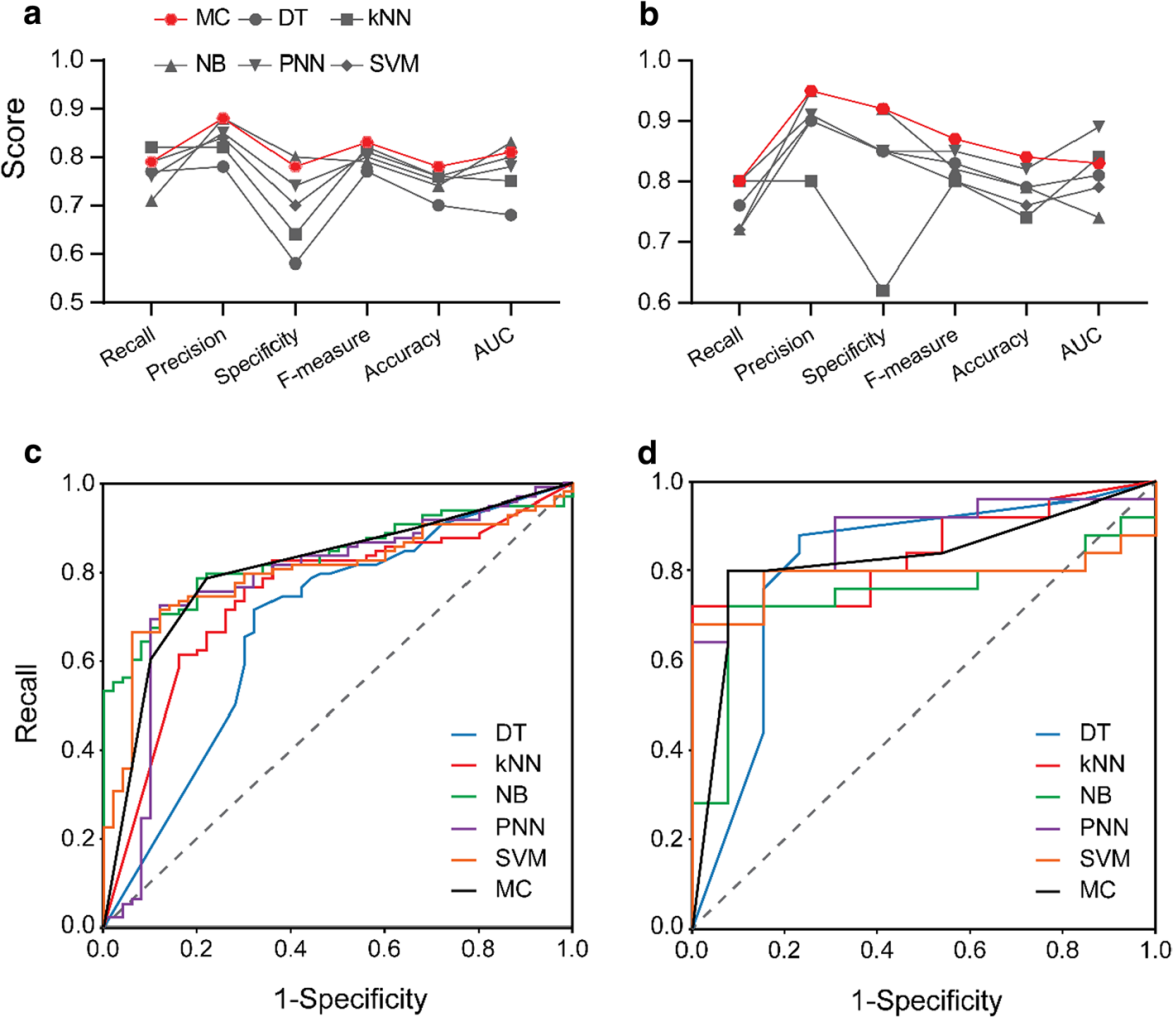
Comparison of the evaluation parameters of different classifiers for the training (**a**) and test sets (**b**) and receiver operating characteristic curves (ROCs) of the classifiers for the training (**c**) and test sets (**d**). DT: decision tree; kNN: k nearest neighbors; NB: naïve Bayes; PNN: probabilistic neural network; SVM: support vector machine. AUC: area under the curve

The mutation database (186 data) was split randomly with a stratified sampling method into a training set (80%) and a test set (20%). Five ML classifiers were developed with DT, kNN, NB, PNN and SVM algorithms using the calculated features as the attributes of the mutations, and ten-fold cross validation was performed for the internal validation of the classifiers. The confusion matrixes of the classifiers for both training and test sets were provided in Additional file 1: Table S4, and the classification details of each mutation by the classifiers (including the class assignment and the class probabilities) were provided in Additional file 1: Table S5. The evaluation parameters of the classifiers were shown in Table 2, and the comparison of them were depicted in Fig. 4.

**Table 2:** The performance of the classifiers on the training and test dataset

	Training set						Test set					
	DT	kNN	NB	PNN	SVM	MC	DT	kNN	NB	PNN	SVM	MC
Recall	0.77	0.82	0.71	0.76	0.79	0.79	0.76	0.80	0.72	0.80	0.72	0.80
Precision	0.78	0.82	0.88	0.85	0.84	0.88	0.90	0.80	0.95	0.91	0.90	0.95
Specificity	0.58	0.64	0.80	0.74	0.70	0.78	0.85	0.62	0.92	0.85	0.85	0.92
F-measure	0.77	0.82	0.79	0.80	0.81	0.83	0.83	0.80	0.82	0.85	0.80	0.87
Accuracy	0.70	0.76	0.74	0.75	0.76	0.78	0.79	0.74	0.79	0.82	0.76	0.84
AUC	0.68	0.75	0.83	0.78	0.80	0.81	0.81	0.84	0.74	0.89	0.79	0.83

*DT* decision tree, *kNN* k nearest neighbors, *NB* naïve Bayes, *PNN* probabilistic neural network, *SVM* support vector machine, *MC* majority consensus, *AUC* area under the curve

For the training set, kNN and SVM classifiers had the greatest accuracy with a same score of 0.76, followed by PNN (0.75), NB (0.74) and DT (0.70), while for the test set, the rank of the accuracy for the five classifiers was PNN (0.82) > NB (0.79) = DT (0.79) > SVM (0.76) > kNN (0.74). The precisions of the five classifiers, which represent the proportion of the correctly predicted positives, ranked as NB (0.88) > PNN (0.85) > SVM (0.84) > kNN (0.82) > DT (0.78) for the training set, and NB (0.95) > PNN (0.91) > SVM (0.90) = DT (0.90) > kNN (0.80) for the test set, respectively. The recall (also known as sensitivity), which represents the true positive rate, ranked in a similar order for the training and test sets, with a highest score for kNN and a lowest score for NB, whereas the specificity that represents the true negative rate ranked in an opposite order to the recall in both training and test sets.

It should be noted that, the predictive models developed herein are intended to identify the potential resistance mutations (i.e., positive mutations), thus it is important that the positive mutations are predicted correctly. Therefore, the recall is an important estimator of the than the classifiers. However, a greater specificity (true negative rate) of the classifiers may have more clinical relevance, because the irrational use of ineffective drugs to a resistant strain should be avoided in case of a poor outcome and further evolution and transmission of a resistant pathogen. In view of this, a high specificity is highly desirable for such a classifier. In the present study, kNN had the greatest recall (0.82) but a poor specificity score (0.64) for the training set, while PNN on the test set presented both a high recall (0.80) and a high specificity (0.85). F-measure, which conveys the balance between recall and precision, was also calculated. As listed in Table 2, kNN and PNN had the highest F-measure scores for training (0.82) and test set (0.85), respectively. Furthermore, the receiver operating characteristic (ROC) curves of the classifiers (Fig. 4), which demonstrated the connection between recall and specificity, were obtained by plotting the recall versus “1-specificity” across all possible thresholds for the test. A ROC curve that is closer to the left-hand and top borders of the ROC space points to a high accuracy of the classifier, and the area under the ROC curve (AUC) was used to represent the measure of separability of the classifiers. According to the ROCs (Fig. 4) and AUC scores (Table 2), the five classifiers exhibited varying separability capacities, with NB (0.83) and PNN (0.89) showing the greatest AUC scores for the training and test sets, respectively.

### Majority consensus classifier

The MC classifier was developed based on the classification results of the five individual ML algorithms, which assigned “positive” to a mutation that had been classified as “positive” by more than two of the ML algorithms. The prediction results for the MC classifier on the training and test data were shown in Table 2. In general, the MC classifier showed a better predictive performance than the five individual ML algorithms, with evaluation parameters being higher in most of the cases (Fig. 4a, b). For example, the accuracy of the MC classifier increased to 0.78 and 0.84 for the training and test sets, respectively. That is, 116 of 148 mutations in the training set and 32 of 38 mutations in the test set were correctly predicted (Additional file 1: Table S4). The same case was found with F-measure, which had higher scores for MC classifier on both training (0.83) and test sets (0.87) than the five individual ML algorithms. It should be noted that there were a few cases that the evaluation metrics of the MC classifiers were equal to or even lower than the ML algorithms. For example, the recall of the kNN classifier on the training set (0.82) was greater than the MC classifier (0.79), but the specificity of kNN was only 0.64, which was much lower than the MC classifier (0.78). Therefore, the MC classifier had a better balance of all the evaluation parameters than the individual algorithms. In particular, the MC classifier had high specificity scores, especially for the test set (0.92). As mentioned above, a higher specificity of such a classifier may have more clinical relevance, thus the MC classifier may have a greater application potential.

### Application of the classifiers

**Fig. 5:**
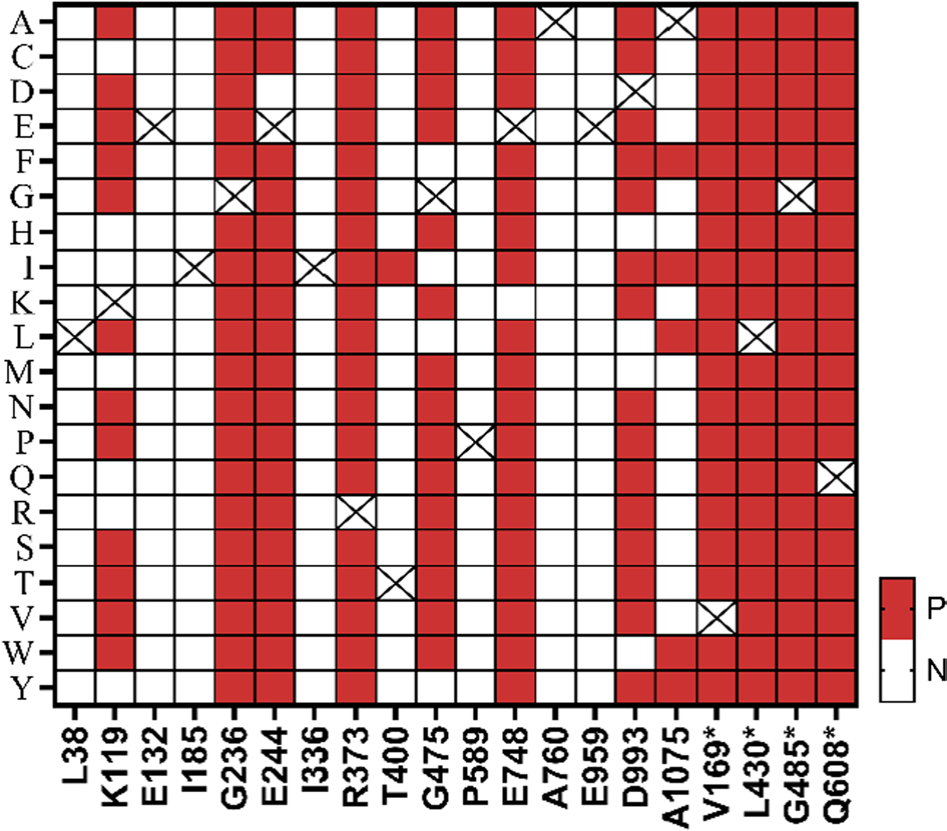
Classifications of the resistance types of RpoB by majority consensus classifier. N stands for negative and P for positive. Mutations in 20 random positions in RpoB of MTB were chosen for the prediction. X axis labels represent the location and the single-letter abbreviation of the amino acid residues, y axis labels are the abbreviation of the mutated residues. An asterisk (*) in the x axis indicates that the position is within the rifampin resistance determining regions (RRDRs)

To verify the performance of the classifiers, we used them to predict the phenotypes of mutations in 20 random positions in RpoB, including 4 in RRDRs and 16 in non-RRDRs. A total of 380 mutants (20 × 19, 20 amino acid sites and 19 possibilities for each site) were built through PremPS server and were investigated for their interactions with Rif molecule by LeDock. The PremPS-based features, binding energies, distance and SS were gathered for these mutations as attributes for prediction (Additional file 1: Table S6). The detailed prediction results of these mutations were provided in Additional file 1: Table S7, and a heatmap displaying the MC classifications were shown in Fig. 5. According to the MC classifications, 198 of 380 mutations were predicted as positive and 182 as negative. In particular, all of the mutations within the RRDRs were predicted as positive, while only 40% (122 of 304) of the mutations in non-RRDRs were classified as positive. The results were supportive again of the fact that mutations in RRDRs are more likely to confer Rif resistance. However, it should be noted that the predicted resistance mutations shown in Fig. 5 do not necessarily occur in real conditions, they only represent that the mutations have a high probability to confer resistance if they occur. Further experiment should be carried out for validation of these predictions.

Furthermore, the performance of the classifiers in predicting resistance mutations of MTB against another anti-tuberculosis drug isoniazid (INH) was verified using 89 mutation data of catalase-peroxidase (KatG) in MTB. KatG is responsible for converting INH to its active form in MTB, and mutations of KatG have been considered as a major cause of INH resistance. In the present study, we collected 89 mutation data of KatG from the literature [21, 24], including 40 positive and 49 negative mutations (Table S8). The classification results of the five ML algorithms and the MC classifiers were provided in Table S9, and the evaluation metrics of these classifiers were shown in Table 3. According to the results, the accuracies of the five ML algorithms ranged from 0.60 to 0.76 and F-measures ranged from 0.66 to 0.77. The MC classifier showed higher scores of accuracy (0.75) and F-measure (0.76) than the five individual algorithms in most of the cases. In particular, the recall of the MC classifier reached up to 0.93, which suggested that this classifier had a great capacity of correctly identifying the INH resistance mutations in KatG. However, the specificity of the MC classifier was only 0.61, showing a poor capacity of predicting the susceptible mutations in KatG. This was likely because the classifiers in the present study were trained based on Rif resistance data only and may need improvement before scaling to other drugs.

**Table 3:** The performance of the classifiers in predicting mutations of KatG against isoniazid

	DT	kNN	NB	PNN	SVM	MC
Precision	0.61	0.53	0.72	0.55	0.69	0.66
Recall	0.85	0.88	0.70	0.83	0.88	0.93
Specificity	0.55	0.37	0.78	0.45	0.67	0.61
Accuracy	0.69	0.60	0.72	0.62	0.76	0.75
F-measure	0.71	0.66	0.71	0.66	0.77	0.77

*DT* decision tree, *kNN* k nearest neighbors, *NB* naïve Bayes, *PNN* probabilistic neural network, *SVM* support vector machine, *MC* majority consensus, *AUC* area under the curve

## Discussion

Antibiotic resistance is a major threat to public health and is of considerable concern. One of the mechanisms for antibiotic resistance is the mutations occurring in an enzyme target, causing structural modifications of the active sites that no longer allow the combination of the antibiotics [25]. Resistance originating in this way has been observed for many antibiotics. For example, mutations of *pbp*, a gene encoding penicillin-binding proteins, conferred bacteria with β-lactams resistance [26], and mutations of *rrnA* and *rrnB,* genes that encode 16S rRNA, conferred tetracycline resistance [27]. In the case of Rif, resistance usually arises as a result of single point mutations in bacterial RNAP, an enzyme that accommodates Rif molecule at the β subunit. Identification of the resistance mutations is essential for understanding the structural basis of the antibiotic resistance, and provides useful information for prevention of the resistance emergence and new drug discovery. In previous studies, the identification of the resistance mutations was usually achieved through labor-intensive and costly experiment, for example to isolate the resistance mutants and sequence the relevant gene segments followed by aligning the sequences with those of the sensitive strains [28, 29]. The experimental approach is thus incapable of rapid identification, and moreover, the identification with experimental approach usually lags behind the emergence of the resistance. Therefore, prediction of the resistance mutations based on known information is highly desired.

In previous studies, there have been some attempts that focused on the prediction of drug resistance mutations in MTB. For example, Walker et al. [21] reported a predictive approach for characterizing the drug resistance and susceptibility of mutations in MTB using whole-genome sequencing data. The authors gathered 120 resistance determining mutations and 772 benign mutations from 2099 MTB genomes, and used them as criterions to characterize the phenotypes of an independent validation set of 1552 MTB isolates. This approach predicted 89.2% of the validation set with a high sensitivity of 92.3% and a high specificity of 98.4%. For Rif resistance, the sensitivity and specificity were 96.8% and 99.2%, respectively. In addition, Miotto et al. [22] developed a standardized approach for classifying the phenotype of mutations in MTB through grading the confidence of the association between mutations and resistance. This approach performed well in identifying the Rif resistance mutations with a sensitivity of 89.6–90.3% and a specificity of 95.7–96.8% (95% confidence interval). Although these approaches had better predictive performance than the classifiers in the present work, their predictions relied heavily on the previously documented genes and mutations that had been validated experimentally to be resistance determining. So the sensitivity of these approaches might be limited if some of the resistance associated genes and mutations were absent from the training set (this is very likely to happen because our knowledge about the resistance genes and mutations is growing). On the contrary, the classifiers in the present study were developed based on the structure-associated features of the protein, like ΔΔG and the binding affinity, rather than simply on the genetic information. Therefore, the classifiers developed in this paper had a more solid mechanistic basis and a greater application potential. Likewise, a computational approach for predicting resistance mutations in dihydrofolate reductase was reported by Frey et al. [30], where a protein design algorithm was employed for generating positive and negative mutations, and the prediction was based on the analysis of the affinity between the mutants and the inhibitor. Comparing to this approach, the classifiers developed in the present paper takes into account not only the affinity but also a series of features of the mutants. Moreover, the predictive algorithms in the present work were trained using a collection of both negative and positive mutations, and is thus more reliable and explicable.

Recently, Jamal et al. [31] reported a work that used AI and ML algorithms to predict resistant and susceptible mutations in MTB. However, the method for developing predictive models in Jamal et al. [31] was debatable. When preparing the database for modeling, the authors labeled mutations with positive ΔΔG values as susceptible mutations, otherwise as resistant mutations. The problem was that ΔΔG was an indicator of the stability of a mutated protein, and it was improper to use this parameter alone for classifying the mutations, although this parameter might have some relationship with the mutation type. Moreover, in the following steps, the authors used this parameter again as an attribute of the mutations for training the classification models, which was also improper. Unlike this work, the data used for developing the predictive models in the present research were collected from the literature and the database, which had been validated experimentally. Moreover, we developed a MC algorithm based on the predictions of five individual ML algorithms, which improved the overall predictive performance. However, the classifier developed in the present work still has some limitations, for example, the classifications need further validation with experiments. Nonetheless, the present work provides an inspiration and an alternative methodology for rapid identification of resistance mutations in bacteria, which may be helpful for early detection of resistance and new drug discovery.

## Conclusion

A MC classifier was developed for predicting Rif resistance mutations in bacterial RNAP based on five ML algorithms (i.e. DT, kNN, NB, PNN and SVM). The features of the mutated RpoB and their combination with Rif molecule were studied by computational approaches and used for developing the predictive models. Estimates of the predictive models showed that the five individual algorithms had varying predictive performance with accuracy of 0.70–0.76 and 0.74–0.82 for the training and testing sets, respectively, while the MC classifier presented better predictions with a higher accuracy of 0.78 and 0.84 for training and test data, respectively. The performance of the classifiers was then verified using a set of data of INH resistance mutations in KatG. The predictions showed high accuracy, F-measure and recall scores, but with poor precision and specificity. Since the classifiers developed in the present study were trained on Rif resistance data only, they might need improvement before scaling to other drugs.

## Methods

### Mutation dataset preparation

Mutations that confer bacteria with Rif resistance are assigned as positive, otherwise as negative. Mutations in RpoB of MTB were collected from the literature [18–22] and GMTV database (http://mtb.dobzhanskycenter.org). All of the mutations have been validated experimentally to result in either resistant (positive) or susceptible phenotype (negative). A total of 186 data were finally gathered, including 123 positive and 63 negative mutations (Additional file 1: Table S3).

### Construction of the RpoB mutants

The wild type RpoB in MTB was obtained from Protein Data Bank (ID: 5UHC, Chain C). Mutated RpoB with single point mutations were constructed through PremPS Server (https://lilab.jysw.suda.edu.cn/research/PremPS/), using the wild type RpoB (5UHC) as a template. It should be noticed that the residue numbering in 5UHC was different from the reference sequence of MTB (Fig. 1c) and was adjusted to the reference numbering when constructing the mutants. For the construction of KatG mutants, the crystal structure of KatG from MTB with a PDB ID of 1SJ2 was used as the template. PremPS Server also gives a set of parameters that characterize the mutated proteins, such as the unfolding Gibbs free energy changes (ΔΔG), differences of hydrophobicity scale between mutated and wild type RpoB (ΔOMH), and the solvent-accessible surface area (SASA_pro) [32]. Interpretations of these parameters are listed in Additional file 1: Table S2. In addition to the PremPS-based features, another two parameters were obtained as the attributes of the mutations, i.e. the secondary structure (SS) and Distance. SS is the type of the secondary structure (coil, sheet, helix and turn) where the mutation locates, while Distance denotes the distance between the centers of a residue and the binding site of the wild type protein.

### Molecular docking

The interactions between Rif molecule and mutated RpoB were investigated by LeDock, a molecular docking program [33]. Prior to the docking, the mutated models were prepared by LePro, which added missing hydrogen to the proteins and remove redundant structures. The binding pocket of the mutated protein was set manually as a cube box referring to the Rif location in wild type RpoB, with coordinates of x (151, 169), y (− 9, 9) and z (9, 29). The number of the binding poses was set as 20, which means the process will generate 20 random docking poses. The poses with the highest scores were chosen to represent the optimal binding poses of Rif with the mutated models. In case of the docking of isoniazid and KatG, the binding pocket in KatG was set at x (16, 36), y (− 19, 1) and z (17, 37).

### Machine learning algorithms

Five supervised ML algorithms were employed for developing predictive models in the present research, i.e. NB, kNN, SVM, DT and PNN. NB is a supervised learning algorithm based on Bayes’ theorem with a “naive” assumption that all attributes are independent given the value of the class variable [34, 35]. kNN classifier is based on the Euclidean distance between the target sample and the training samples, where k denotes the number of the nearest neighbors that are used for classifying the target sample [36]. SVM is a statistical learning method that uses a hyperplane to optimally separate data into negative and positive categories [37]. DT predicts the category of a sample by using a tree-like flowchart, where the nodes represent the test on an attribute and the branches denote the outcome of the test [38]. PNN is a pattern classification algorithm, which in the present work was trained using Constructive Training of Probabilistic Neural Networks as the underlying algorithm [39]. All of the five ML algorithms are commonly used for solving classification problems. In the present research, the predictive models using the five ML algorithms were developed on KNIME platform with “Naïve Bayes Learner”, “SVM Learner”, “K Nearest Neighbor”, “Decision Tree Learner” and “PNN Learner (DDA)” nodes, respectively, while the class assignment together with the probability of each class were obtained through “Naïve Bayes Predictor”, “SVM Predictor”, “K Nearest Neighbor”, “Decision Tree Predictor” and “PNN Predictor” nodes, respectively. The node “Partitioning” was used for randomly dividing the data into a training (80%, 148) and a test set (20%, 38) with a stratified sampling method (Additional file 1: Table S3). A ten-fold cross validation was performed for the training set by using a combination of “X-partitioner” and “X-aggregator” nodes. Receiver Operating Characteristic (ROC) curve was generated using a “ROC curve” node. Precision (P), recall (R), specificity (SP) accuracy (AC) and F-measure were calculated and gathered for evaluation of the five classifiers. The functions used for deriving these parameters were as follows.

1 $$P = \frac{TP}{{TP + FP}}$$

2 $$R = \frac{TP}{{TP + FN}}$$

3 $$SP = \frac{TN}{{TN + FP}}$$

4 $$AC = \frac{TP + TN}{{TP + FP + TN + FN}}$$

5 $$F{\text{-}}measure = \frac{2 \times P \times R}{{P + R}}$$

where TP, FP, TN and FN denote true positive, false positive, true negative and false negative respectively.

### Majority consensus

A MC approach was used to obtain a new classifier, which combined the classification results of the five individual ML algorithms. In this approach, a positive prediction was assigned to a mutation when more than 2 of the individual algorithms gave positive predictions, otherwise a negative prediction was assigned to this mutation. A diagram illustrating the workflow for developing the ML classifiers and the MC classifier was depicted in Additional file 1: Fig. S2. The five ML algorithms and the MC algorithm were integrated into a single workflow and were run at the same time to ensure the training and test sets were identical in all of the algorithms. The workflow was provided as an appendix file (Additional file 2) for others to reproduce the present work.

## Supplementary Information

**Additional file 1**. The supporting information file (SuppInfo.docx) contains the docking poses of rifampin in different RpoB models (Fig. S1), a diagram displaying the KNIME workflow (Fig. S2), interpretations of resistance-determining regions (Table S1) and PremPS-obtained features (Table S2), and detailed information of the mutation database, prediction results of the classifiers and confusion matrixes (Tables S3–9). (DOCX 864 KB)

**Additional file 2**. The source file of the KNIME workflow (KNIME_workflow.knwf) for reproducing the work in the present study. (KNWF 198 KB)

## Data Availability

The datasets supporting the conclusions of this article are included within the article (and its additional file).

## Abbreviations

AA: Amino acid
AI: Artificial intelligence
ARGs: Antibiotic resistance genes
AUC: Area under the curve
DNA: Deoxyribonucleic acid
DT: Decision tree
GMTV: Genome-wide Mycobacterium tuberculosis Variation
kNN: K nearest neighbors
MC: Majority consensus
ML: Machine learning
MTB: *Mycobacterium tuberculosis*
NB: Naïve Bayes
NCBI: National Center for Biotechnology Information
PNN: Probabilistic neural network
Rif: Rifampicin
RNA: Ribonucleic acid
RNAP: RNA polymerase
ROC: Receiver operating characteristic
RpoB: RNA polymerase subunit β
RRDRs: Rifampicin resistance-determining regions
SVM: Support vector machine
ΔΔG: Unfolding Gibbs free energy changes

## References

[1] Interagency Coordination Group on Antimicrobial Resistance. No time to wait: securing the future from drug-resistant infections. Report to the secretary-general of the United Nations. 2019.

[2] Gillespie SH (2002). Evolution of drug resistance in *Mycobacterium tuberculosis*: clinical and molecular perspective. Antimicrob Agents Chemother.

[3] Taniguchi H, Aramaki H, Nikaido Y, Mizuguchi Y, Nakamura M, Koga T (1996). Rifampicin resistance and mutation of the *rpoB* gene in *Mycobacterium tuberculosis*. FEMS Microbiol Lett.

[4] MacNeil A. Global Epidemiology of Tuberculosis and Progress Toward Meeting Global Targets—Worldwide, 2018. MMWR Morb Mortal Wkly Rep. 2020;69. 10.15585/mmwr.mm6911a2 PMC7739980 32191687

[5] Partridge SR, Kwong SM, Firth N, Jensen SO (2018). Mobile genetic elements associated with antimicrobial resistance. Clin Microbiol Rev.

[6] Alcalde-Rico M, Hernando-Amado S, Blanco P, Martínez JL (2016). Multidrug efflux pumps at the crossroad between antibiotic resistance and bacterial virulence. Front Microbiol.

[7] Zhang Q, Lambert G, Liao D, Kim H, Robin K, Tung CK (2011). Acceleration of emergence of bacterial antibiotic resistance in connected microenvironments. Science (80–).

[8] Campbell EA, Korzheva N, Mustaev A, Murakami K, Nair S, Goldfarb A (2001). Structural mechanism for rifampicin inhibition of bacterial RNA polymerase. Cell.

[9] Goldstein BP (2014). Resistance to rifampicin: a review. J Antibiot (Tokyo).

[10] Rzeszótko J, Nguyen SH (2012). Machine learning for traffic prediction. Fundam Informaticae.

[11] Padmanabhan J, Johnson Premkumar MJ (2015). Machine learning in automatic speech recognition: a survey. IETE Tech Rev.

[12] Deng L, Li X (2013). Machine learning paradigms for speech recognition: an overview. IEEE Trans Audio Speech Lang Process.

[13] Portugal I, Alencar P, Cowan D (2018). The use of machine learning algorithms in recommender systems: a systematic review. Expert Syst Appl.

[14] Murakami Y, Mizuguchi K (2010). Applying the Naïve Bayes classifier with kernel density estimation to the prediction of protein–protein interaction sites. Bioinformatics.

[15] Zhang H, Yu P, Ren J-X, Li X-B, Wang H-L, Ding L (2017). Development of novel prediction model for drug-induced mitochondrial toxicity by using naïve Bayes classifier method. Food Chem Toxicol.

[16] Zhang H, Ding L, Zou Y, Hu S-Q, Huang H-G, Kong W-B (2016). Predicting drug-induced liver injury in human with Naïve Bayes classifier approach. J Comput Aided Mol Des.

[17] Lahiri N, Shah RR, Layre E, Young D, Ford C, Murray MB (2016). Rifampin resistance mutations are associated with broad chemical remodeling of *Mycobacterium tuberculosis*. J Biol Chem.

[18] Molodtsov V, Scharf NT, Stefan MA, Garcia GA, Murakami KS (2017). Structural basis for rifamycin resistance of bacterial RNA polymerase by the three most clinically important RpoB mutations found in *Mycobacterium tuberculosis*. Mol Microbiol.

[19] Garibyan L, Huang T, Kim M, Wolff E, Nguyen A, Nguyen T (2003). Use of the *rpoB* gene to determine the specificity of base substitution mutations on the *Escherichia coli* chromosome. DNA Repair (Amst).

[20] Sanchez-Padilla E, Merker M, Beckert P, Jochims F, Dlamini T, Kahn P (2015). Detection of drug-resistant tuberculosis by xpert MTB/RIF in Swaziland. N Engl J Med.

[21] Walker TM, Kohl TA, Omar SV, Hedge J, Del Ojo EC, Bradley P (2015). Whole-genome sequencing for prediction of *Mycobacterium tuberculosis* drug susceptibility and resistance: a retrospective cohort study. Lancet Infect Dis.

[22] Miotto P, Tessema B, Tagliani E, Chindelevitch L, Starks AM, Emerson C (2017). A standardised method for interpreting the association between mutations and phenotypic drug resistance in *Mycobacterium tuberculosis*. Eur Respir J.

[23] Zhang Z, Wang L, Gao Y, Zhang J, Zhenirovskyy M, Alexov E (2012). Predicting folding free energy changes upon single point mutations. Bioinformatics.

[24] CRyPTIC Consortium And The GP (2018). Prediction of susceptibility to first-line tuberculosis drugs by DNA sequencing. N Engl J Med.

[25] Woodford N, Ellington MJ (2007). The emergence of antibiotic resistance by mutation. Clin Microbiol Infect.

[26] Mascher T, Heintz M, Zähner D, Merai M, Hakenbeck R (2006). The CiaRH system of streptococcus pneumoniae prevents lysis during stress induced by treatment with cell wall inhibitors and by mutations in *pbp2x* involved in β-lactam resistance. J Bacteriol.

[27] Wu JY, Kim JJ, Reddy R, Wang WM, Graham DY, Kwon DH (2005). Tetracycline-resistant clinical *Helicobacter pylori* isolates with and without mutations in 16S rRNA-encoding genes. Antimicrob Agents Chemother.

[28] Herrera L, Jimenez S, Valverde A, Garcia-Aranda MA, Saez-Nieto JA (2003). Molecular analysis of rifampicin-resistant Mycobacterium tuberculosis isolated in Spain (1996–2001). Description of new mutations in the rpoB gene and review of the literature. Int J Antimicrob Agents.

[29] Lv L, Jiang T, Zhang S, Yu X (2014). Exposure to mutagenic disinfection byproducts leads to increase of antibiotic resistance in *Pseudomonas aeruginosa*. Environ Sci Technol.

[30] Frey KM, Georgiev I, Donald BR, Anderson AC (2010). Predicting resistance mutations using protein design algorithms. Proc Natl Acad Sci.

[31] Jamal S, Khubaib M, Gangwar R, Grover S, Grover A, Hasnain SE. Artificial Intelligence and Machine learning based prediction of resistant and susceptible mutations in *Mycobacterium tuberculosis*. Sci Rep. 2020;10. 10.1038/s41598-020-62368-2 PMC7099008 32218465

[32] Chen Y, Lu H, Zhang N, Zhu Z, Wang S, Li M. PremPS: Predicting the Effects of Single Mutations on Protein Stability. bioRxiv. 2020. 10.1371/journal.pcbi.1008543 PMC7802934 33378330

[33] Wang Z, Sun H, Yao X, Li D, Xu L, Li Y (2016). Comprehensive evaluation of ten docking programs on a diverse set of protein–ligand complexes: the prediction accuracy of sampling power and scoring power. Phys Chem Chem Phys.

[34] Berger JO (2013). Statistical decision theory and Bayesian analysis.

[35] Box GEP, Tiao GC (2011). Bayesian inference in statistical analysis.

[36] Kataria A, Singh MD (2013). A review of data classification using k-nearest neighbour algorithm. Int J Emerg Technol Adv Eng.

[37] Noble WS (2006). What is a support vector machine?. Nat Biotechnol.

[38] Song Y-Y, Ying LU (2015). Decision tree methods: applications for classification and prediction. Shanghai Arch Psychiatry.

[39] Berthold MR, Diamond J (1998). Constructive training of probabilistic neural networks. Neurocomputing.

