# Supplementary material for: Predicting rifampicin resistance mutations in bacterial RNA polymerase subunit beta based on majority consensus
Source: BMC Bioinformatics. 2021 Apr 22;22:210. doi: 10.1186/s12859-021-04137-0 (PMC8063314; doi:10.1186/s12859-021-04137-0)
Supplement: Supplementary file 1 — Additional file 1. The supporting information file (SuppInfo.docx) contains the docking poses of rifampin in different RpoB models (Fig. S1), a diagram displaying the KNIME workflow (Fig. S2), interpretations of resistance-determining regions (Table S1) and PremPS-obtained features (Table S2), and detailed information of the mutation database, prediction results of the classifiers and confusion matrixes (Tables S3–9). (DOCX 864 KB) [file 12859_2021_4137_MOESM1_ESM.docx]

Supporting Information

TITLE: Predicting Rifampicin Resistance Mutations in Bacterial RNA Polymerase Subunit Beta Based on Majority Consensus

AUTHORS: Qing Ning, Dali Wang*, Fei Cheng, Yuheng Zhong, Qi Ding, Jing You

ADDRESS: Guangdong Key Laboratory of Environmental Pollution and Health, School of Environment, Jinan University, Guangzhou 511443, China

JOURNAL: BMC Bioinformatics

NO. OF PAGES: 48

FIGURES: 2

TABLES: 9

^*^Corresponding author.

Tel: 0086-20-3733-6629, Email: [wdali2018@jnu.edu.cn](mailto:wdali2018@jnu.edu.cn)


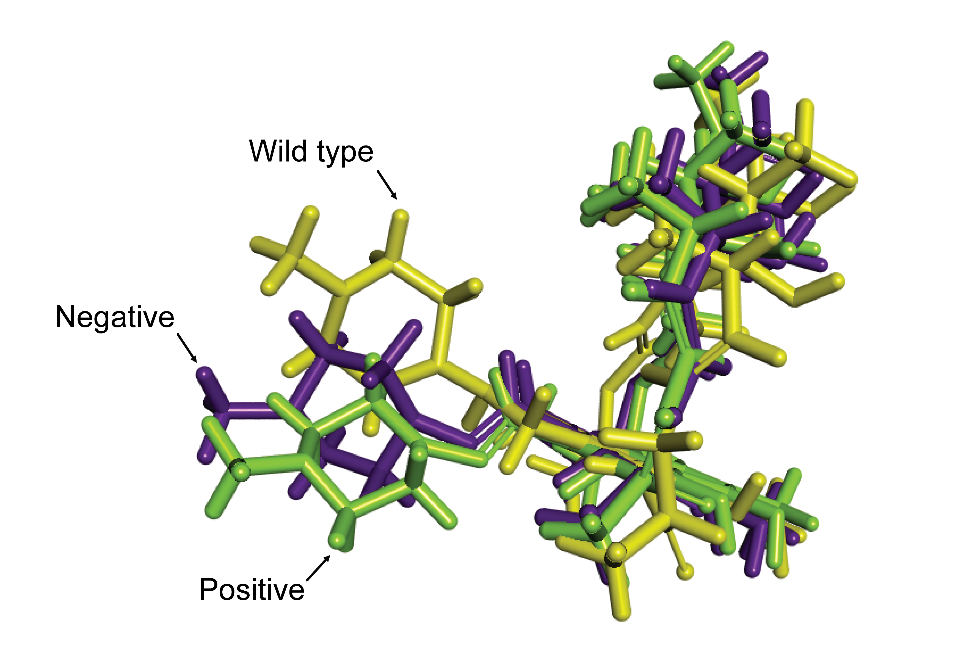


Fig. S1 Comparison of the Rifampin (Rif) poses in the RpoB-Rif complex obtained from the Protein Database (5UHC) and in the complex of mutated negative and positive RpoB and Rif molecule. The yellow pose represents wild type RpoB, the purple and green poses denote the negative mutant F443L and the positive mutant H445D, respectively.


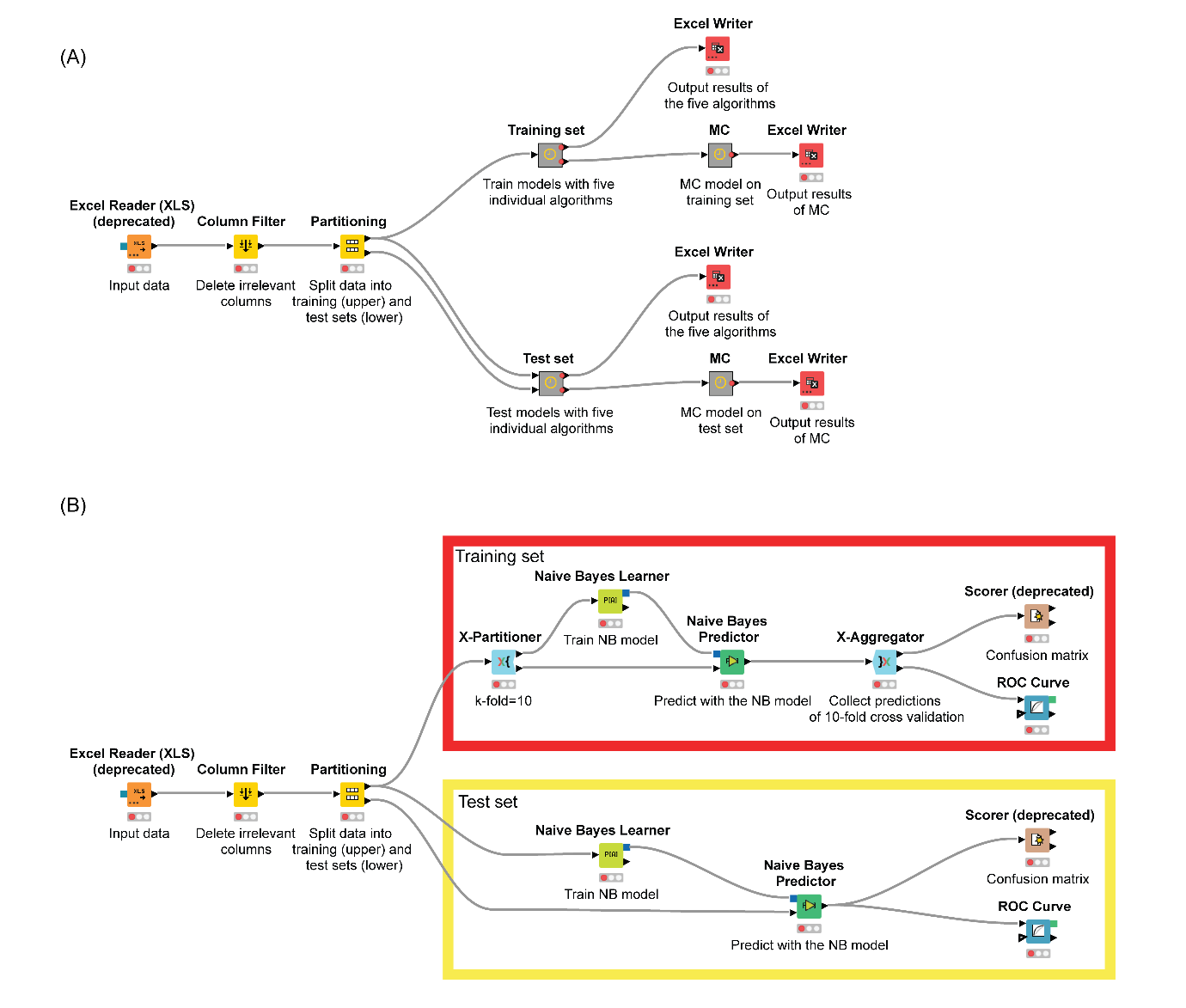


Fig. S2 (A) The overall workflow used for developing the machine learning models and the majority consensus classifier. (B) An example of the workflow for developing individual machine learning classifier. In this example, the naïve Bayes algorithm was displayed.

Table S1. Sequence range of rifampicin resistance-determining regions in *Mycobacterium tuberculosis* RpoB.

| Rifampicin resistance-determining regions（RRDR） | Sequence range |
| --- | --- |
| RRDR-N | 167-172 |
| RRDR-Ⅰ | 423-457 |
| RRDR-Ⅱ | 481-495 |
| RRDR-Ⅲ | 604-611 |

Table S2. Interpretations of the attributes of the mutated proteins

| Feature | Annotation |
| --- | --- |
| PremPS | Quantitative changes in unfolding Gibbs free energy (ΔΔG) |
| Location | There are two values for location, “COR” indicates that the mutated amino acid is buried in the protein core, while “SUR” means the mutated residue locates on the surface of the protein |
| PSSM | Position-Specific Scoring Matrix created by PSI-BLAST |
| ΔCS | Change of conservation after mutation calculated by PROVEAN method |
| ΔOMH | Difference of hydrophobicity scale between mutant and wild-type residue type |
| P_L | Fraction of leucine (L) buried in the protein core |
| P_FWY | Fraction of aromatic residues (F, W or Y) buried in the protein core |
| P_RKDE | Fraction of charged residues (R, K, D or E) buried in the protein core |
| N_Hydro | Number of hydrophobic (V, I, L, F, M, W, Y or C) of 23 sites in the protein sequence, the center of which is at the mutated site |
| N_Charg | Number of charged amino acids (R, K, D or E) of 23 sites in the protein sequence, the center of which is at the mutated site |
| SASA_pro | Solvent accessible surface area (SASA) of the mutated residue in the protein |
| SASA_sol | Solvent accessible surface area (SASA) of the mutated residue in the extended tripeptide |
| Distance | Distance of the central coordinates between amino acid and the active site |
| SS | The secondary structure of the protein where the mutated amino acid locates. There are four values of SS, i.e., coil, sheet, helix and turn. |

https://lilab.jysw.suda.edu.cn/research/PremPS/method/

Table S3. The detailed parameters for the *Mycobacterium tuberculosis* RpoB mutants in the training and test datasets. P: positive, N: negative.

| Mutation | Distance | SS | Location | PremPS | PSSM | DCS | DOMH | P_L | P_FWY | P_RKDE | N_Hydro | N_Charg | SASA_pro | SASA_sol | DE | ΔE | Type |
| --- | --- | --- | --- | --- | --- | --- | --- | --- | --- | --- | --- | --- | --- | --- | --- | --- | --- |
| Training set | | | | | | | | | | | | | | | | | |
| Q172L | 13.45 | 2 | 1 | 0.97 | 0.47 | 0.71 | -0.86 | -0.02 | -0.11 | 0.15 | 0.09 | 0.00 | 0.44 | 0.10 | -8.8 | 0.08 | P |
| V359A | 40.64 | 1 | 1 | 0.94 | 0.15 | 0.21 | 0.55 | 0.01 | -0.20 | -0.15 | 0.01 | -0.01 | 0.30 | 0.07 | -8.9 | -0.02 | P |
| D435N | 9.48 | 1 | 1 | 0.46 | 0.40 | 0.74 | -0.22 | -0.01 | -0.18 | 0.03 | -0.11 | -0.05 | 0.04 | -0.19 | -7.74 | 1.14 | P |
| H445N | 10.17 | 3 | 1 | 1.2 | -0.03 | 1.04 | 0.02 | -0.01 | -0.21 | 0.01 | -0.01 | -0.04 | 0.31 | 0.13 | -8.07 | 0.81 | P |
| H445Y | 10.17 | 3 | 1 | 0.74 | 0.31 | 0.87 | -0.91 | 0.00 | -0.14 | 0.10 | 0.10 | -0.04 | 0.31 | 0.16 | -6.87 | 2.01 | P |
| S450L | 10.16 | 2 | 1 | 0.77 | 0.64 | 0.98 | -0.71 | 0.03 | -0.16 | 0.09 | 0.10 | -0.02 | 0.06 | -0.23 | -7.12 | 1.76 | P |
| S450F | 10.16 | 2 | 1 | 0.68 | 0.63 | 0.98 | -0.80 | 0.03 | -0.15 | 0.08 | 0.10 | -0.03 | 0.07 | -0.23 | -7.56 | 1.32 | P |
| S493F | 14.12 | 2 | 1 | 0.71 | 0.64 | 0.93 | -0.80 | 0.03 | -0.15 | 0.14 | -0.07 | -0.06 | 0.26 | -0.21 | -7.28 | 1.6 | P |
| S493Y | 14.12 | 2 | 1 | 0.42 | -0.03 | 1.44 | -0.84 | 0.03 | -0.16 | 0.12 | -0.05 | -0.01 | 0.14 | -0.21 | -8.81 | 0.07 | P |
| T676P | 18.65 | 1 | 1 | 1.11 | 0.55 | 0.70 | -0.01 | -0.01 | -0.24 | 0.02 | -0.02 | 0.00 | 0.30 | -0.17 | -8.87 | 0.01 | P |
| G981D | 46.05 | 3 | 1 | 0.6 | -0.47 | 0.97 | 0.42 | 0.05 | -0.20 | 0.04 | 0.00 | 0.00 | 0.08 | -0.29 | -8.82 | 0.06 | P |
| L42F | 33.37 | 1 | 0 | 0.67 | 0.30 | 0.63 | -0.31 | 0.01 | -0.21 | 0.04 | -0.03 | 0.00 | 0.10 | 0.14 | -8.89 | -0.01 | P |
| L47R | 21.46 | 4 | 1 | 2.64 | 0.74 | 0.64 | 0.85 | -0.06 | -0.21 | -0.07 | 0.02 | 0.00 | 0.57 | 0.16 | -8.84 | 0.04 | P |
| E82G | 31.51 | 3 | 1 | 1.18 | 0.59 | 0.68 | -0.31 | -0.05 | -0.28 | 0.05 | 0.09 | 0.02 | 0.25 | 0.14 | -8.87 | 0.01 | P |
| I90L | 40.34 | 2 | 1 | 1.33 | 0.53 | 0.39 | -0.13 | -0.01 | -0.17 | 0.05 | 0.11 | 0.04 | 0.43 | 0.10 | -8.91 | -0.03 | P |
| V170F | 10.51 | 1 | 1 | 1.48 | 0.63 | 0.75 | -0.58 | 0.00 | -0.18 | 0.12 | 0.12 | -0.05 | 0.59 | 0.08 | -7.72 | 1.16 | P |
| V170G | 10.51 | 1 | 1 | 2.83 | 0.84 | 0.72 | 0.84 | -0.05 | -0.22 | -0.02 | 0.06 | -0.06 | 0.56 | 0.16 | -7.32 | 1.56 | P |
| Q172H | 13.45 | 2 | 1 | 1.84 | 0.63 | 0.72 | -0.37 | 0.00 | -0.20 | 0.11 | 0.18 | 0.03 | 0.60 | 0.17 | -8.79 | 0.09 | P |
| Q172K | 13.45 | 2 | 1 | 2.07 | 0.61 | 0.75 | -0.23 | 0.00 | -0.18 | 0.10 | 0.23 | 0.04 | 0.60 | 0.15 | -8.9 | -0.02 | P |
| Q172P | 13.45 | 2 | 1 | 1.77 | 0.62 | 0.73 | -0.36 | -0.01 | -0.19 | 0.09 | 0.14 | 0.02 | 0.57 | 0.16 | -8.89 | -0.01 | P |
| Q172R | 13.45 | 2 | 1 | 1.88 | 0.60 | 0.76 | -0.37 | 0.01 | -0.19 | 0.10 | 0.19 | 0.02 | 0.61 | 0.16 | -8.91 | -0.03 | P |
| D265G | 54.99 | 3 | 0 | 0.51 | 0.52 | 0.64 | -0.23 | -0.01 | -0.20 | 0.10 | -0.07 | 0.00 | -0.07 | -0.16 | -8.85 | 0.03 | P |
| P280S | 50.55 | 1 | 0 | 0.73 | 0.53 | 0.70 | 0.05 | 0.02 | -0.23 | 0.09 | -0.01 | 0.00 | -0.34 | -0.06 | -8.87 | 0.01 | P |
| L378R | 17.7 | 3 | 1 | 2.17 | 0.49 | 0.74 | 0.86 | -0.06 | -0.28 | -0.06 | -0.05 | 0.00 | 0.38 | 0.15 | -8.85 | 0.03 | P |
| T399A | 46.55 | 3 | 0 | 0.47 | 0.26 | 0.72 | 0.03 | 0.00 | -0.25 | 0.06 | -0.03 | -0.02 | -0.22 | -0.07 | -8.8 | 0.08 | P |
| T400I | 46.85 | 3 | 0 | -0.52 | -0.52 | 0.68 | -0.66 | -0.01 | -0.04 | -0.04 | 0.02 | -0.04 | 0.16 | -0.07 | -8.84 | 0.04 | P |
| F424L | 15.97 | 3 | 1 | 1.24 | 0.42 | 0.71 | 0.08 | -0.03 | -0.33 | -0.07 | 0.05 | -0.05 | 0.28 | 0.19 | -8.83 | 0.05 | P |
| G426D | 14.49 | 3 | 0 | 0.76 | 0.52 | 0.82 | 0.10 | 0.00 | -0.37 | 0.04 | -0.03 | -0.02 | -0.08 | -0.21 | -8.89 | -0.01 | P |
| T427P | 13.02 | 3 | 0 | 0.57 | 0.52 | 0.66 | 0.00 | 0.00 | -0.28 | 0.10 | 0.01 | -0.10 | -0.31 | -0.04 | -8.87 | 0.01 | P |
| L430P | 9.16 | 4 | 1 | 2.31 | 0.69 | 0.78 | 0.83 | -0.07 | -0.31 | -0.03 | -0.01 | -0.12 | 0.41 | 0.14 | -8.83 | 0.05 | P |
| L430R | 9.16 | 4 | 1 | 2.02 | 0.50 | 0.72 | 0.84 | -0.08 | -0.32 | -0.08 | 0.03 | -0.11 | 0.38 | 0.14 | -8.78 | 0.1 | P |
| S431A | 10.1 | 2 | 1 | 0.76 | 0.52 | 0.78 | -0.05 | 0.03 | -0.23 | 0.00 | -0.10 | -0.11 | 0.16 | -0.25 | -8.84 | 0.04 | P |
| S431F | 10.1 | 2 | 1 | 0.57 | 0.63 | 0.96 | -0.76 | 0.04 | -0.15 | 0.12 | -0.06 | -0.07 | 0.07 | -0.19 | -8.72 | 0.16 | P |
| S431P | 10.1 | 2 | 1 | 0.72 | 0.58 | 0.76 | -0.04 | 0.00 | -0.25 | 0.02 | -0.10 | -0.16 | 0.15 | -0.24 | -8.86 | 0.02 | P |
| S431R | 10.1 | 2 | 1 | 0.74 | 0.58 | 0.76 | -0.01 | 0.01 | -0.27 | 0.01 | -0.09 | -0.17 | 0.16 | -0.25 | -8.91 | -0.03 | P |
| S431T | 10.1 | 2 | 1 | 0.52 | 0.32 | 0.82 | -0.16 | 0.03 | -0.20 | 0.00 | -0.08 | -0.09 | 0.14 | -0.25 | -8.87 | 0.01 | P |
| Q432K | 7.4 | 2 | 0 | 0.62 | 0.40 | 0.66 | -0.13 | 0.02 | -0.31 | 0.05 | -0.03 | -0.06 | -0.08 | 0.09 | -8.51 | 0.37 | P |
| Q432L | 7.4 | 2 | 0 | 0.58 | 0.55 | 0.74 | -0.76 | 0.01 | -0.21 | 0.15 | 0.00 | -0.01 | 0.04 | 0.07 | -8.33 | 0.55 | P |
| Q432R | 7.4 | 2 | 0 | 0.6 | 0.40 | 0.67 | -0.20 | 0.02 | -0.29 | 0.04 | -0.02 | -0.04 | -0.06 | 0.09 | -6.88 | 2 | P |
| D435A | 9.48 | 1 | 1 | 0.45 | 0.51 | 0.74 | -0.48 | 0.02 | -0.08 | 0.11 | -0.07 | -0.07 | 0.01 | -0.24 | -8.55 | 0.33 | P |
| D435F | 9.48 | 1 | 1 | 0.44 | 0.46 | 0.87 | -0.71 | 0.01 | -0.05 | 0.11 | -0.06 | -0.05 | 0.06 | -0.20 | -6.73 | 2.15 | P |
| D435G | 9.48 | 1 | 1 | 0.55 | 0.57 | 0.78 | -0.36 | 0.02 | -0.12 | 0.08 | -0.09 | -0.09 | 0.01 | -0.26 | -7.04 | 1.84 | P |
| D435V | 9.48 | 1 | 1 | 0.48 | 0.45 | 0.90 | -0.73 | 0.02 | -0.04 | 0.13 | -0.06 | -0.03 | 0.05 | -0.20 | -7.36 | 1.52 | P |
| D435Y | 9.48 | 1 | 1 | 0.46 | 0.46 | 0.88 | -0.70 | 0.02 | -0.05 | 0.11 | -0.06 | -0.05 | 0.07 | -0.20 | -6.44 | 2.44 | P |
| N437D | 14.22 | 1 | 1 | 0.99 | 0.40 | 0.65 | -0.02 | -0.01 | -0.21 | -0.03 | 0.00 | -0.01 | 0.13 | 0.09 | -8.93 | -0.05 | P |
| S441F | 13.15 | 3 | 1 | 0.97 | 0.72 | 0.90 | -0.80 | 0.06 | -0.15 | 0.12 | -0.10 | 0.04 | 0.40 | -0.21 | -7.67 | 1.21 | P |
| S441L | 13.15 | 3 | 1 | 0.73 | 0.67 | 0.73 | -0.81 | 0.06 | -0.13 | 0.10 | -0.09 | 0.05 | 0.35 | -0.21 | -7.57 | 1.31 | P |
| S441Q | 13.15 | 3 | 1 | 1.24 | 0.64 | 0.56 | 0.08 | 0.04 | -0.22 | 0.02 | -0.08 | -0.01 | 0.47 | -0.27 | -7.77 | 1.11 | P |
| S441Y | 13.15 | 3 | 1 | 1.01 | 0.67 | 0.91 | -0.76 | 0.06 | -0.15 | 0.13 | -0.10 | 0.05 | 0.42 | -0.23 | -7.46 | 1.42 | P |
| H445C | 10.17 | 3 | 1 | 1.09 | 0.52 | 0.81 | -0.58 | -0.04 | -0.20 | 0.10 | 0.05 | -0.04 | 0.33 | 0.15 | -8.34 | 0.54 | P |
| H445D | 10.17 | 3 | 1 | 1.48 | 0.56 | 0.77 | 0.10 | -0.06 | -0.35 | -0.01 | -0.01 | -0.03 | 0.36 | 0.18 | -8.35 | 0.53 | P |
| H445F | 10.17 | 3 | 1 | 0.87 | 0.59 | 0.82 | -0.97 | 0.00 | -0.19 | 0.13 | 0.06 | -0.03 | 0.30 | 0.14 | -7.51 | 1.37 | P |
| H445G | 10.17 | 3 | 1 | 1.38 | 0.61 | 0.73 | -0.16 | -0.07 | -0.27 | 0.04 | 0.00 | -0.03 | 0.32 | 0.21 | -8.61 | 0.27 | P |
| H445P | 10.17 | 3 | 1 | 1.33 | 0.60 | 0.75 | -0.24 | -0.07 | -0.26 | 0.05 | 0.00 | -0.03 | 0.32 | 0.21 | -7.27 | 1.61 | P |
| H445R | 10.17 | 3 | 1 | 1.34 | 0.56 | 0.72 | -0.18 | -0.07 | -0.25 | 0.06 | 0.01 | -0.03 | 0.34 | 0.19 | -7 | 1.88 | P |
| R448C | 11.16 | 2 | 0 | 0.81 | 0.49 | 0.70 | -0.41 | 0.00 | -0.26 | 0.09 | 0.02 | -0.03 | -0.05 | 0.25 | -8.44 | 0.44 | P |
| R448L | 11.16 | 2 | 0 | 0.81 | 0.51 | 0.74 | -0.68 | 0.02 | -0.23 | 0.15 | 0.02 | 0.00 | 0.01 | 0.26 | -8.48 | 0.4 | P |
| R448S | 11.16 | 2 | 0 | 0.94 | 0.51 | 0.66 | -0.06 | -0.01 | -0.36 | 0.04 | 0.01 | -0.03 | -0.11 | 0.29 | -8.7 | 0.18 | P |
| S450P | 10.16 | 2 | 1 | 1.06 | 0.59 | 0.79 | -0.05 | 0.03 | -0.25 | 0.01 | 0.07 | 0.01 | 0.15 | -0.28 | -8.66 | 0.22 | P |
| S450W | 10.16 | 2 | 1 | 0.84 | 0.58 | 0.90 | -0.47 | 0.01 | -0.18 | 0.06 | 0.10 | -0.02 | 0.08 | -0.23 | -6.59 | 2.29 | P |
| S450Y | 10.16 | 2 | 1 | 0.73 | 0.64 | 0.98 | -0.76 | 0.03 | -0.15 | 0.09 | 0.10 | -0.02 | 0.07 | -0.23 | -6.89 | 1.99 | P |
| A451E | 12.31 | 1 | 1 | 1.67 | 0.61 | 0.79 | 0.16 | 0.06 | -0.27 | -0.01 | 0.06 | 0.02 | 0.49 | -0.23 | -8.84 | 0.04 | P |
| A451V | 12.31 | 1 | 1 | 1.81 | 0.59 | 1.01 | -0.41 | 0.16 | -0.14 | 0.11 | 0.16 | 0.00 | 0.48 | -0.13 | -8.85 | 0.03 | P |
| L452H | 10.2 | 1 | 1 | 1.88 | 0.52 | 0.76 | 0.86 | -0.04 | -0.35 | 0.02 | -0.01 | -0.01 | -0.02 | 0.15 | -7.08 | 1.8 | P |
| L452P | 10.2 | 1 | 1 | 2.17 | 0.73 | 0.74 | 0.85 | -0.01 | -0.31 | 0.04 | 0.02 | 0.00 | -0.03 | 0.14 | -8.65 | 0.23 | P |
| L452R | 10.2 | 1 | 1 | 1.81 | 0.52 | 0.67 | 0.86 | -0.03 | -0.34 | 0.02 | -0.01 | -0.01 | -0.02 | 0.15 | -8.35 | 0.53 | P |
| G453C | 11.05 | 1 | 1 | 1.14 | 0.66 | 0.92 | -0.42 | 0.06 | -0.16 | 0.06 | 0.07 | 0.00 | 0.08 | -0.13 | -9 | -0.12 | P |
| G453D | 11.05 | 1 | 1 | 1.33 | 0.60 | 0.93 | 0.17 | 0.01 | -0.31 | -0.01 | 0.04 | 0.04 | 0.20 | -0.33 | -8.82 | 0.06 | P |
| G453S | 11.05 | 1 | 1 | 1.16 | 0.59 | 0.87 | -0.07 | 0.05 | -0.25 | 0.02 | 0.06 | 0.01 | 0.13 | -0.26 | -8.87 | 0.01 | P |
| G456S | 15.49 | 1 | 0 | 1.05 | 0.54 | 0.86 | -0.06 | 0.03 | -0.29 | 0.06 | 0.06 | -0.01 | 0.06 | -0.21 | -8.88 | 0 | P |
| P483L | 10.39 | 1 | 0 | 0.7 | 0.50 | 0.96 | -0.66 | -0.01 | -0.14 | 0.15 | -0.02 | -0.05 | 0.00 | -0.03 | -7.24 | 1.64 | P |
| P483R | 10.39 | 1 | 0 | 0.99 | 0.53 | 0.89 | -0.01 | -0.04 | -0.30 | 0.07 | -0.04 | -0.13 | 0.06 | -0.05 | -7.73 | 1.15 | P |
| I488V | 12.57 | 4 | 0 | 0.5 | -0.08 | 0.60 | 0.13 | -0.05 | -0.15 | 0.05 | 0.00 | -0.02 | -0.11 | 0.13 | -8.89 | -0.01 | P |
| G489C | 14.36 | 4 | 1 | 1.1 | 0.62 | 1.05 | -0.40 | 0.01 | -0.16 | 0.10 | 0.09 | -0.13 | 0.03 | -0.11 | -8.84 | 0.04 | P |
| I491F | 10.24 | 2 | 1 | 0.9 | 0.57 | 0.69 | -0.36 | -0.03 | -0.24 | 0.04 | -0.04 | -0.07 | 0.18 | 0.16 | -7.18 | 1.7 | P |
| I491M | 10.24 | 2 | 1 | 0.71 | 0.28 | 0.66 | -0.10 | -0.03 | -0.27 | -0.02 | -0.02 | -0.04 | 0.16 | 0.08 | -8.46 | 0.42 | P |
| I491T | 10.24 | 2 | 1 | 1.37 | 0.45 | 0.60 | 0.76 | -0.02 | -0.38 | -0.01 | -0.02 | -0.17 | 0.02 | 0.15 | -8.5 | 0.38 | P |
| I491V | 10.24 | 2 | 1 | 0.78 | -0.02 | 0.61 | 0.11 | -0.01 | -0.16 | 0.03 | -0.01 | -0.03 | 0.11 | 0.15 | -8.89 | -0.01 | P |
| V496L | 22.4 | 1 | 1 | 0.82 | 0.53 | 0.22 | -0.30 | 0.00 | -0.11 | 0.02 | 0.09 | -0.04 | 0.38 | 0.04 | -8.83 | 0.05 | P |
| V496M | 22.4 | 1 | 1 | 0.71 | 0.59 | -0.06 | -0.18 | -0.01 | -0.13 | 0.10 | 0.04 | -0.03 | 0.33 | 0.06 | -8.82 | 0.06 | P |
| F503S | 26.74 | 4 | 0 | 1.43 | 0.56 | 0.65 | 0.87 | -0.03 | -0.45 | 0.02 | 0.01 | -0.12 | -0.18 | 0.09 | -8.86 | 0.02 | P |
| P551S | 49.37 | 1 | 0 | -0.2 | 0.36 | -0.51 | 0.03 | -0.04 | -0.08 | 0.00 | 0.02 | -0.06 | 0.08 | -0.01 | -8.87 | 0.01 | P |
| D571A | 41.2 | 1 | 0 | 1.13 | 0.59 | 0.88 | -0.30 | -0.05 | -0.16 | 0.12 | 0.15 | 0.01 | 0.00 | -0.12 | -8.85 | 0.03 | P |
| D574E | 33.51 | 2 | 1 | 1.36 | 0.32 | 0.80 | -0.12 | 0.09 | -0.13 | 0.00 | 0.06 | 0.04 | 0.50 | -0.20 | -8.79 | 0.09 | P |
| R607H | 13.52 | 4 | 1 | 1.2 | 0.56 | 0.70 | -0.02 | -0.01 | -0.39 | 0.07 | -0.03 | -0.02 | -0.01 | 0.34 | -7.53 | 1.35 | P |
| N658D | 47.54 | 1 | 0 | -0.42 | -0.47 | -0.61 | 0.19 | -0.08 | 0.07 | -0.09 | 0.05 | 0.03 | 0.34 | 0.15 | -8.85 | 0.03 | P |
| A670D | 23.74 | 2 | 0 | 0.53 | 0.53 | 0.18 | 0.26 | 0.02 | -0.23 | 0.01 | 0.00 | 0.02 | -0.06 | -0.20 | -8.85 | 0.03 | P |
| C681W | 31.52 | 2 | 0 | 0.18 | 0.61 | -0.10 | -0.22 | 0.00 | -0.11 | 0.01 | 0.00 | 0.03 | -0.01 | -0.01 | -8.83 | 0.05 | P |
| M707T | 24.09 | 1 | 1 | 1.75 | 0.48 | 0.60 | 0.41 | -0.03 | -0.28 | -0.01 | 0.02 | 0.04 | 0.38 | 0.14 | -8.88 | 0 | P |
| H723D | 34.87 | 4 | 0 | 0.94 | 0.64 | 0.64 | 0.07 | -0.03 | -0.41 | 0.06 | 0.10 | -0.07 | -0.14 | 0.09 | -8.87 | 0.01 | P |
| L731P | 32.77 | 2 | 1 | 2.49 | 0.57 | 0.68 | 0.84 | -0.06 | -0.22 | -0.09 | 0.02 | 0.01 | 0.59 | 0.15 | -8.79 | 0.09 | P |
| L735Q | 33.15 | 3 | 1 | 2.46 | 0.50 | 0.67 | 0.86 | -0.07 | -0.23 | -0.06 | 0.08 | -0.02 | 0.59 | 0.14 | -8.8 | 0.08 | P |
| H745Y | 22.16 | 2 | 1 | 0.74 | 0.30 | 0.87 | -0.89 | -0.03 | -0.16 | 0.11 | -0.06 | 0.11 | 0.34 | 0.14 | -8.87 | 0.01 | P |
| E761D | 37.22 | 1 | 1 | 0.92 | 0.27 | 0.67 | -0.12 | -0.03 | -0.24 | -0.03 | 0.02 | 0.06 | 0.22 | 0.11 | -8.88 | 0 | P |
| R827C | 40.01 | 2 | 0 | 0.62 | 0.53 | 0.63 | -0.34 | 0.01 | -0.20 | 0.14 | 0.00 | 0.02 | -0.35 | 0.18 | -8.83 | 0.05 | P |
| H835R | 26.63 | 4 | 0 | 0.54 | 0.52 | 0.57 | -0.04 | 0.01 | -0.23 | 0.11 | -0.02 | 0.04 | -0.47 | 0.06 | -8.85 | 0.03 | P |
| I925V | 33.22 | 3 | 1 | -0.33 | -0.45 | -0.09 | 0.34 | -0.06 | -0.04 | -0.04 | 0.04 | -0.06 | -0.14 | 0.19 | -8.9 | -0.02 | P |
| E978D | 42.85 | 3 | 1 | 0.09 | -0.61 | 0.66 | -0.05 | 0.00 | -0.14 | 0.02 | -0.01 | -0.02 | 0.08 | 0.16 | -8.88 | 0 | P |
| N24D | 33.97 | 1 | 1 | 0.5 | 0.48 | -0.07 | 0.05 | 0.00 | -0.14 | 0.03 | 0.05 | -0.04 | 0.06 | 0.08 | -8.87 | 0.01 | N |
| G28R | 36.91 | 1 | 0 | 0.48 | 0.58 | 0.37 | -0.01 | 0.02 | -0.17 | 0.08 | 0.01 | -0.09 | -0.08 | -0.22 | -8.8 | 0.08 | N |
| P30S | 36.12 | 1 | 0 | -0.1 | 0.00 | -0.05 | 0.06 | -0.04 | -0.06 | -0.01 | 0.01 | 0.01 | 0.03 | -0.06 | -8.87 | 0.01 | N |
| E66K | 35.82 | 3 | 0 | -0.29 | -0.06 | 0.48 | -0.25 | -0.13 | -0.10 | -0.05 | -0.01 | 0.00 | -0.27 | 0.10 | -8.81 | 0.07 | N |
| A69P | 37.64 | 3 | 0 | 0.4 | 0.31 | 0.37 | 0.04 | 0.00 | -0.15 | 0.03 | 0.00 | 0.02 | -0.04 | -0.18 | -8.83 | 0.05 | N |
| V77M | 33.42 | 1 | 0 | 0.05 | 0.35 | -0.06 | -0.07 | 0.01 | -0.14 | -0.04 | -0.01 | -0.02 | -0.01 | 0.04 | -8.85 | 0.03 | N |
| L80V | 29.14 | 3 | 1 | 0.96 | 0.43 | 0.62 | -0.07 | -0.02 | -0.24 | 0.01 | -0.05 | 0.01 | 0.13 | 0.14 | -8.83 | 0.05 | N |
| P89L | 40.69 | 1 | 0 | 0.89 | 0.54 | 0.86 | -0.53 | -0.03 | -0.15 | 0.15 | 0.13 | 0.03 | -0.07 | -0.04 | -8.84 | 0.04 | N |
| V113I | 24.84 | 3 | 0 | -0.42 | -0.51 | 0.45 | -0.20 | -0.01 | -0.10 | -0.04 | -0.01 | -0.09 | 0.04 | 0.04 | -8.86 | 0.02 | N |
| M121I | 23.73 | 1 | 0 | -0.31 | 0.02 | -0.41 | 0.02 | -0.08 | -0.08 | -0.01 | 0.01 | -0.04 | 0.09 | 0.17 | -8.84 | 0.04 | N |
| E132D | 44.24 | 2 | 0 | 0.5 | 0.52 | 0.36 | -0.05 | 0.00 | -0.27 | 0.09 | -0.01 | -0.07 | -0.15 | 0.07 | -8.87 | 0.01 | N |
| M153T | 22.99 | 1 | 1 | 1.21 | 0.53 | 0.62 | 0.40 | -0.03 | -0.39 | 0.03 | 0.02 | -0.08 | -0.04 | 0.17 | -8.85 | 0.03 | N |
| V179A | 32.54 | 2 | 1 | 1.39 | 0.04 | 0.67 | 0.51 | 0.02 | -0.21 | -0.10 | 0.01 | -0.03 | 0.39 | 0.10 | -8.91 | -0.03 | N |
| S195R | 36.51 | 2 | 1 | 0.5 | 0.58 | 0.57 | 0.00 | 0.01 | -0.28 | -0.01 | -0.08 | 0.01 | -0.03 | -0.24 | -8.84 | 0.04 | N |
| D270E | 49.39 | 3 | 1 | -0.41 | -0.48 | 0.22 | 0.11 | -0.07 | -0.06 | -0.05 | 0.00 | -0.01 | -0.09 | 0.02 | -8.81 | 0.07 | N |
| L316V | 42.81 | 1 | 1 | 1.03 | 0.28 | 0.35 | 0.00 | -0.01 | -0.20 | 0.04 | -0.02 | 0.03 | 0.34 | 0.21 | -8.88 | 0 | N |
| H343Q | 43.99 | 3 | 1 | 0.87 | -0.38 | 1.04 | 0.09 | 0.03 | -0.24 | 0.05 | 0.05 | -0.03 | 0.11 | 0.16 | -8.82 | 0.06 | N |
| T350I | 47.39 | 2 | 0 | -0.19 | 0.27 | 0.23 | -0.56 | -0.01 | -0.09 | 0.03 | -0.01 | 0.04 | -0.07 | 0.00 | -8.81 | 0.07 | N |
| P358L | 41.03 | 2 | 0 | -0.14 | 0.41 | 0.29 | -0.68 | 0.02 | -0.09 | 0.01 | -0.02 | 0.03 | -0.08 | -0.01 | -8.85 | 0.03 | N |
| S388L | 30.5 | 3 | 0 | 0.67 | 0.57 | 0.81 | -0.54 | -0.02 | -0.18 | 0.12 | 0.10 | 0.00 | 0.03 | -0.22 | -8.84 | 0.04 | N |
| M390T | 32.53 | 3 | 1 | 1.71 | 0.50 | 0.66 | 0.42 | -0.05 | -0.31 | -0.05 | -0.05 | 0.02 | 0.44 | 0.13 | -8.83 | 0.05 | N |
| L443F | 15.32 | 3 | 1 | 1.16 | 0.51 | 0.73 | -0.50 | -0.01 | -0.20 | 0.05 | -0.04 | -0.02 | 0.50 | 0.16 | -8.86 | 0.02 | N |
| R511L | 36.93 | 2 | 1 | 0.8 | 0.54 | 0.76 | -0.78 | -0.04 | -0.21 | 0.14 | 0.09 | 0.00 | 0.07 | 0.24 | -8.9 | -0.02 | N |
| D515Y | 48.23 | 1 | 0 | 0.33 | 0.54 | 0.65 | -0.56 | -0.04 | -0.09 | 0.16 | 0.09 | 0.01 | -0.31 | -0.11 | -8.82 | 0.06 | N |
| T526S | 28.91 | 1 | 1 | 0.92 | -0.06 | 1.04 | 0.10 | 0.02 | -0.21 | 0.00 | -0.04 | 0.00 | 0.20 | -0.14 | -8.85 | 0.03 | N |
| A544V | 48.7 | 1 | 0 | 0.21 | 0.54 | 0.34 | -0.40 | 0.02 | -0.10 | 0.06 | -0.02 | 0.02 | -0.07 | -0.18 | -8.88 | 0 | N |
| D545A | 49.63 | 1 | 0 | 0.3 | 0.51 | 0.64 | -0.31 | -0.02 | -0.16 | 0.14 | -0.01 | -0.01 | -0.35 | -0.14 | -8.86 | 0.02 | N |
| E563D | 40.84 | 1 | 0 | 0.2 | -0.05 | 0.46 | 0.04 | -0.10 | -0.12 | 0.00 | 0.01 | 0.00 | -0.15 | 0.11 | -8.87 | 0.01 | N |
| D634G | 22.27 | 1 | 1 | 0.45 | 0.57 | 0.55 | -0.35 | 0.03 | -0.11 | 0.02 | -0.05 | -0.05 | 0.12 | -0.27 | -8.89 | -0.01 | N |
| E639G | 34.79 | 1 | 0 | 0.45 | 0.49 | 0.63 | -0.23 | -0.03 | -0.24 | 0.09 | -0.03 | 0.03 | -0.34 | 0.09 | -8.82 | 0.06 | N |
| H674Q | 13.68 | 4 | 0 | -0.51 | -0.46 | -0.66 | 0.19 | -0.11 | 0.07 | -0.08 | 0.06 | 0.01 | 0.31 | 0.16 | -8.87 | 0.01 | N |
| P682T | 34.33 | 2 | 0 | 0.98 | 0.53 | 0.65 | -0.12 | 0.01 | -0.27 | 0.08 | 0.07 | 0.05 | 0.08 | -0.10 | -8.85 | 0.03 | N |
| V695L | 32.19 | 2 | 1 | 0.56 | -0.01 | 0.66 | -0.19 | 0.02 | -0.13 | -0.05 | 0.04 | -0.02 | 0.20 | 0.04 | -8.83 | 0.05 | N |
| P834L | 25.89 | 1 | 0 | 0.77 | 0.51 | 0.83 | -0.57 | -0.01 | -0.15 | 0.13 | 0.00 | 0.01 | 0.08 | -0.05 | -8.88 | 0 | N |
| D851G | 39.24 | 4 | 0 | 0.28 | 0.40 | 0.50 | -0.21 | -0.02 | -0.12 | 0.05 | -0.03 | -0.01 | -0.17 | -0.11 | -8.84 | 0.04 | N |
| A857T | 32.34 | 4 | 0 | 0.24 | 0.51 | 0.04 | -0.04 | 0.02 | -0.12 | 0.02 | 0.06 | 0.00 | -0.06 | -0.18 | -8.82 | 0.06 | N |
| G890D | 30.15 | 2 | 0 | 0.71 | 0.62 | 0.26 | 0.25 | 0.02 | -0.23 | 0.04 | 0.05 | 0.02 | -0.06 | -0.25 | -8.87 | 0.01 | N |
| L893R | 37.74 | 2 | 1 | 2.24 | 0.46 | 0.74 | 0.86 | -0.08 | -0.28 | -0.08 | -0.02 | -0.01 | 0.51 | 0.15 | -8.89 | -0.01 | N |
| K944E | 60.24 | 4 | 0 | 0 | 0.01 | -0.53 | 0.20 | -0.03 | 0.00 | 0.02 | 0.00 | -0.02 | 0.22 | 0.14 | -8.89 | -0.01 | N |
| S1124A | 37.74 | 4 | 0 | 0.26 | -0.35 | 0.93 | 0.02 | 0.02 | -0.15 | 0.05 | 0.03 | 0.02 | -0.08 | -0.22 | -8.85 | 0.03 | N |
| V1129A | 43.14 | 1 | 0 | 1.18 | 0.47 | 0.60 | 0.52 | -0.02 | -0.36 | 0.00 | 0.01 | 0.03 | -0.13 | 0.06 | -8.8 | 0.08 | N |
| P233Q | 46.94 | 2 | 0 | 0.9 | 0.54 | 0.66 | 0.09 | -0.02 | -0.34 | 0.03 | 0.08 | 0.03 | -0.08 | -0.07 | -8.84 | 0.04 | N |
| G374S | 26.93 | 4 | 1 | 0.98 | 0.35 | 0.94 | -0.05 | 0.07 | -0.21 | 0.02 | -0.11 | 0.02 | 0.26 | -0.30 | -8.85 | 0.03 | N |
| M440V | 10.08 | 1 | 1 | 1.2 | 0.47 | 0.71 | -0.15 | -0.06 | -0.26 | 0.03 | -0.08 | -0.08 | 0.42 | 0.20 | -8.82 | 0.06 | N |
| N493S | 9.67 | 4 | 0 | 0.82 | 0.42 | 0.70 | -0.24 | -0.04 | -0.26 | 0.01 | 0.07 | -0.03 | 0.08 | 0.10 | -8.76 | 0.12 | N |
| A590G | 18.05 | 4 | 1 | 0.82 | -0.13 | 0.92 | 0.17 | 0.07 | -0.21 | 0.03 | 0.00 | 0.00 | 0.24 | -0.25 | -8.83 | 0.05 | N |
| I789V | 36.13 | 1 | 1 | 1.04 | -0.31 | 0.70 | 0.20 | 0.04 | -0.15 | 0.11 | -0.03 | 0.04 | 0.26 | 0.18 | -8.84 | 0.04 | N |
| E795V | 34.86 | 2 | 0 | 0.62 | 0.57 | 0.74 | -0.67 | -0.05 | -0.22 | 0.12 | 0.09 | 0.03 | -0.05 | 0.07 | -8.86 | 0.02 | N |
| D1012G | 46.26 | 1 | 1 | 0.48 | 0.52 | 0.67 | -0.37 | -0.01 | -0.13 | 0.10 | -0.07 | -0.05 | 0.11 | -0.28 | -8.86 | 0.02 | N |
| A1143T | 26.45 | 1 | 0 | -0.35 | -0.04 | -0.23 | 0.09 | 0.02 | -0.05 | 0.01 | 0.01 | -0.03 | 0.10 | -0.22 | -8.85 | 0.03 | N |
| Test set | | | | | | | | | | | | | | | | | |
| M434I | 10.08 | 1 | 1 | 1.16 | 0.48 | 0.73 | -0.22 | -0.07 | -0.26 | 0.02 | -0.09 | -0.06 | 0.41 | 0.21 | -8.86 | 0.02 | P |
| H445Q | 10.17 | 3 | 1 | 1.4 | 0.55 | 0.74 | -0.12 | -0.05 | -0.26 | 0.04 | -0.01 | -0.03 | 0.35 | 0.18 | -8.78 | 0.1 | P |
| T482P | 12.56 | 1 | 1 | 0.7 | 0.53 | 0.68 | -0.02 | -0.03 | -0.26 | 0.02 | -0.11 | -0.10 | 0.11 | -0.13 | -8.92 | -0.04 | P |
| T361I | 35.17 | 1 | 0 | -0.24 | -0.05 | 0.57 | -0.69 | 0.04 | -0.08 | 0.01 | -0.03 | -0.03 | 0.10 | -0.07 | -8.81 | 0.07 | P |
| S428R | 11.65 | 1 | 0 | 0.62 | 0.55 | 0.76 | -0.01 | 0.00 | -0.29 | 0.05 | -0.06 | -0.17 | 0.02 | -0.24 | -8.85 | 0.03 | P |
| L430Q | 9.16 | 4 | 1 | 2.06 | 0.50 | 0.72 | 0.88 | -0.07 | -0.33 | -0.07 | 0.03 | -0.11 | 0.39 | 0.14 | -8.18 | 0.7 | P |
| S431Y | 10.1 | 2 | 1 | 0.63 | 0.63 | 0.96 | -0.72 | 0.04 | -0.16 | 0.13 | -0.06 | -0.07 | 0.07 | -0.19 | -8.91 | -0.03 | P |
| Q432P | 7.4 | 2 | 0 | 0.65 | 0.55 | 0.66 | -0.24 | -0.01 | -0.30 | 0.04 | -0.02 | -0.08 | -0.06 | 0.11 | -6.77 | 2.11 | P |
| G442V | 13.24 | 3 | 1 | 1.09 | 0.64 | 0.87 | -0.53 | 0.05 | -0.14 | 0.10 | 0.01 | -0.02 | 0.21 | -0.10 | -8.77 | 0.11 | P |
| T444R | 13.07 | 3 | 1 | 1.06 | 0.33 | 0.87 | 0.03 | -0.02 | -0.24 | -0.02 | -0.03 | -0.02 | 0.27 | -0.10 | -7.19 | 1.69 | P |
| H445L | 10.17 | 3 | 1 | 0.97 | 0.53 | 0.87 | -0.84 | -0.01 | -0.16 | 0.13 | 0.06 | -0.03 | 0.30 | 0.13 | -7.75 | 1.13 | P |
| R448H | 11.16 | 2 | 0 | 1.12 | 0.54 | 0.66 | 0.01 | 0.01 | -0.37 | 0.05 | 0.02 | -0.02 | -0.09 | 0.31 | -8.82 | 0.06 | P |
| S450Q | 10.16 | 2 | 1 | 1.24 | 0.56 | 0.81 | 0.11 | 0.05 | -0.26 | -0.01 | 0.04 | 0.02 | 0.19 | -0.26 | -7.2 | 1.68 | P |
| G453A | 11.05 | 1 | 1 | 1.07 | 0.53 | 0.93 | -0.16 | 0.07 | -0.25 | 0.01 | 0.09 | 0.01 | 0.11 | -0.25 | -8.91 | -0.03 | P |
| G453V | 11.05 | 1 | 1 | 1.25 | 0.63 | 1.00 | -0.47 | 0.06 | -0.12 | 0.08 | 0.07 | 0.01 | 0.05 | -0.06 | -8.88 | 0 | P |
| I491L | 10.24 | 2 | 1 | 0.57 | 0.29 | 0.40 | -0.10 | -0.04 | -0.18 | -0.01 | -0.02 | 0.00 | 0.15 | 0.08 | -8.91 | -0.03 | P |
| I491N | 10.24 | 2 | 1 | 1.85 | 0.72 | 0.73 | 0.89 | -0.04 | -0.33 | 0.00 | -0.03 | -0.20 | -0.02 | 0.14 | -9.33 | -0.45 | P |
| I491S | 10.24 | 2 | 1 | 1.55 | 0.49 | 0.66 | 0.87 | -0.03 | -0.35 | -0.02 | -0.02 | -0.21 | 0.01 | 0.15 | -8.55 | 0.33 | P |
| D545E | 49.63 | 1 | 0 | 0.05 | -0.44 | 0.70 | 0.03 | -0.01 | -0.11 | -0.02 | 0.04 | 0.03 | -0.15 | -0.01 | -8.87 | 0.01 | P |
| D545N | 49.63 | 1 | 0 | -0.2 | -0.44 | 0.74 | -0.21 | -0.05 | -0.07 | -0.03 | 0.02 | 0.02 | -0.17 | -0.02 | -8.85 | 0.03 | P |
| R662H | 38.75 | 2 | 0 | 0.18 | -0.03 | 0.21 | 0.04 | -0.07 | -0.09 | -0.01 | -0.03 | 0.00 | -0.09 | 0.24 | -8.89 | -0.01 | P |
| H674R | 13.68 | 4 | 0 | -0.2 | 0.48 | -0.70 | -0.05 | -0.05 | -0.10 | -0.01 | 0.03 | 0.02 | 0.05 | 0.13 | -8.86 | 0.02 | P |
| H674Y | 13.68 | 4 | 0 | 0.09 | 0.57 | 0.25 | -0.75 | 0.03 | -0.15 | 0.07 | -0.02 | 0.03 | -0.02 | 0.08 | -8.49 | 0.39 | P |
| H723Y | 34.87 | 4 | 0 | -0.87 | -0.35 | -0.56 | -0.71 | -0.08 | 0.03 | -0.12 | 0.00 | 0.02 | 0.59 | 0.31 | -8.84 | 0.04 | P |
| H835P | 26.63 | 4 | 0 | 0.47 | 0.41 | 0.65 | -0.10 | 0.01 | -0.22 | 0.11 | -0.02 | 0.02 | -0.47 | 0.08 | -8.91 | -0.03 | P |
| D53N | 24.19 | 4 | 0 | -0.53 | -0.38 | 0.05 | -0.16 | -0.03 | -0.05 | -0.09 | 0.00 | 0.03 | 0.07 | 0.04 | -8.86 | 0.02 | N |
| V109I | 29.97 | 1 | 0 | 0.31 | 0.30 | 0.34 | -0.24 | -0.01 | -0.15 | -0.01 | -0.03 | 0.04 | 0.04 | 0.02 | -8.8 | 0.08 | N |
| L314V | 39.74 | 3 | 1 | 1.29 | 0.26 | 0.66 | -0.06 | 0.00 | -0.19 | -0.01 | -0.08 | 0.01 | 0.53 | 0.16 | -8.86 | 0.02 | N |
| A334D | 43.5 | 3 | 1 | 1.28 | 0.47 | 0.78 | 0.19 | 0.05 | -0.26 | -0.04 | -0.06 | 0.02 | 0.35 | -0.23 | -8.87 | 0.01 | N |
| D362H | 31.75 | 1 | 0 | 0.68 | 0.54 | 0.71 | -0.30 | -0.03 | -0.16 | 0.09 | -0.10 | 0.02 | 0.14 | -0.22 | -8.81 | 0.07 | N |
| P454L | 14.57 | 4 | 0 | 0.74 | 0.56 | 0.81 | -0.57 | 0.03 | -0.15 | 0.16 | 0.05 | 0.01 | -0.12 | -0.03 | -8.99 | -0.11 | N |
| E639Q | 34.79 | 1 | 0 | -0.12 | -0.07 | 0.53 | -0.14 | -0.14 | -0.12 | -0.05 | -0.01 | 0.00 | -0.25 | 0.11 | -8.86 | 0.02 | N |
| R661Q | 42.52 | 2 | 0 | 0.16 | 0.32 | 0.02 | 0.06 | -0.04 | -0.12 | 0.05 | -0.01 | -0.02 | -0.26 | 0.17 | -8.89 | -0.01 | N |
| E825G | 45.79 | 1 | 0 | 0.55 | 0.59 | 0.65 | -0.21 | -0.02 | -0.24 | 0.10 | -0.03 | 0.01 | -0.38 | 0.08 | -8.84 | 0.04 | N |
| A998V | 46.93 | 4 | 0 | 0.33 | 0.58 | 0.38 | -0.43 | 0.02 | -0.10 | 0.06 | -0.05 | 0.00 | 0.07 | -0.20 | -8.78 | 0.1 | N |
| V1117L | 33.98 | 3 | 1 | 0.8 | 0.44 | 0.62 | -0.24 | -0.02 | -0.23 | 0.00 | 0.09 | 0.01 | 0.11 | 0.02 | -8.86 | 0.02 | N |
| D109E | 37.77 | 2 | 0 | 0.06 | -0.47 | 0.84 | 0.01 | -0.01 | -0.12 | -0.01 | -0.01 | -0.01 | -0.13 | -0.02 | -8.93 | -0.05 | N |
| L1128Q | 30.95 | 3 | 0 | 1.5 | 0.46 | 0.62 | 0.86 | -0.04 | -0.36 | -0.01 | -0.01 | 0.01 | -0.17 | 0.14 | -8.9 | -0.02 | N |

*The numbers of 1, 2, 3 and 4 in the column of “SS” mean the secondary structure of the mutated amino acid being coil, sheet, helix and turn, respectively.

The numbers of 0 and 1 in the column of “Location” denote the location of the mutated amino acid in the protein being surface or core, respectively.

DE: docking energy (kacl•mol^-1^)

Table S4. Confusion matrix of the five developed classifiers on the training and test data, respectively.

| Dataset | Type/Prediction | DT | | kNN | | NB | | PNN | | SVM | | MC | | |
| --- | --- | --- | --- | --- | --- | --- | --- | --- | --- | --- | --- | --- | --- | --- |
|  |  | P | N | P | N | P | N | P | N | P | N | P | N |  |
| Training | P | 75 | 23 | 80 | 18 | 70 | 28 | 74 | 24 | 77 | 21 | 77 | 21 |  |
|  | N | 21 | 29 | 18 | 32 | 10 | 40 | 13 | 37 | 15 | 35 | 11 | 39 |  |
| Test | P | 19 | 6 | 20 | 5 | 18 | 7 | 20 | 5 | 18 | 7 | 20 | 5 |  |
|  | N | 2 | 11 | 5 | 8 | 1 | 12 | 2 | 11 | 2 | 11 | 1 | 12 |  |

NB: Naïve Bayes; DT: decision tree; kNN: k nearest neighbors; PNN: probabilistic neural network; SVM: support vector machine; MC: majority consensus

Table S5. The predictive results for the classifiers on the training and test sets of *Mycobacterium tuberculosis* RpoB mutants. P: positive, N: negative. P_N_ and P_P_ denote the probability values of being negative or positive mutation, respectively. NB: Naïve Bayes; DT: decision tree; kNN: k nearest neighbors; PNN: probabilistic neural network; SVM: support vector machine; MC: majority consensus

| Mutation | Real type | DT | | | kNN | | | NB | | | PNN | | | SVM | | | MC | | |
| --- | --- | --- | --- | --- | --- | --- | --- | --- | --- | --- | --- | --- | --- | --- | --- | --- | --- | --- | --- |
|  |  | P_N_ | P_P_ | Prediction | P_N_ | P_P_ | Prediction | P_N_ | P_P_ | Prediction | P_N_ | P_P_ | Prediction | P_N_ | P_P_ | Prediction | P_N_ | P_P_ | Prediction |
| Training set | | | | | | | | | | | | | | | | | | | |
| Q172L | P | 0.02 | 0.98 | P | 0.00 | 1.00 | P | 0.03 | 0.97 | P | 0.00 | 1.00 | P | 0.27 | 0.73 | P | 0.00 | 1.00 | P |
| V359A | P | 1.00 | 0.00 | N | 0.34 | 0.66 | P | 0.97 | 0.03 | N | 0.58 | 0.42 | N | 0.70 | 0.30 | N | 0.80 | 0.20 | N |
| D435N | P | 0.00 | 1.00 | P | 0.00 | 1.00 | P | 0.00 | 1.00 | P | 0.01 | 0.99 | P | 0.24 | 0.76 | P | 0.00 | 1.00 | P |
| H445N | P | 0.00 | 1.00 | P | 0.00 | 1.00 | P | 0.00 | 1.00 | P | 0.01 | 0.99 | P | 0.20 | 0.80 | P | 0.00 | 1.00 | P |
| H445Y | P | 0.02 | 0.98 | P | 0.00 | 1.00 | P | 0.00 | 1.00 | P | 0.00 | 1.00 | P | 0.19 | 0.81 | P | 0.00 | 1.00 | P |
| S450L | P | 0.00 | 1.00 | P | 0.00 | 1.00 | P | 0.00 | 1.00 | P | 0.00 | 1.00 | P | 0.16 | 0.84 | P | 0.00 | 1.00 | P |
| S450F | P | 0.02 | 0.98 | P | 0.00 | 1.00 | P | 0.00 | 1.00 | P | 0.00 | 1.00 | P | 0.22 | 0.78 | P | 0.00 | 1.00 | P |
| S493F | P | 0.00 | 1.00 | P | 0.00 | 1.00 | P | 0.00 | 1.00 | P | 0.00 | 1.00 | P | 0.19 | 0.81 | P | 0.00 | 1.00 | P |
| S493Y | P | 0.00 | 1.00 | P | 0.00 | 1.00 | P | 0.56 | 0.44 | N | 0.00 | 1.00 | P | 0.51 | 0.49 | N | 0.40 | 0.60 | P |
| T676P | P | 1.00 | 0.00 | N | 0.32 | 0.68 | P | 0.69 | 0.31 | N | 0.07 | 0.93 | P | 0.32 | 0.68 | P | 0.40 | 0.60 | P |
| G981D | P | 0.90 | 0.10 | N | 0.29 | 0.71 | P | 1.00 | 0.00 | N | 0.81 | 0.19 | N | 0.80 | 0.20 | N | 0.80 | 0.20 | N |
| L42F | P | 0.96 | 0.04 | N | 1.00 | 0.00 | N | 0.99 | 0.01 | N | 0.94 | 0.06 | N | 0.63 | 0.37 | N | 1.00 | 0.00 | N |
| L47R | P | 0.05 | 0.95 | P | 0.32 | 0.68 | P | 0.03 | 0.97 | P | 0.05 | 0.95 | P | 0.15 | 0.85 | P | 0.00 | 1.00 | P |
| E82G | P | 0.25 | 0.75 | P | 1.00 | 0.00 | N | 0.64 | 0.36 | N | 0.95 | 0.05 | N | 0.46 | 0.54 | P | 0.60 | 0.40 | N |
| I90L | P | 0.00 | 1.00 | P | 0.30 | 0.70 | P | 0.38 | 0.62 | P | 0.60 | 0.40 | N | 0.62 | 0.38 | N | 0.40 | 0.60 | P |
| V170F | P | 0.02 | 0.98 | P | 0.00 | 1.00 | P | 0.00 | 1.00 | P | 0.00 | 1.00 | P | 0.18 | 0.82 | P | 0.00 | 1.00 | P |
| V170G | P | 0.67 | 0.33 | N | 0.00 | 1.00 | P | 0.00 | 1.00 | P | 0.00 | 1.00 | P | 0.07 | 0.93 | P | 0.20 | 0.80 | P |
| Q172H | P | 0.04 | 0.96 | P | 0.00 | 1.00 | P | 0.00 | 1.00 | P | 0.00 | 1.00 | P | 0.17 | 0.83 | P | 0.00 | 1.00 | P |
| Q172K | P | 0.02 | 0.98 | P | 0.00 | 1.00 | P | 0.00 | 1.00 | P | 0.00 | 1.00 | P | 0.16 | 0.84 | P | 0.00 | 1.00 | P |
| Q172P | P | 0.02 | 0.98 | P | 0.00 | 1.00 | P | 0.00 | 1.00 | P | 0.00 | 1.00 | P | 0.18 | 0.82 | P | 0.00 | 1.00 | P |
| Q172R | P | 0.00 | 1.00 | P | 0.00 | 1.00 | P | 0.00 | 1.00 | P | 0.00 | 1.00 | P | 0.16 | 0.84 | P | 0.00 | 1.00 | P |
| D265G | P | 0.00 | 1.00 | P | 0.32 | 0.68 | P | 1.00 | 0.00 | N | 1.00 | 0.00 | N | 0.87 | 0.13 | N | 0.60 | 0.40 | N |
| P280S | P | 0.00 | 1.00 | P | 1.00 | 0.00 | N | 1.00 | 0.00 | N | 0.67 | 0.33 | N | 0.83 | 0.17 | N | 0.80 | 0.20 | N |
| L378R | P | 0.00 | 1.00 | P | 0.37 | 0.63 | P | 0.04 | 0.96 | P | 0.37 | 0.63 | P | 0.17 | 0.83 | P | 0.00 | 1.00 | P |
| T399A | P | 0.96 | 0.04 | N | 0.68 | 0.32 | N | 1.00 | 0.00 | N | 0.74 | 0.26 | N | 0.82 | 0.18 | N | 1.00 | 0.00 | N |
| T400I | P | 1.00 | 0.00 | N | 0.70 | 0.30 | N | 1.00 | 0.00 | N | 0.71 | 0.29 | N | 0.95 | 0.05 | N | 1.00 | 0.00 | N |
| F424L | P | 0.00 | 1.00 | P | 0.52 | 0.48 | N | 0.16 | 0.84 | P | 0.18 | 0.82 | P | 0.22 | 0.78 | P | 0.20 | 0.80 | P |
| G426D | P | 0.00 | 1.00 | P | 0.32 | 0.68 | P | 0.57 | 0.43 | N | 0.02 | 0.98 | P | 0.35 | 0.65 | P | 0.20 | 0.80 | P |
| T427P | P | 0.00 | 1.00 | P | 0.00 | 1.00 | P | 0.56 | 0.44 | N | 0.01 | 0.99 | P | 0.34 | 0.66 | P | 0.20 | 0.80 | P |
| L430P | P | 0.00 | 1.00 | P | 0.00 | 1.00 | P | 0.00 | 1.00 | P | 0.03 | 0.97 | P | 0.08 | 0.92 | P | 0.00 | 1.00 | P |
| L430R | P | 0.67 | 0.33 | N | 0.00 | 1.00 | P | 0.00 | 1.00 | P | 0.04 | 0.96 | P | 0.16 | 0.84 | P | 0.20 | 0.80 | P |
| S431A | P | 0.02 | 0.98 | P | 0.00 | 1.00 | P | 0.01 | 0.99 | P | 0.00 | 1.00 | P | 0.24 | 0.76 | P | 0.00 | 1.00 | P |
| S431F | P | 0.00 | 1.00 | P | 0.00 | 1.00 | P | 0.00 | 1.00 | P | 0.01 | 0.99 | P | 0.27 | 0.73 | P | 0.00 | 1.00 | P |
| S431P | P | 0.02 | 0.98 | P | 0.00 | 1.00 | P | 0.00 | 1.00 | P | 0.02 | 0.98 | P | 0.24 | 0.76 | P | 0.00 | 1.00 | P |
| S431R | P | 0.00 | 1.00 | P | 0.00 | 1.00 | P | 0.00 | 1.00 | P | 0.01 | 0.99 | P | 0.26 | 0.74 | P | 0.00 | 1.00 | P |
| S431T | P | 0.00 | 1.00 | P | 0.00 | 1.00 | P | 0.09 | 0.91 | P | 0.01 | 0.99 | P | 0.32 | 0.68 | P | 0.00 | 1.00 | P |
| Q432K | P | 0.04 | 0.96 | P | 0.00 | 1.00 | P | 0.00 | 1.00 | P | 0.00 | 1.00 | P | 0.27 | 0.73 | P | 0.00 | 1.00 | P |
| Q432L | P | 0.00 | 1.00 | P | 0.00 | 1.00 | P | 0.00 | 1.00 | P | 0.00 | 1.00 | P | 0.22 | 0.78 | P | 0.00 | 1.00 | P |
| Q432R | P | 0.02 | 0.98 | P | 0.00 | 1.00 | P | 0.00 | 1.00 | P | 0.00 | 1.00 | P | 0.12 | 0.88 | P | 0.00 | 1.00 | P |
| D435A | P | 0.02 | 0.98 | P | 0.29 | 0.71 | P | 0.00 | 1.00 | P | 0.02 | 0.98 | P | 0.27 | 0.73 | P | 0.00 | 1.00 | P |
| D435F | P | 0.00 | 1.00 | P | 0.00 | 1.00 | P | 0.00 | 1.00 | P | 0.00 | 1.00 | P | 0.17 | 0.83 | P | 0.00 | 1.00 | P |
| D435G | P | 0.00 | 1.00 | P | 0.00 | 1.00 | P | 0.00 | 1.00 | P | 0.00 | 1.00 | P | 0.20 | 0.80 | P | 0.00 | 1.00 | P |
| D435V | P | 0.00 | 1.00 | P | 0.00 | 1.00 | P | 0.00 | 1.00 | P | 0.00 | 1.00 | P | 0.23 | 0.77 | P | 0.00 | 1.00 | P |
| D435Y | P | 0.00 | 1.00 | P | 0.00 | 1.00 | P | 0.00 | 1.00 | P | 0.00 | 1.00 | P | 0.23 | 0.77 | P | 0.00 | 1.00 | P |
| N437D | P | 0.00 | 1.00 | P | 0.00 | 1.00 | P | 0.07 | 0.93 | P | 0.00 | 1.00 | P | 0.30 | 0.70 | P | 0.00 | 1.00 | P |
| S441F | P | 0.00 | 1.00 | P | 0.00 | 1.00 | P | 0.00 | 1.00 | P | 0.00 | 1.00 | P | 0.22 | 0.78 | P | 0.00 | 1.00 | P |
| S441L | P | 0.00 | 1.00 | P | 0.00 | 1.00 | P | 0.00 | 1.00 | P | 0.00 | 1.00 | P | 0.17 | 0.83 | P | 0.00 | 1.00 | P |
| S441Q | P | 1.00 | 0.00 | N | 0.00 | 1.00 | P | 0.00 | 1.00 | P | 0.00 | 1.00 | P | 0.20 | 0.80 | P | 0.20 | 0.80 | P |
| S441Y | P | 0.00 | 1.00 | P | 0.00 | 1.00 | P | 0.00 | 1.00 | P | 0.00 | 1.00 | P | 0.19 | 0.81 | P | 0.00 | 1.00 | P |
| H445C | P | 0.00 | 1.00 | P | 0.00 | 1.00 | P | 0.00 | 1.00 | P | 0.02 | 0.98 | P | 0.13 | 0.87 | P | 0.00 | 1.00 | P |
| H445D | P | 0.00 | 1.00 | P | 0.00 | 1.00 | P | 0.00 | 1.00 | P | 0.02 | 0.98 | P | 0.15 | 0.85 | P | 0.00 | 1.00 | P |
| H445F | P | 0.00 | 1.00 | P | 0.00 | 1.00 | P | 0.00 | 1.00 | P | 0.01 | 0.99 | P | 0.18 | 0.82 | P | 0.00 | 1.00 | P |
| H445G | P | 0.00 | 1.00 | P | 0.00 | 1.00 | P | 0.00 | 1.00 | P | 0.00 | 1.00 | P | 0.14 | 0.86 | P | 0.00 | 1.00 | P |
| H445P | P | 0.00 | 1.00 | P | 0.00 | 1.00 | P | 0.00 | 1.00 | P | 0.01 | 0.99 | P | 0.12 | 0.88 | P | 0.00 | 1.00 | P |
| H445R | P | 0.06 | 0.94 | P | 0.00 | 1.00 | P | 0.00 | 1.00 | P | 0.00 | 1.00 | P | 0.14 | 0.86 | P | 0.00 | 1.00 | P |
| R448C | P | 0.02 | 0.98 | P | 0.00 | 1.00 | P | 0.00 | 1.00 | P | 0.00 | 1.00 | P | 0.26 | 0.74 | P | 0.00 | 1.00 | P |
| R448L | P | 0.02 | 0.98 | P | 0.00 | 1.00 | P | 0.00 | 1.00 | P | 0.00 | 1.00 | P | 0.29 | 0.71 | P | 0.00 | 1.00 | P |
| R448S | P | 0.00 | 1.00 | P | 0.00 | 1.00 | P | 0.00 | 1.00 | P | 0.00 | 1.00 | P | 0.24 | 0.76 | P | 0.00 | 1.00 | P |
| S450P | P | 0.00 | 1.00 | P | 0.00 | 1.00 | P | 0.00 | 1.00 | P | 0.01 | 0.99 | P | 0.21 | 0.79 | P | 0.00 | 1.00 | P |
| S450W | P | 0.02 | 0.98 | P | 0.00 | 1.00 | P | 0.00 | 1.00 | P | 0.00 | 1.00 | P | 0.12 | 0.88 | P | 0.00 | 1.00 | P |
| S450Y | P | 0.00 | 1.00 | P | 0.00 | 1.00 | P | 0.00 | 1.00 | P | 0.00 | 1.00 | P | 0.19 | 0.81 | P | 0.00 | 1.00 | P |
| A451E | P | 0.00 | 1.00 | P | 0.00 | 1.00 | P | 0.03 | 0.97 | P | 0.00 | 1.00 | P | 0.16 | 0.84 | P | 0.00 | 1.00 | P |
| A451V | P | 0.00 | 1.00 | P | 0.00 | 1.00 | P | 0.16 | 0.84 | P | 0.00 | 1.00 | P | 0.18 | 0.82 | P | 0.00 | 1.00 | P |
| L452H | P | 0.00 | 1.00 | P | 0.00 | 1.00 | P | 0.00 | 1.00 | P | 0.00 | 1.00 | P | 0.10 | 0.90 | P | 0.00 | 1.00 | P |
| L452P | P | 0.00 | 1.00 | P | 0.32 | 0.68 | P | 0.00 | 1.00 | P | 0.01 | 0.99 | P | 0.12 | 0.88 | P | 0.00 | 1.00 | P |
| L452R | P | 0.00 | 1.00 | P | 0.31 | 0.69 | P | 0.00 | 1.00 | P | 0.02 | 0.98 | P | 0.13 | 0.87 | P | 0.00 | 1.00 | P |
| G453C | P | 0.00 | 1.00 | P | 0.18 | 0.82 | P | 0.00 | 1.00 | P | 0.03 | 0.97 | P | 0.18 | 0.82 | P | 0.00 | 1.00 | P |
| G453D | P | 0.00 | 1.00 | P | 0.16 | 0.84 | P | 0.10 | 0.90 | P | 0.02 | 0.98 | P | 0.20 | 0.80 | P | 0.00 | 1.00 | P |
| G453S | P | 0.00 | 1.00 | P | 0.14 | 0.86 | P | 0.10 | 0.90 | P | 0.02 | 0.98 | P | 0.21 | 0.79 | P | 0.00 | 1.00 | P |
| G456S | P | 0.13 | 0.88 | P | 0.00 | 1.00 | P | 0.75 | 0.25 | N | 0.00 | 1.00 | P | 0.39 | 0.61 | P | 0.20 | 0.80 | P |
| P483L | P | 0.02 | 0.98 | P | 0.00 | 1.00 | P | 0.00 | 1.00 | P | 0.00 | 1.00 | P | 0.26 | 0.74 | P | 0.00 | 1.00 | P |
| P483R | P | 0.02 | 0.98 | P | 0.00 | 1.00 | P | 0.00 | 1.00 | P | 0.01 | 0.99 | P | 0.16 | 0.84 | P | 0.00 | 1.00 | P |
| I488V | P | 1.00 | 0.00 | N | 0.28 | 0.72 | P | 0.99 | 0.01 | N | 0.07 | 0.93 | P | 0.38 | 0.62 | P | 0.40 | 0.60 | P |
| G489C | P | 0.00 | 1.00 | P | 0.35 | 0.65 | P | 0.01 | 0.99 | P | 0.01 | 0.99 | P | 0.23 | 0.77 | P | 0.00 | 1.00 | P |
| I491F | P | 0.00 | 1.00 | P | 0.00 | 1.00 | P | 0.00 | 1.00 | P | 0.00 | 1.00 | P | 0.16 | 0.84 | P | 0.00 | 1.00 | P |
| I491M | P | 0.05 | 0.95 | P | 0.00 | 1.00 | P | 0.00 | 1.00 | P | 0.01 | 0.99 | P | 0.22 | 0.78 | P | 0.00 | 1.00 | P |
| I491T | P | 0.00 | 1.00 | P | 0.00 | 1.00 | P | 0.00 | 1.00 | P | 0.01 | 0.99 | P | 0.20 | 0.80 | P | 0.00 | 1.00 | P |
| I491V | P | 0.05 | 0.95 | P | 0.00 | 1.00 | P | 0.58 | 0.42 | N | 0.02 | 0.98 | P | 0.23 | 0.77 | P | 0.20 | 0.80 | P |
| V496L | P | 0.00 | 1.00 | P | 0.44 | 0.56 | P | 0.85 | 0.15 | N | 0.34 | 0.66 | P | 0.49 | 0.51 | P | 0.20 | 0.80 | P |
| V496M | P | 0.96 | 0.04 | N | 0.41 | 0.59 | P | 0.95 | 0.05 | N | 0.22 | 0.78 | P | 0.37 | 0.63 | P | 0.40 | 0.60 | P |
| F503S | P | 1.00 | 0.00 | N | 1.00 | 0.00 | N | 0.14 | 0.86 | P | 0.99 | 0.01 | N | 0.47 | 0.53 | P | 0.60 | 0.40 | N |
| P551S | P | 0.91 | 0.09 | N | 0.72 | 0.28 | N | 1.00 | 0.00 | N | 0.83 | 0.17 | N | 0.91 | 0.09 | N | 1.00 | 0.00 | N |
| D571A | P | 0.00 | 1.00 | P | 0.80 | 0.20 | N | 0.98 | 0.02 | N | 0.88 | 0.12 | N | 0.65 | 0.35 | N | 0.80 | 0.20 | N |
| D574E | P | 0.96 | 0.04 | N | 1.00 | 0.00 | N | 0.30 | 0.70 | P | 0.88 | 0.12 | N | 0.62 | 0.38 | N | 0.80 | 0.20 | N |
| R607H | P | 0.00 | 1.00 | P | 0.00 | 1.00 | P | 0.00 | 1.00 | P | 0.00 | 1.00 | P | 0.11 | 0.89 | P | 0.00 | 1.00 | P |
| N658D | P | 0.96 | 0.04 | N | 0.68 | 0.32 | N | 1.00 | 0.00 | N | 0.89 | 0.11 | N | 0.93 | 0.07 | N | 1.00 | 0.00 | N |
| A670D | P | 0.90 | 0.10 | N | 0.39 | 0.61 | P | 1.00 | 0.00 | N | 0.67 | 0.33 | N | 0.49 | 0.51 | P | 0.60 | 0.40 | N |
| C681W | P | 0.93 | 0.07 | N | 1.00 | 0.00 | N | 1.00 | 0.00 | N | 0.90 | 0.10 | N | 0.70 | 0.30 | N | 1.00 | 0.00 | N |
| M707T | P | 0.00 | 1.00 | P | 0.45 | 0.55 | P | 0.29 | 0.71 | P | 0.58 | 0.42 | N | 0.33 | 0.67 | P | 0.20 | 0.80 | P |
| H723D | P | 0.96 | 0.04 | N | 1.00 | 0.00 | N | 0.95 | 0.05 | N | 0.98 | 0.02 | N | 0.55 | 0.45 | N | 1.00 | 0.00 | N |
| L731P | P | 0.75 | 0.25 | N | 0.34 | 0.66 | P | 0.00 | 1.00 | P | 0.39 | 0.61 | P | 0.27 | 0.73 | P | 0.20 | 0.80 | P |
| L735Q | P | 0.13 | 0.88 | P | 0.39 | 0.61 | P | 0.02 | 0.98 | P | 0.51 | 0.49 | N | 0.28 | 0.72 | P | 0.20 | 0.80 | P |
| H745Y | P | 1.00 | 0.00 | N | 0.36 | 0.64 | P | 0.24 | 0.76 | P | 0.15 | 0.85 | P | 0.57 | 0.43 | N | 0.40 | 0.60 | P |
| E761D | P | 0.33 | 0.67 | P | 1.00 | 0.00 | N | 0.97 | 0.03 | N | 0.99 | 0.01 | N | 0.69 | 0.31 | N | 0.80 | 0.20 | N |
| R827C | P | 0.93 | 0.07 | N | 0.68 | 0.32 | N | 0.99 | 0.01 | N | 0.81 | 0.19 | N | 0.68 | 0.32 | N | 1.00 | 0.00 | N |
| H835R | P | 0.13 | 0.88 | P | 1.00 | 0.00 | N | 1.00 | 0.00 | N | 0.99 | 0.01 | N | 0.60 | 0.40 | N | 0.80 | 0.20 | N |
| I925V | P | 0.96 | 0.04 | N | 1.00 | 0.00 | N | 1.00 | 0.00 | N | 0.90 | 0.10 | N | 0.88 | 0.12 | N | 1.00 | 0.00 | N |
| E978D | P | 0.96 | 0.04 | N | 1.00 | 0.00 | N | 1.00 | 0.00 | N | 0.96 | 0.04 | N | 0.85 | 0.15 | N | 1.00 | 0.00 | N |
| N24D | N | 0.96 | 0.04 | N | 0.67 | 0.33 | N | 1.00 | 0.00 | N | 0.94 | 0.06 | N | 0.58 | 0.42 | N | 1.00 | 0.00 | N |
| G28R | N | 0.96 | 0.04 | N | 0.62 | 0.38 | N | 0.96 | 0.04 | N | 0.84 | 0.16 | N | 0.63 | 0.37 | N | 1.00 | 0.00 | N |
| P30S | N | 0.00 | 1.00 | P | 1.00 | 0.00 | N | 1.00 | 0.00 | N | 0.96 | 0.04 | N | 0.71 | 0.29 | N | 0.80 | 0.20 | N |
| E66K | N | 1.00 | 0.00 | N | 0.68 | 0.32 | N | 1.00 | 0.00 | N | 0.80 | 0.20 | N | 0.72 | 0.28 | N | 1.00 | 0.00 | N |
| A69P | N | 0.96 | 0.04 | N | 1.00 | 0.00 | N | 1.00 | 0.00 | N | 1.00 | 0.00 | N | 0.68 | 0.32 | N | 1.00 | 0.00 | N |
| V77M | N | 0.96 | 0.04 | N | 0.58 | 0.42 | N | 1.00 | 0.00 | N | 0.61 | 0.39 | N | 0.72 | 0.28 | N | 1.00 | 0.00 | N |
| L80V | N | 0.20 | 0.80 | P | 0.71 | 0.29 | N | 0.88 | 0.12 | N | 0.97 | 0.03 | N | 0.40 | 0.60 | P | 0.60 | 0.40 | N |
| P89L | N | 1.00 | 0.00 | N | 0.26 | 0.74 | P | 0.92 | 0.08 | N | 0.49 | 0.51 | P | 0.67 | 0.33 | N | 0.60 | 0.40 | N |
| V113I | N | 0.17 | 0.83 | P | 0.32 | 0.68 | P | 1.00 | 0.00 | N | 0.77 | 0.23 | N | 0.60 | 0.40 | N | 0.60 | 0.40 | N |
| M121I | N | 0.96 | 0.04 | N | 0.29 | 0.71 | P | 1.00 | 0.00 | N | 0.53 | 0.47 | N | 0.55 | 0.45 | N | 0.80 | 0.20 | N |
| E132D | N | 0.13 | 0.88 | P | 1.00 | 0.00 | N | 1.00 | 0.00 | N | 0.87 | 0.13 | N | 0.76 | 0.24 | N | 0.80 | 0.20 | N |
| M153T | N | 0.04 | 0.96 | P | 0.00 | 1.00 | P | 0.12 | 0.88 | P | 0.26 | 0.74 | P | 0.30 | 0.70 | P | 0.00 | 1.00 | P |
| V179A | N | 0.13 | 0.88 | P | 0.68 | 0.32 | N | 0.33 | 0.67 | P | 0.63 | 0.37 | N | 0.44 | 0.56 | P | 0.40 | 0.60 | P |
| S195R | N | 0.90 | 0.10 | N | 0.69 | 0.31 | N | 0.92 | 0.08 | N | 0.91 | 0.09 | N | 0.58 | 0.42 | N | 1.00 | 0.00 | N |
| D270E | N | 0.00 | 1.00 | P | 0.65 | 0.35 | N | 1.00 | 0.00 | N | 0.70 | 0.30 | N | 0.87 | 0.13 | N | 0.80 | 0.20 | N |
| L316V | N | 0.00 | 1.00 | P | 0.72 | 0.28 | N | 0.96 | 0.04 | N | 0.91 | 0.09 | N | 0.65 | 0.35 | N | 0.80 | 0.20 | N |
| H343Q | N | 0.83 | 0.17 | N | 0.32 | 0.68 | P | 0.99 | 0.01 | N | 0.50 | 0.50 | N | 0.58 | 0.42 | N | 0.80 | 0.20 | N |
| T350I | N | 1.00 | 0.00 | N | 0.67 | 0.33 | N | 1.00 | 0.00 | N | 0.52 | 0.48 | N | 0.88 | 0.12 | N | 1.00 | 0.00 | N |
| P358L | N | 0.93 | 0.07 | N | 0.73 | 0.27 | N | 1.00 | 0.00 | N | 0.87 | 0.13 | N | 0.83 | 0.17 | N | 1.00 | 0.00 | N |
| S388L | N | 0.00 | 1.00 | P | 0.37 | 0.63 | P | 0.91 | 0.09 | N | 0.76 | 0.24 | N | 0.52 | 0.48 | N | 0.60 | 0.40 | N |
| M390T | N | 0.75 | 0.25 | N | 0.34 | 0.66 | P | 0.34 | 0.66 | P | 0.38 | 0.62 | P | 0.37 | 0.63 | P | 0.20 | 0.80 | P |
| L443F | N | 0.00 | 1.00 | P | 0.00 | 1.00 | P | 0.06 | 0.94 | P | 0.00 | 1.00 | P | 0.17 | 0.83 | P | 0.00 | 1.00 | P |
| R511L | N | 1.00 | 0.00 | N | 0.66 | 0.34 | N | 0.23 | 0.77 | P | 0.88 | 0.12 | N | 0.45 | 0.55 | P | 0.60 | 0.40 | N |
| D515Y | N | 1.00 | 0.00 | N | 1.00 | 0.00 | N | 0.99 | 0.01 | N | 0.89 | 0.11 | N | 0.82 | 0.18 | N | 1.00 | 0.00 | N |
| T526S | N | 0.90 | 0.10 | N | 1.00 | 0.00 | N | 0.97 | 0.03 | N | 1.00 | 0.00 | N | 0.44 | 0.56 | P | 0.80 | 0.20 | N |
| A544V | N | 0.91 | 0.09 | N | 1.00 | 0.00 | N | 1.00 | 0.00 | N | 0.93 | 0.07 | N | 0.85 | 0.15 | N | 1.00 | 0.00 | N |
| D545A | N | 0.67 | 0.33 | N | 0.37 | 0.63 | P | 1.00 | 0.00 | N | 0.50 | 0.50 | P | 0.85 | 0.15 | N | 0.60 | 0.40 | N |
| E563D | N | 1.00 | 0.00 | N | 0.68 | 0.32 | N | 1.00 | 0.00 | N | 0.45 | 0.55 | P | 0.77 | 0.23 | N | 0.80 | 0.20 | N |
| D634G | N | 0.00 | 1.00 | P | 0.00 | 1.00 | P | 0.49 | 0.51 | P | 0.14 | 0.86 | P | 0.43 | 0.57 | P | 0.00 | 1.00 | P |
| E639G | N | 0.00 | 1.00 | P | 0.71 | 0.29 | N | 0.99 | 0.01 | N | 0.95 | 0.05 | N | 0.59 | 0.41 | N | 0.80 | 0.20 | N |
| H674Q | N | 0.00 | 1.00 | P | 0.00 | 1.00 | P | 1.00 | 0.00 | N | 0.00 | 1.00 | P | 0.31 | 0.69 | P | 0.20 | 0.80 | P |
| P682T | N | 0.90 | 0.10 | N | 0.73 | 0.27 | N | 0.96 | 0.04 | N | 0.82 | 0.18 | N | 0.58 | 0.42 | N | 1.00 | 0.00 | N |
| V695L | N | 0.96 | 0.04 | N | 0.40 | 0.60 | P | 0.98 | 0.02 | N | 0.68 | 0.32 | N | 0.56 | 0.44 | N | 0.80 | 0.20 | N |
| P834L | N | 0.96 | 0.04 | N | 0.70 | 0.30 | N | 0.95 | 0.05 | N | 0.83 | 0.17 | N | 0.49 | 0.51 | P | 0.80 | 0.20 | N |
| D851G | N | 0.93 | 0.07 | N | 0.75 | 0.25 | N | 1.00 | 0.00 | N | 0.99 | 0.01 | N | 0.75 | 0.25 | N | 1.00 | 0.00 | N |
| A857T | N | 0.90 | 0.10 | N | 0.00 | 1.00 | P | 1.00 | 0.00 | N | 0.33 | 0.67 | P | 0.60 | 0.40 | N | 0.60 | 0.40 | N |
| G890D | N | 0.00 | 1.00 | P | 0.65 | 0.35 | N | 0.99 | 0.01 | N | 0.79 | 0.21 | N | 0.57 | 0.43 | N | 0.80 | 0.20 | N |
| L893R | N | 0.00 | 1.00 | P | 0.63 | 0.37 | N | 0.02 | 0.98 | P | 0.92 | 0.08 | N | 0.39 | 0.61 | P | 0.40 | 0.60 | P |
| K944E | N | 0.00 | 1.00 | P | 0.33 | 0.67 | P | 1.00 | 0.00 | N | 1.00 | 0.00 | N | 0.91 | 0.09 | N | 0.60 | 0.40 | N |
| S1124A | N | 0.90 | 0.10 | N | 1.00 | 0.00 | N | 1.00 | 0.00 | N | 0.98 | 0.02 | N | 0.78 | 0.22 | N | 1.00 | 0.00 | N |
| V1129A | N | 0.13 | 0.88 | P | 1.00 | 0.00 | N | 0.94 | 0.06 | N | 0.99 | 0.01 | N | 0.61 | 0.39 | N | 0.80 | 0.20 | N |
| P233Q | N | 1.00 | 0.00 | N | 0.60 | 0.40 | N | 1.00 | 0.00 | N | 0.74 | 0.26 | N | 0.75 | 0.25 | N | 1.00 | 0.00 | N |
| G374S | N | 1.00 | 0.00 | N | 0.24 | 0.76 | P | 0.43 | 0.57 | P | 0.32 | 0.68 | P | 0.37 | 0.63 | P | 0.20 | 0.80 | P |
| M440V | N | 0.02 | 0.98 | P | 0.00 | 1.00 | P | 0.01 | 0.99 | P | 0.00 | 1.00 | P | 0.20 | 0.80 | P | 0.00 | 1.00 | P |
| N493S | N | 0.00 | 1.00 | P | 0.00 | 1.00 | P | 0.01 | 0.99 | P | 0.00 | 1.00 | P | 0.18 | 0.82 | P | 0.00 | 1.00 | P |
| A590G | N | 0.00 | 1.00 | P | 0.00 | 1.00 | P | 0.81 | 0.19 | N | 0.00 | 1.00 | P | 0.30 | 0.70 | P | 0.20 | 0.80 | P |
| I789V | N | 1.00 | 0.00 | N | 0.61 | 0.39 | N | 0.98 | 0.02 | N | 0.93 | 0.07 | N | 0.53 | 0.47 | N | 1.00 | 0.00 | N |
| E795V | N | 1.00 | 0.00 | N | 0.79 | 0.21 | N | 0.96 | 0.04 | N | 0.88 | 0.12 | N | 0.53 | 0.47 | N | 1.00 | 0.00 | N |
| D1012G | N | 1.00 | 0.00 | N | 1.00 | 0.00 | N | 0.97 | 0.03 | N | 0.69 | 0.31 | N | 0.78 | 0.22 | N | 1.00 | 0.00 | N |
| A1143T | N | 0.00 | 1.00 | P | 1.00 | 0.00 | N | 1.00 | 0.00 | N | 0.99 | 0.01 | N | 0.60 | 0.40 | N | 0.80 | 0.20 | N |
| Test set | | | | | | | | | | | | | | | | | | | |
| M434I | P | 0.00 | 1.00 | P | 0.85 | 0.15 | N | 0.06 | 0.94 | P | 0.10 | 0.90 | P | 0.22 | 0.78 | P | 0.20 | 0.80 | P |
| H445Q | P | 0.00 | 1.00 | P | 0.00 | 1.00 | P | 0.01 | 0.99 | P | 0.02 | 0.98 | P | 0.18 | 0.82 | P | 0.00 | 1.00 | P |
| T482P | P | 0.75 | 0.25 | N | 0.00 | 1.00 | P | 0.00 | 1.00 | P | 0.00 | 1.00 | P | 0.29 | 0.71 | P | 0.20 | 0.80 | P |
| T361I | P | 0.92 | 0.08 | N | 1.00 | 0.00 | N | 1.00 | 0.00 | N | 0.98 | 0.02 | N | 0.79 | 0.21 | N | 1.00 | 0.00 | N |
| S428R | P | 0.00 | 1.00 | P | 0.00 | 1.00 | P | 0.01 | 0.99 | P | 0.00 | 1.00 | P | 0.31 | 0.69 | P | 0.00 | 1.00 | P |
| L430Q | P | 0.04 | 0.96 | P | 0.00 | 1.00 | P | 0.00 | 1.00 | P | 0.02 | 0.98 | P | 0.10 | 0.90 | P | 0.00 | 1.00 | P |
| S431Y | P | 0.00 | 1.00 | P | 0.00 | 1.00 | P | 0.01 | 0.99 | P | 0.01 | 0.99 | P | 0.25 | 0.75 | P | 0.00 | 1.00 | P |
| Q432P | P | 0.04 | 0.96 | P | 0.00 | 1.00 | P | 0.00 | 1.00 | P | 0.00 | 1.00 | P | 0.21 | 0.79 | P | 0.00 | 1.00 | P |
| G442V | P | 0.00 | 1.00 | P | 0.00 | 1.00 | P | 0.01 | 0.99 | P | 0.00 | 1.00 | P | 0.23 | 0.77 | P | 0.00 | 1.00 | P |
| T444R | P | 0.00 | 1.00 | P | 0.00 | 1.00 | P | 0.00 | 1.00 | P | 0.00 | 1.00 | P | 0.21 | 0.79 | P | 0.00 | 1.00 | P |
| H445L | P | 0.00 | 1.00 | P | 0.00 | 1.00 | P | 0.00 | 1.00 | P | 0.01 | 0.99 | P | 0.20 | 0.80 | P | 0.00 | 1.00 | P |
| R448H | P | 0.04 | 0.96 | P | 0.00 | 1.00 | P | 0.45 | 0.55 | P | 0.00 | 1.00 | P | 0.23 | 0.77 | P | 0.00 | 1.00 | P |
| S450Q | P | 0.00 | 1.00 | P | 0.00 | 1.00 | P | 0.00 | 1.00 | P | 0.00 | 1.00 | P | 0.16 | 0.84 | P | 0.00 | 1.00 | P |
| G453A | P | 0.00 | 1.00 | P | 0.00 | 1.00 | P | 0.03 | 0.97 | P | 0.02 | 0.98 | P | 0.21 | 0.79 | P | 0.00 | 1.00 | P |
| G453V | P | 0.00 | 1.00 | P | 0.00 | 1.00 | P | 0.14 | 0.86 | P | 0.02 | 0.98 | P | 0.18 | 0.82 | P | 0.00 | 1.00 | P |
| I491L | P | 0.04 | 0.96 | P | 0.00 | 1.00 | P | 0.41 | 0.59 | P | 0.01 | 0.99 | P | 0.30 | 0.70 | P | 0.00 | 1.00 | P |
| I491N | P | 0.04 | 0.96 | P | 0.00 | 1.00 | P | 0.00 | 1.00 | P | 0.01 | 0.99 | P | 0.12 | 0.88 | P | 0.00 | 1.00 | P |
| I491S | P | 0.04 | 0.96 | P | 0.00 | 1.00 | P | 0.00 | 1.00 | P | 0.01 | 0.99 | P | 0.14 | 0.86 | P | 0.00 | 1.00 | P |
| D545E | P | 0.67 | 0.33 | N | 0.71 | 0.29 | N | 1.00 | 0.00 | N | 0.72 | 0.28 | N | 0.87 | 0.13 | N | 1.00 | 0.00 | N |
| D545N | P | 0.67 | 0.33 | N | 0.71 | 0.29 | N | 1.00 | 0.00 | N | 0.74 | 0.26 | N | 0.89 | 0.11 | N | 1.00 | 0.00 | N |
| R662H | P | 0.92 | 0.08 | N | 0.63 | 0.37 | N | 1.00 | 0.00 | N | 0.89 | 0.11 | N | 0.77 | 0.23 | N | 1.00 | 0.00 | N |
| H674R | P | 0.04 | 0.96 | P | 0.48 | 0.52 | P | 1.00 | 0.00 | N | 0.20 | 0.80 | P | 0.54 | 0.46 | N | 0.40 | 0.60 | P |
| H674Y | P | 0.04 | 0.96 | P | 0.00 | 1.00 | P | 0.00 | 1.00 | P | 0.05 | 0.95 | P | 0.44 | 0.56 | P | 0.00 | 1.00 | P |
| H723Y | P | 1.00 | 0.00 | N | 0.39 | 0.61 | P | 1.00 | 0.00 | N | 0.68 | 0.32 | N | 0.90 | 0.10 | N | 0.80 | 0.20 | N |
| H835P | P | 0.00 | 1.00 | P | 0.10 | 0.90 | P | 0.94 | 0.06 | N | 0.37 | 0.63 | P | 0.51 | 0.49 | N | 0.40 | 0.60 | P |
| D53N | N | 0.92 | 0.08 | N | 0.52 | 0.48 | N | 1.00 | 0.00 | N | 0.86 | 0.14 | N | 0.74 | 0.26 | N | 1.00 | 0.00 | N |
| V109I | N | 0.92 | 0.08 | N | 0.72 | 0.28 | N | 1.00 | 0.00 | N | 0.95 | 0.05 | N | 0.64 | 0.36 | N | 1.00 | 0.00 | N |
| L314V | N | 1.00 | 0.00 | N | 0.27 | 0.73 | P | 0.85 | 0.15 | N | 0.58 | 0.42 | N | 0.59 | 0.41 | N | 0.80 | 0.20 | N |
| A334D | N | 1.00 | 0.00 | N | 0.71 | 0.29 | N | 0.84 | 0.16 | N | 0.87 | 0.13 | N | 0.62 | 0.38 | N | 1.00 | 0.00 | N |
| D362H | N | 0.92 | 0.08 | N | 0.32 | 0.68 | P | 0.96 | 0.04 | N | 0.76 | 0.24 | N | 0.59 | 0.41 | N | 0.80 | 0.20 | N |
| P454L | N | 0.00 | 1.00 | P | 0.25 | 0.75 | P | 0.00 | 1.00 | P | 0.02 | 0.98 | P | 0.31 | 0.69 | P | 0.00 | 1.00 | P |
| E639Q | N | 0.92 | 0.08 | N | 1.00 | 0.00 | N | 1.00 | 0.00 | N | 0.97 | 0.03 | N | 0.75 | 0.25 | N | 1.00 | 0.00 | N |
| R661Q | N | 0.92 | 0.08 | N | 1.00 | 0.00 | N | 1.00 | 0.00 | N | 0.92 | 0.08 | N | 0.79 | 0.21 | N | 1.00 | 0.00 | N |
| E825G | N | 0.00 | 1.00 | P | 1.00 | 0.00 | N | 1.00 | 0.00 | N | 0.93 | 0.07 | N | 0.74 | 0.26 | N | 0.80 | 0.20 | N |
| A998V | N | 0.92 | 0.08 | N | 0.26 | 0.74 | P | 0.99 | 0.01 | N | 0.40 | 0.60 | P | 0.81 | 0.19 | N | 0.60 | 0.40 | N |
| V1117L | N | 0.92 | 0.08 | N | 0.34 | 0.66 | P | 0.94 | 0.06 | N | 0.53 | 0.47 | N | 0.55 | 0.45 | N | 0.80 | 0.20 | N |
| D109E | N | 0.67 | 0.33 | N | 0.71 | 0.29 | N | 1.00 | 0.00 | N | 0.98 | 0.02 | N | 0.77 | 0.23 | N | 1.00 | 0.00 | N |
| L1128Q | N | 0.92 | 0.08 | N | 0.67 | 0.33 | N | 0.62 | 0.38 | N | 0.86 | 0.14 | N | 0.40 | 0.60 | P | 0.80 | 0.20 | N |

Table S6. The parameters of 380 (20×19) RpoB mutants in Rif resistance-determining regions (RRDRs) or non-RRDRs.

| Mutation | Region | Distance | SS | Location | PremPS | PSSM | DCS | DOMH | P_L | P_FWY | P_RKDE | N_Hydro | N_Charg | SASA_pro | SASA_sol | DE | ΔE |
| --- | --- | --- | --- | --- | --- | --- | --- | --- | --- | --- | --- | --- | --- | --- | --- | --- | --- |
| L38A | non-RRDR | 39.45 | 1 | 0 | 0.53 | 0.46 | 0.63 | 0.65 | 0.00 | -0.44 | 0.05 | 0.03 | 0.01 | -0.96 | 0.10 | -8.83 | 0.05 |
| L38C | non-RRDR | 39.45 | 1 | 0 | 0.33 | 0.52 | 0.61 | 0.10 | 0.00 | -0.37 | 0.06 | -0.01 | -0.01 | -0.66 | 0.09 | -8.81 | 0.07 |
| L38D | non-RRDR | 39.45 | 1 | 0 | 0.96 | 0.74 | 0.59 | 0.85 | -0.01 | -0.42 | 0.04 | 0.01 | 0.00 | -0.95 | 0.09 | -8.9 | -0.02 |
| L38E | non-RRDR | 39.45 | 1 | 0 | 0.75 | 0.53 | 0.61 | 0.89 | -0.02 | -0.46 | 0.04 | 0.03 | 0.00 | -0.97 | 0.10 | -8.86 | 0.02 |
| L38F | non-RRDR | 39.45 | 1 | 0 | 0.12 | 0.50 | 0.44 | -0.29 | -0.03 | -0.17 | 0.05 | -0.04 | -0.01 | -0.43 | 0.09 | -8.84 | 0.04 |
| L38G | non-RRDR | 39.45 | 1 | 0 | 0.85 | 0.72 | 0.59 | 0.75 | 0.00 | -0.42 | 0.05 | 0.01 | 0.00 | -0.95 | 0.10 | -8.84 | 0.04 |
| L38H | non-RRDR | 39.45 | 1 | 0 | 0.56 | 0.50 | 0.61 | 0.71 | 0.00 | -0.45 | 0.06 | 0.03 | 0.00 | -0.99 | 0.10 | -8.86 | 0.02 |
| L38I | non-RRDR | 39.45 | 1 | 0 | -0.25 | -0.42 | -0.39 | 0.03 | -0.05 | 0.07 | -0.09 | 0.04 | 0.03 | 0.37 | 0.17 | -8.86 | 0.02 |
| L38K | non-RRDR | 39.45 | 1 | 0 | 0.63 | 0.53 | 0.64 | 0.73 | -0.01 | -0.45 | 0.05 | 0.03 | 0.00 | -0.98 | 0.10 | -8.85 | 0.03 |
| L38L | non-RRDR | 39.45 | 1 | 0 | -0.05 | -0.01 | 0.00 | 0.02 | -0.09 | -0.06 | -0.01 | 0.01 | 0.01 | -0.06 | 0.14 | -8.83 | 0.05 |
| L38M | non-RRDR | 39.45 | 1 | 0 | 0.25 | 0.44 | 0.35 | 0.00 | -0.03 | -0.17 | 0.06 | -0.03 | 0.00 | -0.44 | 0.08 | -8.86 | 0.02 |
| L38N | non-RRDR | 39.45 | 1 | 0 | 0.65 | 0.53 | 0.62 | 0.78 | -0.01 | -0.45 | 0.04 | 0.03 | 0.00 | -0.97 | 0.10 | -8.8 | 0.08 |
| L38P | non-RRDR | 39.45 | 1 | 0 | 0.51 | 0.51 | 0.61 | 0.64 | -0.01 | -0.45 | 0.05 | 0.03 | 0.00 | -0.99 | 0.11 | -8.86 | 0.02 |
| L38Q | non-RRDR | 39.45 | 1 | 0 | 0.45 | -0.21 | 0.87 | 0.69 | -0.03 | -0.28 | 0.00 | 0.00 | 0.01 | -0.75 | 0.14 | -8.87 | 0.01 |
| L38R | non-RRDR | 39.45 | 1 | 0 | 0.52 | 0.46 | 0.63 | 0.67 | 0.00 | -0.46 | 0.05 | 0.03 | 0.01 | -0.97 | 0.11 | -8.9 | -0.02 |
| L38S | non-RRDR | 39.45 | 1 | 0 | 0.54 | 0.51 | 0.64 | 0.63 | 0.00 | -0.44 | 0.05 | 0.02 | 0.00 | -0.98 | 0.11 | -8.88 | 0 |
| L38T | non-RRDR | 39.45 | 1 | 0 | 0.51 | 0.49 | 0.61 | 0.56 | 0.00 | -0.43 | 0.05 | 0.03 | 0.01 | -0.93 | 0.12 | -8.85 | 0.03 |
| L38V | non-RRDR | 39.45 | 1 | 0 | 0.08 | -0.01 | -0.11 | 0.17 | -0.09 | -0.06 | 0.01 | -0.01 | 0.01 | 0.02 | 0.14 | -8.81 | 0.07 |
| L38W | non-RRDR | 39.45 | 1 | 0 | 0.34 | 0.54 | 0.63 | 0.11 | 0.00 | -0.39 | 0.07 | -0.02 | 0.00 | -0.69 | 0.10 | -8.87 | 0.01 |
| L38Y | non-RRDR | 39.45 | 1 | 0 | 0.21 | 0.51 | 0.59 | -0.25 | -0.03 | -0.22 | 0.06 | -0.02 | -0.02 | -0.50 | 0.09 | -8.83 | 0.05 |
| K119A | non-RRDR | 26.26 | 4 | 0 | 0.69 | 0.61 | 0.67 | -0.26 | 0.00 | -0.24 | 0.09 | 0.01 | 0.00 | -0.29 | 0.10 | -8.88 | 0 |
| K119C | non-RRDR | 26.26 | 4 | 0 | 0.51 | 0.54 | 0.63 | -0.39 | -0.01 | -0.20 | 0.13 | -0.01 | 0.00 | -0.28 | 0.09 | -8.89 | -0.01 |
| K119D | non-RRDR | 26.26 | 4 | 0 | 0.73 | 0.56 | 0.63 | 0.04 | 0.00 | -0.35 | 0.09 | 0.02 | 0.04 | -0.41 | 0.11 | -8.89 | -0.01 |
| K119E | non-RRDR | 26.26 | 4 | 0 | 0.68 | 0.51 | 0.58 | 0.00 | 0.00 | -0.30 | 0.08 | 0.03 | 0.02 | -0.35 | 0.11 | -8.83 | 0.05 |
| K119F | non-RRDR | 26.26 | 4 | 0 | 0.49 | 0.59 | 0.67 | -0.60 | 0.00 | -0.17 | 0.15 | 0.00 | 0.00 | -0.23 | 0.08 | -8.9 | -0.02 |
| K119G | non-RRDR | 26.26 | 4 | 0 | 1.00 | 0.58 | 0.68 | 0.04 | 0.00 | -0.25 | 0.11 | 0.01 | 0.03 | -0.31 | 0.12 | -8.83 | 0.05 |
| K119H | non-RRDR | 26.26 | 4 | 0 | 0.13 | -0.55 | 0.98 | 0.00 | -0.01 | -0.17 | 0.02 | 0.01 | -0.01 | -0.25 | 0.12 | -8.88 | 0 |
| K119I | non-RRDR | 26.26 | 4 | 0 | 0.47 | 0.58 | 0.68 | -0.63 | 0.01 | -0.17 | 0.15 | -0.01 | 0.00 | -0.23 | 0.08 | -8.88 | 0 |
| K119K | non-RRDR | 26.26 | 4 | 0 | -0.10 | -0.45 | 0.24 | 0.04 | -0.04 | -0.02 | -0.03 | 0.01 | -0.01 | 0.02 | 0.14 | -8.87 | 0.01 |
| K119L | non-RRDR | 26.26 | 4 | 0 | 0.50 | 0.60 | 0.66 | -0.63 | 0.00 | -0.18 | 0.16 | 0.00 | 0.01 | -0.21 | 0.09 | -8.92 | -0.04 |
| K119M | non-RRDR | 26.26 | 4 | 0 | 0.44 | 0.58 | 0.64 | -0.61 | -0.01 | -0.18 | 0.14 | 0.00 | 0.00 | -0.21 | 0.08 | -8.87 | 0.01 |
| K119N | non-RRDR | 26.26 | 4 | 0 | 0.75 | 0.52 | 0.59 | -0.01 | 0.00 | -0.27 | 0.08 | 0.01 | 0.02 | -0.31 | 0.11 | -8.87 | 0.01 |
| K119P | non-RRDR | 26.26 | 4 | 0 | 0.84 | 0.59 | 0.66 | -0.13 | 0.01 | -0.26 | 0.11 | 0.01 | 0.02 | -0.29 | 0.11 | -8.88 | 0 |
| K119Q | non-RRDR | 26.26 | 4 | 0 | 0.45 | 0.41 | 0.36 | 0.00 | -0.01 | -0.23 | 0.07 | 0.01 | 0.00 | -0.26 | 0.09 | -8.86 | 0.02 |
| K119R | non-RRDR | 26.26 | 4 | 0 | -0.38 | -0.43 | -0.32 | 0.03 | -0.06 | 0.08 | -0.09 | 0.00 | 0.01 | 0.31 | 0.09 | -8.89 | -0.01 |
| K119S | non-RRDR | 26.26 | 4 | 0 | 0.76 | 0.55 | 0.61 | -0.09 | 0.00 | -0.25 | 0.10 | 0.01 | 0.02 | -0.29 | 0.11 | -8.91 | -0.03 |
| K119T | non-RRDR | 26.26 | 4 | 0 | 0.62 | 0.57 | 0.67 | -0.28 | 0.00 | -0.24 | 0.09 | 0.00 | 0.00 | -0.29 | 0.10 | -8.84 | 0.04 |
| K119V | non-RRDR | 26.26 | 4 | 0 | 0.61 | 0.60 | 0.67 | -0.51 | 0.01 | -0.17 | 0.16 | 0.00 | -0.01 | -0.23 | 0.09 | -8.85 | 0.03 |
| K119W | non-RRDR | 26.26 | 4 | 0 | 0.55 | 0.59 | 0.63 | -0.38 | 0.00 | -0.21 | 0.13 | 0.00 | 0.00 | -0.28 | 0.09 | -8.83 | 0.05 |
| K119Y | non-RRDR | 26.26 | 4 | 0 | 0.43 | 0.57 | 0.66 | -0.66 | 0.00 | -0.18 | 0.17 | 0.00 | 0.00 | -0.22 | 0.08 | -8.9 | -0.02 |
| E132A | non-RRDR | 44.24 | 2 | 0 | 0.47 | 0.56 | 0.56 | -0.40 | 0.01 | -0.26 | 0.09 | 0.01 | -0.09 | -0.09 | 0.09 | -8.83 | 0.05 |
| E132C | non-RRDR | 44.24 | 2 | 0 | 0.55 | 0.56 | 0.69 | -0.51 | 0.01 | -0.22 | 0.12 | -0.01 | -0.09 | -0.07 | 0.07 | -8.75 | 0.13 |
| E132D | non-RRDR | 44.24 | 2 | 0 | 0.50 | 0.52 | 0.36 | -0.05 | 0.00 | -0.27 | 0.09 | -0.01 | -0.07 | -0.15 | 0.07 | -8.84 | 0.04 |
| E132E | non-RRDR | 44.24 | 2 | 0 | -0.13 | -0.42 | 0.20 | 0.03 | -0.06 | -0.06 | -0.04 | 0.03 | 0.03 | 0.02 | 0.13 | -8.87 | 0.01 |
| E132F | non-RRDR | 44.24 | 2 | 0 | 0.55 | 0.57 | 0.68 | -0.58 | 0.00 | -0.21 | 0.14 | 0.01 | -0.05 | -0.08 | 0.09 | -8.82 | 0.06 |
| E132G | non-RRDR | 44.24 | 2 | 0 | 0.69 | 0.60 | 0.63 | -0.22 | 0.02 | -0.27 | 0.06 | 0.00 | -0.09 | -0.13 | 0.10 | -8.85 | 0.03 |
| E132H | non-RRDR | 44.24 | 2 | 0 | 0.49 | 0.51 | 0.41 | -0.21 | 0.01 | -0.21 | 0.04 | 0.01 | -0.07 | -0.11 | 0.11 | -8.89 | -0.01 |
| E132I | non-RRDR | 44.24 | 2 | 0 | 0.46 | 0.55 | 0.68 | -0.69 | 0.01 | -0.21 | 0.14 | 0.01 | -0.03 | -0.07 | 0.08 | -8.88 | 0 |
| E132K | non-RRDR | 44.24 | 2 | 0 | 0.06 | -0.04 | 0.41 | -0.21 | -0.06 | -0.10 | -0.01 | 0.02 | -0.03 | -0.07 | 0.14 | -8.88 | 0 |
| E132L | non-RRDR | 44.24 | 2 | 0 | 0.46 | 0.57 | 0.66 | -0.69 | 0.01 | -0.22 | 0.14 | 0.01 | -0.04 | -0.05 | 0.08 | -8.82 | 0.06 |
| E132M | non-RRDR | 44.24 | 2 | 0 | 0.28 | 0.55 | 0.56 | -0.75 | 0.02 | -0.21 | 0.10 | 0.01 | -0.04 | -0.04 | 0.08 | -8.84 | 0.04 |
| E132N | non-RRDR | 44.24 | 2 | 0 | 0.45 | 0.51 | 0.37 | -0.23 | 0.02 | -0.21 | 0.04 | 0.01 | -0.06 | -0.12 | 0.12 | -8.85 | 0.03 |
| E132P | non-RRDR | 44.24 | 2 | 0 | 0.55 | 0.60 | 0.58 | -0.38 | 0.01 | -0.25 | 0.09 | 0.00 | -0.08 | -0.10 | 0.09 | -8.83 | 0.05 |
| E132Q | non-RRDR | 44.24 | 2 | 0 | 0.05 | -0.47 | 0.64 | -0.15 | 0.00 | -0.11 | 0.01 | 0.03 | -0.01 | -0.05 | 0.16 | -8.87 | 0.01 |
| E132R | non-RRDR | 44.24 | 2 | 0 | -0.57 | -0.45 | -0.12 | -0.26 | -0.08 | 0.00 | -0.09 | 0.03 | 0.02 | 0.20 | 0.18 | -8.9 | -0.02 |
| E132S | non-RRDR | 44.24 | 2 | 0 | 0.53 | 0.52 | 0.51 | -0.27 | 0.01 | -0.24 | 0.07 | 0.01 | -0.08 | -0.09 | 0.09 | -8.83 | 0.05 |
| E132T | non-RRDR | 44.24 | 2 | 0 | 0.45 | 0.52 | 0.57 | -0.41 | 0.01 | -0.26 | 0.09 | 0.01 | -0.08 | -0.08 | 0.09 | -8.9 | -0.02 |
| E132V | non-RRDR | 44.24 | 2 | 0 | 0.41 | 0.57 | 0.66 | -0.72 | 0.01 | -0.23 | 0.14 | 0.00 | -0.04 | -0.05 | 0.08 | -8.79 | 0.09 |
| E132W | non-RRDR | 44.24 | 2 | 0 | 0.47 | 0.57 | 0.69 | -0.65 | 0.01 | -0.22 | 0.14 | -0.01 | -0.08 | -0.06 | 0.08 | -8.87 | 0.01 |
| E132Y | non-RRDR | 44.24 | 2 | 0 | 0.48 | 0.57 | 0.65 | -0.64 | -0.01 | -0.21 | 0.13 | 0.01 | -0.03 | -0.07 | 0.09 | -8.88 | 0 |
| I185A | non-RRDR | 38.45 | 2 | 0 | 0.61 | 0.17 | 0.14 | 0.72 | -0.03 | -0.22 | 0.02 | 0.02 | 0.04 | -0.41 | 0.14 | -8.82 | 0.06 |
| I185C | non-RRDR | 38.45 | 2 | 0 | 0.53 | 0.55 | 0.32 | 0.17 | -0.01 | -0.28 | 0.07 | -0.01 | 0.03 | -0.38 | 0.08 | -8.85 | 0.03 |
| I185D | non-RRDR | 38.45 | 2 | 0 | 1.18 | 0.52 | 0.56 | 0.82 | 0.01 | -0.39 | 0.05 | 0.03 | 0.09 | -0.65 | 0.14 | -8.83 | 0.05 |
| I185E | non-RRDR | 38.45 | 2 | 0 | 1.00 | 0.47 | 0.26 | 0.81 | 0.01 | -0.32 | 0.06 | 0.04 | 0.09 | -0.55 | 0.15 | -8.89 | -0.01 |
| I185F | non-RRDR | 38.45 | 2 | 0 | 0.25 | 0.40 | 0.34 | -0.22 | -0.02 | -0.15 | 0.02 | -0.03 | 0.00 | -0.21 | 0.10 | -8.83 | 0.05 |
| I185G | non-RRDR | 38.45 | 2 | 0 | 1.21 | 0.52 | 0.64 | 0.81 | 0.01 | -0.41 | 0.06 | 0.03 | 0.09 | -0.67 | 0.14 | -8.93 | -0.05 |
| I185H | non-RRDR | 38.45 | 2 | 0 | 0.56 | -0.52 | 0.85 | 0.66 | -0.02 | -0.18 | 0.01 | 0.00 | 0.02 | -0.42 | 0.16 | -8.88 | 0 |
| I185I | non-RRDR | 38.45 | 2 | 0 | -0.13 | -0.28 | 0.12 | 0.03 | -0.07 | -0.05 | -0.03 | 0.01 | 0.02 | 0.00 | 0.12 | -8.79 | 0.09 |
| I185K | non-RRDR | 38.45 | 2 | 0 | 0.80 | 0.12 | 0.36 | 0.77 | 0.00 | -0.25 | 0.03 | 0.02 | 0.06 | -0.46 | 0.15 | -8.81 | 0.07 |
| I185L | non-RRDR | 38.45 | 2 | 0 | 0.16 | 0.30 | 0.21 | -0.03 | -0.06 | -0.16 | 0.03 | -0.02 | 0.00 | -0.20 | 0.08 | -8.9 | -0.02 |
| I185M | non-RRDR | 38.45 | 2 | 0 | 0.11 | -0.05 | 0.39 | 0.07 | -0.11 | -0.13 | 0.00 | -0.03 | 0.00 | -0.15 | 0.12 | -8.83 | 0.05 |
| I185N | non-RRDR | 38.45 | 2 | 0 | 1.09 | 0.45 | 0.46 | 0.83 | 0.02 | -0.38 | 0.06 | 0.03 | 0.10 | -0.62 | 0.14 | -8.86 | 0.02 |
| I185P | non-RRDR | 38.45 | 2 | 0 | 0.64 | -0.19 | 0.60 | 0.67 | -0.05 | -0.17 | 0.01 | 0.00 | 0.04 | -0.42 | 0.17 | -8.85 | 0.03 |
| I185Q | non-RRDR | 38.45 | 2 | 0 | 0.78 | 0.12 | 0.37 | 0.76 | 0.00 | -0.26 | 0.02 | 0.02 | 0.06 | -0.46 | 0.15 | -8.9 | -0.02 |
| I185R | non-RRDR | 38.45 | 2 | 0 | 0.77 | 0.12 | 0.37 | 0.74 | 0.00 | -0.25 | 0.03 | 0.01 | 0.06 | -0.46 | 0.15 | -8.85 | 0.03 |
| I185S | non-RRDR | 38.45 | 2 | 0 | 0.96 | 0.39 | 0.24 | 0.76 | 0.01 | -0.27 | 0.08 | 0.02 | 0.09 | -0.51 | 0.16 | -8.87 | 0.01 |
| I185T | non-RRDR | 38.45 | 2 | 0 | 0.48 | -0.18 | 0.48 | 0.59 | -0.07 | -0.17 | 0.00 | 0.00 | 0.04 | -0.38 | 0.17 | -8.91 | -0.03 |
| I185V | non-RRDR | 38.45 | 2 | 0 | 0.11 | -0.01 | -0.10 | 0.22 | -0.09 | -0.06 | 0.02 | -0.01 | 0.00 | 0.00 | 0.14 | -8.85 | 0.03 |
| I185W | non-RRDR | 38.45 | 2 | 0 | 0.68 | 0.53 | 0.64 | 0.14 | -0.01 | -0.37 | 0.05 | -0.03 | 0.04 | -0.38 | 0.07 | -8.87 | 0.01 |
| I185Y | non-RRDR | 38.45 | 2 | 0 | -0.05 | -0.07 | 0.59 | -0.23 | -0.13 | -0.10 | -0.06 | -0.02 | -0.01 | -0.16 | 0.13 | -8.88 | 0 |
| G236A | non-RRDR | 52.58 | 4 | 0 | 1.02 | 0.55 | 0.84 | -0.17 | -0.01 | -0.25 | 0.08 | 0.15 | 0.06 | -0.05 | -0.17 | -8.82 | 0.06 |
| G236C | non-RRDR | 52.58 | 4 | 0 | 1.28 | 0.60 | 1.03 | -0.36 | -0.03 | -0.18 | 0.09 | 0.15 | 0.07 | -0.03 | -0.05 | -8.9 | -0.02 |
| G236D | non-RRDR | 52.58 | 4 | 0 | 0.94 | 0.54 | 0.89 | 0.10 | -0.01 | -0.37 | 0.01 | 0.09 | 0.03 | -0.13 | -0.21 | -8.82 | 0.06 |
| G236E | non-RRDR | 52.58 | 4 | 0 | 1.12 | 0.55 | 0.96 | 0.07 | -0.01 | -0.29 | 0.03 | 0.10 | 0.03 | -0.12 | -0.21 | -8.83 | 0.05 |
| G236F | non-RRDR | 52.58 | 4 | 0 | 1.23 | 0.63 | 1.04 | -0.56 | -0.01 | -0.14 | 0.11 | 0.15 | 0.07 | -0.03 | -0.04 | -8.84 | 0.04 |
| G236G | non-RRDR | 52.58 | 4 | 0 | -0.21 | -0.41 | 0.36 | 0.12 | 0.01 | -0.10 | 0.00 | 0.01 | 0.00 | 0.10 | -0.31 | -8.91 | -0.03 |
| G236H | non-RRDR | 52.58 | 4 | 0 | 1.17 | 0.60 | 0.94 | -0.04 | -0.01 | -0.27 | 0.05 | 0.14 | 0.04 | -0.10 | -0.19 | -8.85 | 0.03 |
| G236I | non-RRDR | 52.58 | 4 | 0 | 1.28 | 0.68 | 1.04 | -0.58 | 0.01 | -0.15 | 0.12 | 0.14 | 0.07 | -0.02 | -0.04 | -8.82 | 0.06 |
| G236K | non-RRDR | 52.58 | 4 | 0 | 1.13 | 0.56 | 0.96 | -0.04 | -0.01 | -0.26 | 0.05 | 0.13 | 0.03 | -0.09 | -0.19 | -8.87 | 0.01 |
| G236L | non-RRDR | 52.58 | 4 | 0 | 1.22 | 0.60 | 1.07 | -0.58 | 0.00 | -0.15 | 0.12 | 0.15 | 0.08 | -0.03 | -0.04 | -8.86 | 0.02 |
| G236M | non-RRDR | 52.58 | 4 | 0 | 1.27 | 0.60 | 1.08 | -0.55 | 0.00 | -0.15 | 0.12 | 0.16 | 0.08 | -0.02 | -0.04 | -8.89 | -0.01 |
| G236N | non-RRDR | 52.58 | 4 | 0 | 0.98 | 0.51 | 0.89 | -0.02 | 0.01 | -0.30 | 0.03 | 0.09 | 0.03 | -0.06 | -0.20 | -8.87 | 0.01 |
| G236P | non-RRDR | 52.58 | 4 | 0 | 1.14 | 0.60 | 0.91 | -0.11 | -0.01 | -0.26 | 0.06 | 0.14 | 0.06 | -0.09 | -0.17 | -8.82 | 0.06 |
| G236Q | non-RRDR | 52.58 | 4 | 0 | 1.18 | 0.61 | 0.95 | -0.01 | 0.00 | -0.28 | 0.03 | 0.12 | 0.04 | -0.10 | -0.20 | -8.77 | 0.11 |
| G236R | non-RRDR | 52.58 | 4 | 0 | 1.15 | 0.60 | 0.94 | -0.07 | -0.01 | -0.26 | 0.06 | 0.13 | 0.05 | -0.10 | -0.19 | -8.81 | 0.07 |
| G236S | non-RRDR | 52.58 | 4 | 0 | 0.98 | 0.54 | 0.84 | -0.06 | -0.02 | -0.29 | 0.06 | 0.13 | 0.04 | -0.08 | -0.20 | -8.77 | 0.11 |
| G236T | non-RRDR | 52.58 | 4 | 0 | 1.19 | 0.58 | 0.95 | -0.20 | -0.02 | -0.22 | 0.08 | 0.15 | 0.06 | -0.04 | -0.14 | -8.8 | 0.08 |
| G236V | non-RRDR | 52.58 | 4 | 0 | 1.53 | 0.65 | 1.06 | -0.35 | -0.01 | -0.13 | 0.13 | 0.15 | 0.09 | -0.03 | -0.02 | -8.79 | 0.09 |
| G236W | non-RRDR | 52.58 | 4 | 0 | 1.22 | 0.59 | 0.99 | -0.38 | -0.03 | -0.17 | 0.09 | 0.15 | 0.07 | -0.03 | -0.06 | -8.88 | 0 |
| G236Y | non-RRDR | 52.58 | 4 | 0 | 1.18 | 0.64 | 1.00 | -0.62 | 0.01 | -0.14 | 0.12 | 0.14 | 0.07 | -0.02 | -0.02 | -8.85 | 0.03 |
| E244A | non-RRDR | 56.54 | 3 | 0 | 0.10 | 0.25 | 0.63 | -0.27 | -0.04 | -0.15 | 0.08 | 0.00 | 0.00 | -0.48 | 0.09 | -8.88 | 0 |
| E244C | non-RRDR | 56.54 | 3 | 0 | 0.33 | 0.55 | 0.68 | -0.45 | -0.02 | -0.17 | 0.14 | -0.02 | -0.02 | -0.44 | 0.07 | -8.86 | 0.02 |
| E244D | non-RRDR | 56.54 | 3 | 0 | 0.09 | -0.50 | 0.77 | 0.05 | -0.02 | -0.11 | 0.01 | 0.03 | 0.02 | -0.27 | 0.11 | -8.8 | 0.08 |
| E244E | non-RRDR | 56.54 | 3 | 0 | -0.09 | -0.44 | 0.24 | 0.02 | -0.04 | -0.04 | -0.04 | 0.03 | 0.02 | 0.02 | 0.14 | -8.93 | -0.05 |
| E244F | non-RRDR | 56.54 | 3 | 0 | 0.29 | 0.55 | 0.67 | -0.55 | -0.02 | -0.16 | 0.14 | 0.00 | -0.02 | -0.41 | 0.08 | -8.83 | 0.05 |
| E244G | non-RRDR | 56.54 | 3 | 0 | 0.29 | 0.49 | 0.63 | -0.23 | -0.02 | -0.22 | 0.09 | -0.01 | -0.01 | -0.52 | 0.09 | -8.87 | 0.01 |
| E244H | non-RRDR | 56.54 | 3 | 0 | 0.10 | 0.23 | 0.63 | -0.22 | -0.05 | -0.16 | 0.06 | 0.01 | 0.00 | -0.50 | 0.09 | -8.87 | 0.01 |
| E244I | non-RRDR | 56.54 | 3 | 0 | 0.29 | 0.56 | 0.69 | -0.62 | -0.02 | -0.16 | 0.15 | 0.00 | -0.01 | -0.38 | 0.08 | -8.82 | 0.06 |
| E244K | non-RRDR | 56.54 | 3 | 0 | 0.09 | 0.24 | 0.62 | -0.23 | -0.05 | -0.16 | 0.05 | 0.02 | 0.00 | -0.48 | 0.09 | -8.86 | 0.02 |
| E244L | non-RRDR | 56.54 | 3 | 0 | 0.30 | 0.57 | 0.69 | -0.62 | -0.02 | -0.16 | 0.15 | 0.00 | -0.01 | -0.39 | 0.08 | -8.85 | 0.03 |
| E244M | non-RRDR | 56.54 | 3 | 0 | 0.22 | 0.54 | 0.66 | -0.62 | -0.02 | -0.17 | 0.16 | 0.00 | -0.01 | -0.39 | 0.09 | -8.88 | 0 |
| E244N | non-RRDR | 56.54 | 3 | 0 | -0.03 | -0.36 | 0.89 | -0.14 | -0.07 | -0.12 | 0.00 | 0.02 | 0.00 | -0.37 | 0.12 | -8.81 | 0.07 |
| E244P | non-RRDR | 56.54 | 3 | 0 | 0.29 | 0.57 | 0.64 | -0.33 | -0.04 | -0.20 | 0.12 | -0.01 | -0.02 | -0.51 | 0.08 | -8.89 | -0.01 |
| E244Q | non-RRDR | 56.54 | 3 | 0 | 0.01 | 0.25 | 0.48 | -0.22 | -0.05 | -0.14 | 0.02 | 0.02 | 0.00 | -0.44 | 0.08 | -8.84 | 0.04 |
| E244R | non-RRDR | 56.54 | 3 | 0 | 0.20 | 0.38 | 0.61 | -0.23 | -0.03 | -0.18 | 0.08 | 0.00 | -0.01 | -0.51 | 0.10 | -8.89 | -0.01 |
| E244S | non-RRDR | 56.54 | 3 | 0 | 0.18 | 0.39 | 0.59 | -0.25 | -0.03 | -0.18 | 0.07 | 0.00 | -0.02 | -0.49 | 0.10 | -8.87 | 0.01 |
| E244T | non-RRDR | 56.54 | 3 | 0 | 0.24 | 0.49 | 0.65 | -0.34 | -0.03 | -0.20 | 0.11 | -0.01 | -0.01 | -0.50 | 0.08 | -8.88 | 0 |
| E244V | non-RRDR | 56.54 | 3 | 0 | 0.25 | 0.56 | 0.66 | -0.63 | -0.02 | -0.17 | 0.15 | 0.00 | -0.01 | -0.38 | 0.08 | -8.86 | 0.02 |
| E244W | non-RRDR | 56.54 | 3 | 0 | 0.30 | 0.55 | 0.71 | -0.58 | -0.01 | -0.16 | 0.14 | -0.01 | -0.03 | -0.39 | 0.08 | -8.84 | 0.04 |
| E244Y | non-RRDR | 56.54 | 3 | 0 | 0.27 | 0.54 | 0.68 | -0.57 | -0.03 | -0.17 | 0.15 | 0.00 | -0.01 | -0.41 | 0.09 | -8.84 | 0.04 |
| I336A | non-RRDR | 42.92 | 3 | 1 | 2.30 | 0.47 | 0.61 | 0.87 | -0.08 | -0.23 | -0.06 | 0.00 | 0.01 | 0.54 | 0.18 | -8.82 | 0.06 |
| I336C | non-RRDR | 42.92 | 3 | 1 | 1.53 | 0.57 | 0.64 | 0.06 | -0.04 | -0.27 | -0.04 | -0.03 | -0.01 | 0.50 | 0.15 | -8.87 | 0.01 |
| I336D | non-RRDR | 42.92 | 3 | 1 | 2.35 | 0.52 | 0.65 | 0.96 | -0.07 | -0.26 | -0.08 | -0.03 | -0.01 | 0.50 | 0.17 | -8.87 | 0.01 |
| I336E | non-RRDR | 42.92 | 3 | 1 | 2.32 | 0.52 | 0.65 | 0.96 | -0.07 | -0.27 | -0.09 | -0.03 | -0.01 | 0.49 | 0.17 | -8.89 | -0.01 |
| I336F | non-RRDR | 42.92 | 3 | 1 | 1.02 | 0.48 | 0.56 | -0.42 | 0.00 | -0.17 | 0.01 | -0.03 | -0.03 | 0.46 | 0.17 | -8.89 | -0.01 |
| I336G | non-RRDR | 42.92 | 3 | 1 | 2.33 | 0.51 | 0.71 | 0.90 | -0.08 | -0.26 | -0.08 | -0.04 | -0.01 | 0.50 | 0.17 | -8.81 | 0.07 |
| I336H | non-RRDR | 42.92 | 3 | 1 | 1.92 | 0.04 | 0.82 | 0.82 | -0.02 | -0.27 | -0.11 | -0.03 | 0.01 | 0.50 | 0.16 | -8.79 | 0.09 |
| I336I | non-RRDR | 42.92 | 3 | 1 | -0.18 | -0.51 | 0.31 | 0.01 | -0.03 | -0.10 | 0.00 | 0.01 | 0.03 | -0.05 | 0.15 | -8.88 | 0 |
| I336K | non-RRDR | 42.92 | 3 | 1 | 2.24 | 0.47 | 0.65 | 0.89 | -0.07 | -0.27 | -0.08 | -0.02 | -0.01 | 0.51 | 0.18 | -8.83 | 0.05 |
| I336L | non-RRDR | 42.92 | 3 | 1 | 0.81 | -0.33 | 0.74 | -0.08 | 0.07 | -0.13 | 0.06 | 0.02 | 0.02 | 0.26 | 0.17 | -8.86 | 0.02 |
| I336M | non-RRDR | 42.92 | 3 | 1 | 1.00 | -0.34 | 0.81 | 0.04 | 0.07 | -0.13 | 0.08 | 0.01 | 0.01 | 0.29 | 0.16 | -8.82 | 0.06 |
| I336N | non-RRDR | 42.92 | 3 | 1 | 2.28 | 0.47 | 0.65 | 0.91 | -0.07 | -0.27 | -0.08 | -0.02 | -0.01 | 0.51 | 0.18 | -8.83 | 0.05 |
| I336P | non-RRDR | 42.92 | 3 | 1 | 2.26 | 0.52 | 0.65 | 0.86 | -0.07 | -0.26 | -0.09 | -0.03 | 0.00 | 0.51 | 0.17 | -8.81 | 0.07 |
| I336Q | non-RRDR | 42.92 | 3 | 1 | 2.29 | 0.47 | 0.67 | 0.91 | -0.07 | -0.27 | -0.09 | -0.01 | -0.01 | 0.51 | 0.18 | -8.81 | 0.07 |
| I336R | non-RRDR | 42.92 | 3 | 1 | 2.15 | 0.34 | 0.70 | 0.85 | -0.06 | -0.25 | -0.10 | -0.03 | -0.01 | 0.53 | 0.18 | -8.8 | 0.08 |
| I336S | non-RRDR | 42.92 | 3 | 1 | 2.26 | 0.48 | 0.66 | 0.87 | -0.07 | -0.26 | -0.08 | -0.02 | 0.00 | 0.51 | 0.18 | -8.83 | 0.05 |
| I336T | non-RRDR | 42.92 | 3 | 1 | 2.19 | 0.48 | 0.57 | 0.82 | -0.08 | -0.22 | -0.07 | -0.01 | 0.00 | 0.53 | 0.18 | -8.84 | 0.04 |
| I336V | non-RRDR | 42.92 | 3 | 1 | 0.71 | -0.33 | 0.39 | 0.22 | 0.06 | -0.15 | 0.12 | 0.02 | 0.01 | 0.19 | 0.17 | -8.82 | 0.06 |
| I336W | non-RRDR | 42.92 | 3 | 1 | 1.52 | 0.61 | 0.67 | 0.07 | -0.05 | -0.29 | -0.06 | -0.04 | 0.00 | 0.47 | 0.15 | -8.91 | -0.03 |
| I336Y | non-RRDR | 42.92 | 3 | 1 | 1.31 | 0.57 | 0.75 | -0.38 | -0.02 | -0.24 | 0.04 | -0.04 | -0.01 | 0.45 | 0.19 | -8.88 | 0 |
| R373A | non-RRDR | 18.36 | 2 | 1 | 0.98 | 0.53 | 0.61 | -0.21 | -0.01 | -0.36 | 0.09 | -0.07 | 0.05 | 0.06 | 0.29 | -8.8 | 0.08 |
| R373C | non-RRDR | 18.36 | 2 | 1 | 0.92 | 0.50 | 0.75 | -0.45 | -0.02 | -0.26 | 0.11 | -0.04 | 0.04 | 0.05 | 0.25 | -8.85 | 0.03 |
| R373D | non-RRDR | 18.36 | 2 | 1 | 0.95 | 0.51 | 0.67 | 0.09 | -0.04 | -0.50 | 0.00 | -0.03 | 0.07 | -0.04 | 0.23 | -8.87 | 0.01 |
| R373E | non-RRDR | 18.36 | 2 | 1 | 0.92 | 0.42 | 0.65 | -0.01 | 0.00 | -0.42 | 0.00 | -0.04 | 0.05 | 0.00 | 0.27 | -8.86 | 0.02 |
| R373F | non-RRDR | 18.36 | 2 | 1 | 0.78 | 0.52 | 0.78 | -0.75 | -0.02 | -0.19 | 0.15 | -0.03 | 0.03 | 0.02 | 0.28 | -8.92 | -0.04 |
| R373G | non-RRDR | 18.36 | 2 | 1 | 1.07 | 0.54 | 0.64 | -0.07 | -0.05 | -0.38 | 0.05 | -0.06 | 0.06 | 0.05 | 0.30 | -8.91 | -0.03 |
| R373H | non-RRDR | 18.36 | 2 | 1 | 1.09 | 0.44 | 0.66 | -0.04 | 0.00 | -0.36 | 0.03 | -0.06 | 0.05 | 0.05 | 0.32 | -8.89 | -0.01 |
| R373I | non-RRDR | 18.36 | 2 | 1 | 0.79 | 0.52 | 0.79 | -0.72 | -0.01 | -0.19 | 0.14 | -0.05 | 0.03 | 0.01 | 0.27 | -8.88 | 0 |
| R373K | non-RRDR | 18.36 | 2 | 1 | 0.96 | -0.08 | 0.88 | 0.02 | 0.00 | -0.23 | -0.01 | -0.06 | 0.00 | 0.10 | 0.32 | -8.9 | -0.02 |
| R373L | non-RRDR | 18.36 | 2 | 1 | 0.75 | 0.54 | 0.72 | -0.71 | -0.01 | -0.22 | 0.14 | -0.05 | 0.03 | 0.03 | 0.28 | -8.86 | 0.02 |
| R373M | non-RRDR | 18.36 | 2 | 1 | 0.88 | 0.57 | 0.70 | -0.60 | -0.01 | -0.22 | 0.16 | -0.06 | 0.01 | 0.04 | 0.30 | -8.9 | -0.02 |
| R373N | non-RRDR | 18.36 | 2 | 1 | 1.15 | 0.54 | 0.66 | -0.03 | -0.01 | -0.39 | 0.04 | -0.05 | 0.05 | 0.04 | 0.29 | -8.86 | 0.02 |
| R373P | non-RRDR | 18.36 | 2 | 1 | 1.00 | 0.53 | 0.63 | -0.18 | -0.03 | -0.36 | 0.06 | -0.06 | 0.06 | 0.05 | 0.30 | -8.86 | 0.02 |
| R373Q | non-RRDR | 18.36 | 2 | 1 | 1.07 | 0.43 | 0.67 | -0.03 | 0.00 | -0.37 | 0.02 | -0.06 | 0.05 | 0.04 | 0.32 | -8.88 | 0 |
| R373R | non-RRDR | 18.36 | 2 | 1 | 0.01 | -0.44 | 0.21 | 0.01 | -0.03 | -0.06 | -0.03 | 0.00 | 0.00 | 0.11 | 0.23 | -8.87 | 0.01 |
| R373S | non-RRDR | 18.36 | 2 | 1 | 1.12 | 0.55 | 0.63 | -0.08 | -0.02 | -0.38 | 0.07 | -0.06 | 0.06 | 0.06 | 0.30 | -8.9 | -0.02 |
| R373T | non-RRDR | 18.36 | 2 | 1 | 0.92 | 0.54 | 0.61 | -0.29 | -0.01 | -0.34 | 0.09 | -0.06 | 0.03 | 0.07 | 0.29 | -8.88 | 0 |
| R373V | non-RRDR | 18.36 | 2 | 1 | 0.98 | 0.54 | 0.80 | -0.55 | -0.01 | -0.22 | 0.15 | -0.06 | 0.02 | 0.02 | 0.28 | -8.91 | -0.03 |
| R373W | non-RRDR | 18.36 | 2 | 1 | 0.92 | 0.49 | 0.75 | -0.49 | -0.03 | -0.24 | 0.11 | -0.04 | 0.05 | 0.07 | 0.26 | -8.93 | -0.05 |
| R373Y | non-RRDR | 18.36 | 2 | 1 | 0.80 | 0.59 | 0.75 | -0.77 | -0.01 | -0.21 | 0.16 | -0.06 | 0.02 | 0.04 | 0.29 | -8.9 | -0.02 |
| T400A | non-RRDR | 46.85 | 3 | 0 | 0.46 | 0.42 | 0.58 | 0.03 | 0.00 | -0.24 | 0.05 | -0.03 | -0.02 | -0.26 | -0.08 | -8.86 | 0.02 |
| T400C | non-RRDR | 46.85 | 3 | 0 | 0.43 | 0.51 | 0.62 | -0.22 | 0.00 | -0.20 | 0.07 | -0.04 | -0.03 | -0.22 | -0.07 | -8.79 | 0.09 |
| T400D | non-RRDR | 46.85 | 3 | 0 | 0.51 | 0.52 | 0.64 | 0.12 | 0.01 | -0.37 | 0.04 | -0.02 | 0.02 | -0.40 | -0.05 | -8.84 | 0.04 |
| T400E | non-RRDR | 46.85 | 3 | 0 | 0.49 | 0.51 | 0.64 | 0.12 | 0.01 | -0.37 | 0.04 | -0.02 | 0.02 | -0.40 | -0.05 | -8.84 | 0.04 |
| T400F | non-RRDR | 46.85 | 3 | 0 | 0.16 | 0.52 | 0.59 | -0.68 | 0.01 | -0.16 | 0.11 | -0.04 | -0.01 | -0.15 | -0.05 | -8.24 | 0.64 |
| T400G | non-RRDR | 46.85 | 3 | 0 | 0.62 | 0.53 | 0.65 | 0.07 | 0.01 | -0.29 | 0.08 | -0.04 | 0.00 | -0.31 | -0.07 | -8.89 | -0.01 |
| T400H | non-RRDR | 46.85 | 3 | 0 | 0.63 | 0.52 | 0.66 | 0.08 | 0.01 | -0.29 | 0.06 | -0.03 | 0.00 | -0.31 | -0.07 | -8.82 | 0.06 |
| T400I | non-RRDR | 46.85 | 3 | 0 | -0.52 | -0.52 | 0.68 | -0.66 | -0.01 | -0.04 | -0.04 | 0.02 | -0.04 | 0.16 | -0.07 | -8.86 | 0.02 |
| T400K | non-RRDR | 46.85 | 3 | 0 | 0.63 | 0.52 | 0.66 | 0.08 | 0.01 | -0.29 | 0.06 | -0.03 | 0.00 | -0.31 | -0.07 | -8.82 | 0.06 |
| T400L | non-RRDR | 46.85 | 3 | 0 | -0.21 | -0.07 | 0.56 | -0.55 | -0.01 | -0.07 | -0.02 | -0.03 | -0.03 | 0.05 | -0.04 | -8.86 | 0.02 |
| T400M | non-RRDR | 46.85 | 3 | 0 | -0.08 | -0.07 | 0.66 | -0.37 | -0.03 | -0.11 | 0.00 | -0.03 | -0.03 | -0.05 | -0.06 | -8.85 | 0.03 |
| T400N | non-RRDR | 46.85 | 3 | 0 | 0.56 | 0.51 | 0.65 | 0.11 | 0.01 | -0.35 | 0.05 | -0.02 | 0.01 | -0.36 | -0.05 | -8.86 | 0.02 |
| T400P | non-RRDR | 46.85 | 3 | 0 | 0.64 | 0.54 | 0.66 | 0.02 | 0.00 | -0.27 | 0.06 | -0.03 | 0.00 | -0.27 | -0.08 | -8.9 | -0.02 |
| T400Q | non-RRDR | 46.85 | 3 | 0 | 0.56 | 0.51 | 0.65 | 0.09 | 0.01 | -0.36 | 0.06 | -0.02 | 0.01 | -0.34 | -0.05 | -8.82 | 0.06 |
| T400R | non-RRDR | 46.85 | 3 | 0 | 0.65 | 0.53 | 0.66 | 0.06 | 0.01 | -0.28 | 0.06 | -0.03 | 0.00 | -0.29 | -0.07 | -8.91 | -0.03 |
| T400S | non-RRDR | 46.85 | 3 | 0 | 0.35 | 0.27 | 0.60 | 0.05 | -0.01 | -0.22 | 0.05 | -0.03 | -0.02 | -0.27 | -0.07 | -8.87 | 0.01 |
| T400T | non-RRDR | 46.85 | 3 | 0 | -0.19 | -0.43 | 0.27 | 0.08 | -0.06 | -0.05 | -0.03 | 0.02 | -0.02 | 0.09 | -0.07 | -8.87 | 0.01 |
| T400V | non-RRDR | 46.85 | 3 | 0 | -0.23 | -0.33 | 0.66 | -0.36 | 0.00 | -0.07 | -0.01 | -0.01 | -0.02 | -0.01 | -0.08 | -8.84 | 0.04 |
| T400W | non-RRDR | 46.85 | 3 | 0 | 0.48 | 0.60 | 0.67 | -0.32 | -0.02 | -0.20 | 0.12 | -0.05 | -0.03 | -0.23 | -0.07 | -8.83 | 0.05 |
| T400Y | non-RRDR | 46.85 | 3 | 0 | 0.23 | 0.52 | 0.65 | -0.66 | 0.01 | -0.16 | 0.13 | -0.04 | -0.01 | -0.15 | -0.06 | -8.84 | 0.04 |
| G475A | non-RRDR | 25.05 | 4 | 1 | 1.16 | 0.59 | 0.87 | -0.26 | 0.10 | -0.20 | 0.05 | -0.14 | 0.01 | 0.44 | -0.28 | -8.89 | -0.01 |
| G475C | non-RRDR | 25.05 | 4 | 1 | 1.40 | 0.73 | 0.90 | -0.40 | 0.10 | -0.15 | 0.08 | -0.14 | 0.03 | 0.41 | -0.17 | -8.82 | 0.06 |
| G475D | non-RRDR | 25.05 | 4 | 1 | 1.37 | 0.63 | 0.80 | 0.13 | 0.05 | -0.26 | -0.01 | -0.10 | 0.04 | 0.45 | -0.36 | -8.86 | 0.02 |
| G475E | non-RRDR | 25.05 | 4 | 1 | 1.45 | 0.68 | 0.82 | 0.09 | 0.05 | -0.21 | 0.01 | -0.11 | 0.05 | 0.45 | -0.36 | -8.84 | 0.04 |
| G475F | non-RRDR | 25.05 | 4 | 1 | 1.14 | 0.73 | 0.94 | -0.73 | 0.07 | -0.14 | 0.10 | -0.09 | 0.02 | 0.36 | -0.12 | -8.84 | 0.04 |
| G475G | non-RRDR | 25.05 | 4 | 1 | -0.31 | -0.50 | 0.44 | 0.16 | 0.02 | -0.13 | 0.02 | 0.02 | -0.01 | -0.03 | -0.30 | -8.79 | 0.09 |
| G475H | non-RRDR | 25.05 | 4 | 1 | 1.40 | 0.70 | 0.81 | -0.09 | 0.07 | -0.18 | 0.03 | -0.14 | 0.03 | 0.45 | -0.28 | -8.93 | -0.05 |
| G475I | non-RRDR | 25.05 | 4 | 1 | 1.18 | 0.64 | 0.96 | -0.70 | 0.08 | -0.13 | 0.12 | -0.08 | 0.02 | 0.35 | -0.09 | -8.87 | 0.01 |
| G475K | non-RRDR | 25.05 | 4 | 1 | 1.36 | 0.62 | 0.83 | -0.07 | 0.08 | -0.19 | 0.04 | -0.14 | 0.02 | 0.45 | -0.28 | -8.84 | 0.04 |
| G475L | non-RRDR | 25.05 | 4 | 1 | 1.21 | 0.74 | 0.97 | -0.71 | 0.08 | -0.14 | 0.11 | -0.09 | 0.02 | 0.35 | -0.11 | -8.88 | 0 |
| G475M | non-RRDR | 25.05 | 4 | 1 | 1.25 | 0.74 | 0.97 | -0.67 | 0.10 | -0.14 | 0.11 | -0.10 | 0.02 | 0.37 | -0.14 | -8.84 | 0.04 |
| G475N | non-RRDR | 25.05 | 4 | 1 | 1.39 | 0.61 | 0.82 | 0.03 | 0.07 | -0.20 | 0.02 | -0.13 | 0.03 | 0.47 | -0.33 | -8.85 | 0.03 |
| G475P | non-RRDR | 25.05 | 4 | 1 | 1.40 | 0.70 | 0.82 | -0.14 | 0.07 | -0.17 | 0.04 | -0.14 | 0.03 | 0.45 | -0.27 | -8.82 | 0.06 |
| G475Q | non-RRDR | 25.05 | 4 | 1 | 1.39 | 0.62 | 0.83 | 0.03 | 0.06 | -0.20 | 0.03 | -0.13 | 0.04 | 0.44 | -0.33 | -8.8 | 0.08 |
| G475R | non-RRDR | 25.05 | 4 | 1 | 1.41 | 0.71 | 0.81 | -0.10 | 0.06 | -0.18 | 0.03 | -0.15 | 0.03 | 0.46 | -0.28 | -8.87 | 0.01 |
| G475S | non-RRDR | 25.05 | 4 | 1 | 1.34 | 0.60 | 0.86 | -0.09 | 0.10 | -0.20 | 0.03 | -0.15 | 0.02 | 0.46 | -0.29 | -8.85 | 0.03 |
| G475T | non-RRDR | 25.05 | 4 | 1 | 1.23 | 0.63 | 0.87 | -0.32 | 0.09 | -0.18 | 0.06 | -0.13 | 0.02 | 0.42 | -0.24 | -8.86 | 0.02 |
| G475V | non-RRDR | 25.05 | 4 | 1 | 1.40 | 0.74 | 0.94 | -0.51 | 0.09 | -0.13 | 0.12 | -0.12 | 0.03 | 0.36 | -0.11 | -8.83 | 0.05 |
| G475W | non-RRDR | 25.05 | 4 | 1 | 1.32 | 0.72 | 0.87 | -0.41 | 0.09 | -0.15 | 0.09 | -0.14 | 0.02 | 0.39 | -0.18 | -8.81 | 0.07 |
| G475Y | non-RRDR | 25.05 | 4 | 1 | 1.10 | 0.73 | 0.94 | -0.79 | 0.08 | -0.14 | 0.11 | -0.09 | 0.02 | 0.36 | -0.12 | -8.93 | -0.05 |
| P589A | non-RRDR | 27.77 | 4 | 1 | 1.51 | 0.59 | 0.76 | -0.13 | 0.04 | -0.18 | 0.06 | -0.10 | 0.07 | 0.52 | -0.12 | -8.86 | 0.02 |
| P589C | non-RRDR | 27.77 | 4 | 1 | 1.38 | 0.62 | 0.82 | -0.40 | 0.05 | -0.15 | 0.08 | -0.07 | 0.07 | 0.46 | -0.10 | -8.85 | 0.03 |
| P589D | non-RRDR | 27.77 | 4 | 1 | 1.61 | 0.61 | 0.71 | 0.13 | -0.01 | -0.21 | -0.03 | -0.07 | 0.06 | 0.51 | -0.10 | -8.87 | 0.01 |
| P589E | non-RRDR | 27.77 | 4 | 1 | 1.66 | 0.60 | 0.72 | 0.18 | 0.00 | -0.21 | -0.03 | -0.07 | 0.06 | 0.52 | -0.10 | -8.88 | 0 |
| P589F | non-RRDR | 27.77 | 4 | 1 | 0.97 | 0.51 | 0.85 | -0.80 | 0.04 | -0.10 | 0.14 | -0.04 | 0.04 | 0.42 | -0.08 | -8.89 | -0.01 |
| P589G | non-RRDR | 27.77 | 4 | 1 | 1.60 | 0.63 | 0.77 | -0.04 | 0.03 | -0.19 | 0.04 | -0.09 | 0.07 | 0.49 | -0.11 | -8.79 | 0.09 |
| P589H | non-RRDR | 27.77 | 4 | 1 | 1.59 | 0.64 | 0.77 | -0.05 | 0.03 | -0.19 | 0.04 | -0.09 | 0.07 | 0.49 | -0.11 | -8.86 | 0.02 |
| P589I | non-RRDR | 27.77 | 4 | 1 | 1.08 | 0.61 | 0.86 | -0.75 | 0.04 | -0.13 | 0.13 | -0.05 | 0.06 | 0.41 | -0.09 | -8.88 | 0 |
| P589K | non-RRDR | 27.77 | 4 | 1 | 1.54 | 0.59 | 0.74 | -0.04 | 0.03 | -0.19 | 0.04 | -0.09 | 0.07 | 0.51 | -0.12 | -8.83 | 0.05 |
| P589L | non-RRDR | 27.77 | 4 | 1 | 1.11 | 0.61 | 0.86 | -0.73 | 0.04 | -0.13 | 0.13 | -0.05 | 0.06 | 0.41 | -0.08 | -8.91 | -0.03 |
| P589M | non-RRDR | 27.77 | 4 | 1 | 1.18 | 0.62 | 0.83 | -0.64 | 0.05 | -0.12 | 0.13 | -0.07 | 0.06 | 0.42 | -0.11 | -8.91 | -0.03 |
| P589N | non-RRDR | 27.77 | 4 | 1 | 1.67 | 0.62 | 0.74 | 0.11 | 0.00 | -0.19 | 0.00 | -0.09 | 0.07 | 0.50 | -0.11 | -8.85 | 0.03 |
| P589P | non-RRDR | 27.77 | 4 | 1 | -0.20 | -0.51 | 0.40 | 0.10 | -0.02 | -0.10 | -0.01 | 0.01 | -0.01 | 0.01 | -0.08 | -8.8 | 0.08 |
| P589Q | non-RRDR | 27.77 | 4 | 1 | 1.64 | 0.59 | 0.73 | 0.12 | 0.01 | -0.19 | 0.00 | -0.09 | 0.07 | 0.52 | -0.11 | -8.86 | 0.02 |
| P589R | non-RRDR | 27.77 | 4 | 1 | 1.57 | 0.64 | 0.77 | -0.08 | 0.03 | -0.19 | 0.04 | -0.09 | 0.07 | 0.50 | -0.11 | -8.88 | 0 |
| P589S | non-RRDR | 27.77 | 4 | 1 | 1.52 | 0.59 | 0.75 | -0.07 | 0.03 | -0.19 | 0.04 | -0.09 | 0.07 | 0.52 | -0.12 | -8.8 | 0.08 |
| P589T | non-RRDR | 27.77 | 4 | 1 | 1.45 | 0.59 | 0.76 | -0.19 | 0.04 | -0.18 | 0.06 | -0.10 | 0.07 | 0.51 | -0.11 | -8.85 | 0.03 |
| P589V | non-RRDR | 27.77 | 4 | 1 | 1.27 | 0.63 | 0.84 | -0.59 | 0.06 | -0.12 | 0.15 | -0.09 | 0.08 | 0.44 | -0.13 | -8.88 | 0 |
| P589W | non-RRDR | 27.77 | 4 | 1 | 1.20 | 0.49 | 0.83 | -0.50 | 0.05 | -0.14 | 0.12 | -0.06 | 0.06 | 0.45 | -0.10 | -8.85 | 0.03 |
| P589Y | non-RRDR | 27.77 | 4 | 1 | 1.04 | 0.61 | 0.85 | -0.79 | 0.03 | -0.13 | 0.12 | -0.04 | 0.05 | 0.42 | -0.08 | -8.81 | 0.07 |
| E748A | non-RRDR | 22.14 | 2 | 0 | 0.57 | 0.51 | 0.65 | -0.42 | 0.00 | -0.27 | 0.09 | -0.02 | 0.08 | -0.12 | 0.07 | -8.89 | -0.01 |
| E748C | non-RRDR | 22.14 | 2 | 0 | 0.63 | 0.51 | 0.71 | -0.47 | 0.00 | -0.20 | 0.10 | -0.03 | 0.06 | -0.11 | 0.05 | -8.87 | 0.01 |
| E748D | non-RRDR | 22.14 | 2 | 0 | 0.53 | -0.10 | 0.83 | 0.02 | -0.03 | -0.21 | 0.00 | -0.03 | 0.03 | -0.09 | 0.11 | -8.86 | 0.02 |
| E748E | non-RRDR | 22.14 | 2 | 0 | -0.27 | -0.47 | 0.23 | 0.02 | -0.05 | -0.04 | -0.04 | -0.01 | -0.07 | 0.03 | 0.14 | -8.87 | 0.01 |
| E748F | non-RRDR | 22.14 | 2 | 0 | 0.60 | 0.52 | 0.71 | -0.59 | -0.01 | -0.18 | 0.11 | -0.01 | 0.08 | -0.09 | 0.07 | -8.87 | 0.01 |
| E748G | non-RRDR | 22.14 | 2 | 0 | 0.74 | 0.55 | 0.71 | -0.21 | -0.01 | -0.29 | 0.04 | -0.02 | 0.04 | -0.15 | 0.08 | -8.93 | -0.05 |
| E748H | non-RRDR | 22.14 | 2 | 0 | 0.68 | 0.50 | 0.66 | -0.22 | 0.00 | -0.29 | 0.05 | -0.02 | 0.05 | -0.12 | 0.08 | -8.93 | -0.05 |
| E748I | non-RRDR | 22.14 | 2 | 0 | 0.56 | 0.53 | 0.72 | -0.69 | 0.00 | -0.18 | 0.12 | 0.00 | 0.10 | -0.09 | 0.06 | -8.8 | 0.08 |
| E748K | non-RRDR | 22.14 | 2 | 0 | 0.28 | -0.49 | 1.03 | -0.17 | -0.01 | -0.16 | 0.00 | -0.02 | 0.00 | -0.04 | 0.13 | -8.86 | 0.02 |
| E748L | non-RRDR | 22.14 | 2 | 0 | 0.56 | 0.57 | 0.74 | -0.70 | -0.01 | -0.20 | 0.12 | -0.01 | 0.08 | -0.09 | 0.06 | -8.83 | 0.05 |
| E748M | non-RRDR | 22.14 | 2 | 0 | 0.58 | 0.55 | 0.77 | -0.70 | -0.01 | -0.22 | 0.12 | 0.00 | 0.09 | -0.09 | 0.07 | -8.83 | 0.05 |
| E748N | non-RRDR | 22.14 | 2 | 0 | 0.67 | 0.50 | 0.67 | -0.26 | 0.02 | -0.30 | 0.04 | -0.02 | 0.05 | -0.11 | 0.08 | -8.85 | 0.03 |
| E748P | non-RRDR | 22.14 | 2 | 0 | 0.70 | 0.57 | 0.66 | -0.35 | -0.01 | -0.26 | 0.07 | -0.02 | 0.07 | -0.12 | 0.07 | -8.85 | 0.03 |
| E748Q | non-RRDR | 22.14 | 2 | 0 | 0.37 | -0.08 | 0.79 | -0.15 | -0.07 | -0.19 | -0.02 | -0.02 | 0.05 | -0.06 | 0.13 | -8.87 | 0.01 |
| E748R | non-RRDR | 22.14 | 2 | 0 | 0.49 | -0.08 | 0.91 | -0.16 | -0.06 | -0.20 | 0.01 | -0.02 | 0.04 | -0.08 | 0.11 | -8.88 | 0 |
| E748S | non-RRDR | 22.14 | 2 | 0 | 0.59 | 0.36 | 0.68 | -0.22 | 0.00 | -0.27 | 0.05 | -0.03 | 0.05 | -0.10 | 0.07 | -8.85 | 0.03 |
| E748T | non-RRDR | 22.14 | 2 | 0 | 0.56 | 0.51 | 0.66 | -0.43 | 0.00 | -0.26 | 0.09 | -0.02 | 0.08 | -0.13 | 0.07 | -8.86 | 0.02 |
| E748V | non-RRDR | 22.14 | 2 | 0 | 0.54 | 0.56 | 0.74 | -0.71 | -0.02 | -0.22 | 0.12 | 0.00 | 0.09 | -0.09 | 0.06 | -8.85 | 0.03 |
| E748W | non-RRDR | 22.14 | 2 | 0 | 0.61 | 0.57 | 0.75 | -0.62 | -0.02 | -0.21 | 0.11 | -0.01 | 0.08 | -0.10 | 0.06 | -8.85 | 0.03 |
| E748Y | non-RRDR | 22.14 | 2 | 0 | 0.59 | 0.55 | 0.76 | -0.64 | -0.02 | -0.21 | 0.12 | -0.01 | 0.08 | -0.11 | 0.07 | -8.86 | 0.02 |
| A760A | non-RRDR | 39.22 | 1 | 0 | -0.18 | -0.30 | 0.30 | 0.10 | 0.01 | -0.10 | 0.01 | 0.01 | -0.01 | 0.04 | -0.22 | -8.82 | 0.06 |
| A760C | non-RRDR | 39.22 | 1 | 0 | 0.76 | 0.71 | 0.54 | -0.27 | -0.01 | -0.19 | 0.03 | 0.05 | 0.06 | 0.02 | -0.17 | -8.86 | 0.02 |
| A760D | non-RRDR | 39.22 | 1 | 0 | 0.61 | 0.51 | 0.25 | 0.23 | 0.02 | -0.24 | 0.01 | 0.02 | 0.04 | -0.04 | -0.19 | -8.87 | 0.01 |
| A760E | non-RRDR | 39.22 | 1 | 0 | 0.58 | 0.59 | 0.14 | 0.23 | 0.02 | -0.24 | 0.03 | 0.01 | 0.03 | -0.04 | -0.19 | -8.89 | -0.01 |
| A760F | non-RRDR | 39.22 | 1 | 0 | 0.44 | 0.65 | 0.74 | -0.75 | 0.00 | -0.16 | 0.07 | 0.06 | 0.06 | -0.09 | -0.13 | -8.83 | 0.05 |
| A760G | non-RRDR | 39.22 | 1 | 0 | 0.88 | 0.61 | 0.39 | 0.09 | 0.02 | -0.21 | 0.04 | 0.05 | 0.09 | 0.00 | -0.19 | -8.86 | 0.02 |
| A760H | non-RRDR | 39.22 | 1 | 0 | 0.75 | 0.51 | 0.46 | 0.04 | 0.02 | -0.22 | 0.01 | 0.05 | 0.05 | 0.00 | -0.17 | -8.81 | 0.07 |
| A760I | non-RRDR | 39.22 | 1 | 0 | 0.46 | 0.63 | 0.62 | -0.63 | 0.00 | -0.16 | 0.07 | 0.06 | 0.08 | -0.03 | -0.17 | -8.83 | 0.05 |
| A760K | non-RRDR | 39.22 | 1 | 0 | 0.69 | 0.52 | 0.29 | 0.10 | 0.01 | -0.20 | 0.02 | 0.04 | 0.07 | 0.01 | -0.18 | -8.90 | -0.02 |
| A760L | non-RRDR | 39.22 | 1 | 0 | 0.53 | 0.68 | 0.62 | -0.61 | -0.01 | -0.16 | 0.06 | 0.06 | 0.08 | -0.03 | -0.16 | -8.83 | 0.05 |
| A760M | non-RRDR | 39.22 | 1 | 0 | 0.76 | 0.70 | 0.49 | -0.41 | 0.00 | -0.09 | 0.07 | 0.07 | 0.11 | 0.01 | -0.18 | -8.88 | 0 |
| A760N | non-RRDR | 39.22 | 1 | 0 | 0.88 | 0.60 | 0.43 | 0.15 | 0.01 | -0.24 | 0.05 | 0.04 | 0.08 | -0.02 | -0.21 | -8.82 | 0.06 |
| A760P | non-RRDR | 39.22 | 1 | 0 | -0.58 | -0.36 | -0.61 | 0.29 | -0.01 | 0.04 | 0.08 | -0.03 | 0.01 | 0.16 | -0.14 | -8.83 | 0.05 |
| A760Q | non-RRDR | 39.22 | 1 | 0 | 0.73 | 0.61 | 0.27 | 0.16 | 0.01 | -0.23 | 0.04 | 0.02 | 0.06 | -0.02 | -0.19 | -8.87 | 0.01 |
| A760R | non-RRDR | 39.22 | 1 | 0 | 0.80 | 0.61 | 0.43 | 0.00 | 0.00 | -0.21 | 0.03 | 0.05 | 0.08 | -0.01 | -0.19 | -8.80 | 0.08 |
| A760S | non-RRDR | 39.22 | 1 | 0 | 0.18 | 0.36 | 0.06 | 0.07 | 0.03 | -0.14 | -0.03 | 0.00 | 0.03 | 0.03 | -0.22 | -8.83 | 0.05 |
| A760T | non-RRDR | 39.22 | 1 | 0 | 0.61 | 0.59 | 0.29 | -0.03 | 0.01 | -0.19 | 0.02 | 0.02 | 0.07 | 0.01 | -0.19 | -8.87 | 0.01 |
| A760V | non-RRDR | 39.22 | 1 | 0 | 0.73 | 0.65 | 0.47 | -0.38 | 0.01 | -0.10 | 0.07 | 0.07 | 0.12 | 0.01 | -0.18 | -8.79 | 0.09 |
| A760W | non-RRDR | 39.22 | 1 | 0 | 0.75 | 0.62 | 0.70 | -0.37 | -0.01 | -0.19 | 0.05 | 0.06 | 0.06 | -0.03 | -0.13 | -8.78 | 0.1 |
| A760Y | non-RRDR | 39.22 | 1 | 0 | 0.38 | 0.67 | 0.62 | -0.72 | -0.01 | -0.16 | 0.06 | 0.05 | 0.08 | -0.05 | -0.16 | -8.81 | 0.07 |
| E959A | non-RRDR | 47.21 | 2 | 0 | -0.07 | 0.27 | 0.35 | -0.26 | -0.06 | -0.11 | 0.01 | -0.02 | 0.00 | -0.34 | 0.08 | -8.8 | 0.08 |
| E959C | non-RRDR | 47.21 | 2 | 0 | 0.07 | 0.38 | 0.46 | -0.47 | -0.01 | -0.13 | 0.08 | -0.04 | 0.04 | -0.32 | 0.09 | -8.84 | 0.04 |
| E959D | non-RRDR | 47.21 | 2 | 0 | 0.00 | -0.03 | 0.19 | 0.06 | -0.10 | -0.09 | 0.00 | -0.01 | 0.01 | -0.13 | 0.11 | -8.84 | 0.04 |
| E959E | non-RRDR | 47.21 | 2 | 0 | -0.07 | -0.01 | 0.00 | 0.03 | -0.10 | -0.05 | -0.02 | 0.01 | 0.01 | -0.05 | 0.12 | -8.83 | 0.05 |
| E959F | non-RRDR | 47.21 | 2 | 0 | -0.08 | 0.35 | 0.35 | -0.61 | 0.00 | -0.11 | 0.06 | -0.01 | 0.03 | -0.24 | 0.12 | -8.88 | 0 |
| E959G | non-RRDR | 47.21 | 2 | 0 | -0.44 | -0.33 | 0.37 | -0.27 | -0.06 | -0.07 | -0.05 | 0.01 | 0.01 | -0.17 | 0.12 | -8.85 | 0.03 |
| E959H | non-RRDR | 47.21 | 2 | 0 | -0.10 | 0.28 | 0.31 | -0.25 | -0.07 | -0.12 | -0.01 | -0.01 | 0.00 | -0.31 | 0.08 | -8.85 | 0.03 |
| E959I | non-RRDR | 47.21 | 2 | 0 | -0.12 | 0.38 | 0.35 | -0.68 | 0.00 | -0.11 | 0.06 | -0.01 | 0.02 | -0.23 | 0.10 | -8.86 | 0.02 |
| E959K | non-RRDR | 47.21 | 2 | 0 | -0.19 | 0.30 | 0.13 | -0.24 | -0.05 | -0.12 | -0.01 | 0.00 | 0.00 | -0.26 | 0.07 | -8.87 | 0.01 |
| E959L | non-RRDR | 47.21 | 2 | 0 | -0.12 | 0.38 | 0.35 | -0.68 | 0.00 | -0.11 | 0.06 | -0.01 | 0.02 | -0.23 | 0.10 | -8.86 | 0.02 |
| E959M | non-RRDR | 47.21 | 2 | 0 | -0.15 | 0.38 | 0.32 | -0.67 | 0.00 | -0.11 | 0.03 | -0.01 | 0.01 | -0.22 | 0.10 | -8.85 | 0.03 |
| E959N | non-RRDR | 47.21 | 2 | 0 | -0.07 | 0.28 | 0.26 | -0.21 | -0.07 | -0.14 | 0.01 | 0.00 | 0.01 | -0.29 | 0.08 | -8.81 | 0.07 |
| E959P | non-RRDR | 47.21 | 2 | 0 | 0.05 | 0.41 | 0.32 | -0.27 | -0.03 | -0.13 | 0.02 | -0.02 | 0.02 | -0.36 | 0.09 | -8.91 | -0.03 |
| E959Q | non-RRDR | 47.21 | 2 | 0 | -0.12 | 0.29 | 0.16 | -0.20 | -0.05 | -0.14 | 0.00 | 0.01 | 0.01 | -0.27 | 0.08 | -8.78 | 0.1 |
| E959R | non-RRDR | 47.21 | 2 | 0 | -0.11 | 0.27 | 0.36 | -0.27 | -0.06 | -0.12 | -0.01 | -0.02 | 0.00 | -0.34 | 0.08 | -8.87 | 0.01 |
| E959S | non-RRDR | 47.21 | 2 | 0 | -0.16 | 0.28 | 0.22 | -0.27 | -0.06 | -0.12 | 0.00 | -0.01 | 0.00 | -0.28 | 0.08 | -8.87 | 0.01 |
| E959T | non-RRDR | 47.21 | 2 | 0 | -0.06 | 0.27 | 0.36 | -0.27 | -0.06 | -0.11 | 0.01 | -0.02 | 0.00 | -0.33 | 0.09 | -8.82 | 0.06 |
| E959V | non-RRDR | 47.21 | 2 | 0 | -0.15 | 0.38 | 0.31 | -0.67 | 0.00 | -0.10 | 0.03 | 0.00 | 0.01 | -0.22 | 0.10 | -8.84 | 0.04 |
| E959W | non-RRDR | 47.21 | 2 | 0 | 0.00 | 0.37 | 0.48 | -0.62 | -0.01 | -0.13 | 0.07 | -0.02 | 0.04 | -0.28 | 0.10 | -8.84 | 0.04 |
| E959Y | non-RRDR | 47.21 | 2 | 0 | -0.11 | 0.36 | 0.33 | -0.63 | 0.00 | -0.12 | 0.05 | -0.01 | 0.02 | -0.23 | 0.12 | -8.84 | 0.04 |
| D993A | non-RRDR | 52.76 | 1 | 0 | 0.35 | 0.43 | 0.37 | -0.26 | -0.01 | -0.09 | 0.07 | 0.07 | 0.00 | -0.18 | -0.07 | -8.85 | 0.03 |
| D993C | non-RRDR | 52.76 | 1 | 0 | 0.36 | 0.51 | 0.55 | -0.50 | -0.01 | -0.12 | 0.12 | 0.08 | 0.00 | -0.16 | -0.09 | -8.86 | 0.02 |
| D993D | non-RRDR | 52.76 | 1 | 0 | -0.10 | -0.29 | 0.16 | 0.03 | -0.05 | -0.04 | -0.03 | 0.04 | 0.01 | 0.05 | 0.02 | -8.84 | 0.04 |
| D993E | non-RRDR | 52.76 | 1 | 0 | -0.14 | -0.37 | 0.14 | 0.04 | -0.03 | -0.05 | -0.06 | 0.02 | 0.02 | 0.09 | 0.04 | -8.83 | 0.05 |
| D993F | non-RRDR | 52.76 | 1 | 0 | 0.21 | 0.50 | 0.48 | -0.64 | -0.02 | -0.09 | 0.10 | 0.09 | 0.00 | -0.15 | -0.06 | -8.78 | 0.1 |
| D993G | non-RRDR | 52.76 | 1 | 0 | 0.35 | 0.43 | 0.36 | -0.22 | -0.01 | -0.10 | 0.07 | 0.08 | 0.00 | -0.19 | -0.07 | -8.78 | 0.1 |
| D993H | non-RRDR | 52.76 | 1 | 0 | 0.36 | 0.43 | 0.37 | -0.22 | 0.00 | -0.09 | 0.07 | 0.08 | 0.00 | -0.19 | -0.07 | -8.83 | 0.05 |
| D993I | non-RRDR | 52.76 | 1 | 0 | 0.12 | 0.38 | 0.48 | -0.66 | 0.01 | -0.08 | 0.09 | 0.06 | 0.00 | -0.13 | -0.05 | -8.81 | 0.07 |
| D993K | non-RRDR | 52.76 | 1 | 0 | -0.02 | -0.07 | 0.57 | -0.24 | -0.07 | -0.06 | -0.03 | 0.04 | 0.01 | -0.16 | -0.01 | -8.86 | 0.02 |
| D993L | non-RRDR | 52.76 | 1 | 0 | 0.11 | 0.38 | 0.48 | -0.66 | 0.01 | -0.07 | 0.10 | 0.06 | 0.00 | -0.13 | -0.05 | -8.9 | -0.02 |
| D993M | non-RRDR | 52.76 | 1 | 0 | 0.03 | 0.39 | 0.39 | -0.67 | 0.02 | -0.07 | 0.07 | 0.05 | 0.00 | -0.10 | -0.03 | -8.86 | 0.02 |
| D993N | non-RRDR | 52.76 | 1 | 0 | 0.16 | 0.28 | 0.38 | -0.24 | -0.02 | -0.11 | 0.03 | 0.07 | 0.00 | -0.17 | -0.06 | -8.86 | 0.02 |
| D993P | non-RRDR | 52.76 | 1 | 0 | 0.39 | 0.42 | 0.45 | -0.26 | -0.01 | -0.10 | 0.09 | 0.08 | 0.01 | -0.20 | -0.08 | -8.81 | 0.07 |
| D993Q | non-RRDR | 52.76 | 1 | 0 | -0.03 | -0.07 | 0.58 | -0.22 | -0.08 | -0.07 | -0.04 | 0.05 | 0.01 | -0.17 | -0.02 | -8.89 | -0.01 |
| D993R | non-RRDR | 52.76 | 1 | 0 | 0.01 | -0.06 | 0.59 | -0.22 | -0.07 | -0.08 | -0.02 | 0.04 | 0.01 | -0.17 | -0.01 | -8.95 | -0.07 |
| D993S | non-RRDR | 52.76 | 1 | 0 | 0.31 | 0.44 | 0.33 | -0.25 | 0.00 | -0.08 | 0.05 | 0.07 | 0.00 | -0.18 | -0.07 | -8.84 | 0.04 |
| D993T | non-RRDR | 52.76 | 1 | 0 | 0.34 | 0.43 | 0.38 | -0.29 | 0.00 | -0.09 | 0.07 | 0.07 | 0.01 | -0.17 | -0.07 | -8.85 | 0.03 |
| D993V | non-RRDR | 52.76 | 1 | 0 | -0.01 | 0.25 | 0.48 | -0.65 | -0.01 | -0.06 | 0.06 | 0.03 | 0.00 | -0.08 | -0.03 | -8.82 | 0.06 |
| D993W | non-RRDR | 52.76 | 1 | 0 | 0.28 | 0.51 | 0.55 | -0.61 | -0.01 | -0.12 | 0.12 | 0.08 | 0.00 | -0.15 | -0.09 | -8.87 | 0.01 |
| D993Y | non-RRDR | 52.76 | 1 | 0 | 0.23 | 0.50 | 0.47 | -0.62 | -0.02 | -0.09 | 0.11 | 0.09 | 0.00 | -0.15 | -0.06 | -8.89 | -0.01 |
| A1075A | non-RRDR | 39.7 | 1 | 0 | -0.24 | -0.41 | 0.46 | 0.13 | 0.02 | -0.11 | 0.02 | -0.06 | -0.03 | -0.03 | -0.22 | -8.81 | 0.07 |
| A1075C | non-RRDR | 39.7 | 1 | 0 | 0.84 | 0.57 | 0.75 | -0.22 | 0.00 | -0.22 | 0.07 | 0.04 | -0.01 | -0.01 | -0.14 | -8.85 | 0.03 |
| A1075D | non-RRDR | 39.7 | 1 | 0 | 0.80 | 0.57 | 0.77 | 0.10 | 0.02 | -0.39 | 0.01 | 0.05 | -0.04 | -0.10 | -0.19 | -8.82 | 0.06 |
| A1075E | non-RRDR | 39.7 | 1 | 0 | 0.70 | 0.53 | 0.75 | 0.09 | 0.01 | -0.40 | 0.01 | 0.06 | -0.06 | -0.10 | -0.20 | -8.78 | 0.1 |
| A1075F | non-RRDR | 39.7 | 1 | 0 | 0.67 | 0.59 | 0.91 | -0.67 | 0.03 | -0.17 | 0.12 | 0.08 | -0.03 | -0.05 | -0.12 | -8.82 | 0.06 |
| A1075G | non-RRDR | 39.7 | 1 | 0 | 0.92 | 0.58 | 0.74 | 0.03 | 0.04 | -0.31 | 0.04 | 0.06 | -0.04 | -0.06 | -0.16 | -8.85 | 0.03 |
| A1075H | non-RRDR | 39.7 | 1 | 0 | 0.98 | 0.58 | 0.81 | -0.01 | 0.02 | -0.30 | 0.04 | 0.09 | -0.03 | -0.04 | -0.17 | -8.85 | 0.03 |
| A1075I | non-RRDR | 39.7 | 1 | 0 | 0.73 | 0.59 | 0.92 | -0.61 | 0.03 | -0.19 | 0.13 | 0.07 | -0.03 | -0.06 | -0.13 | -8.87 | 0.01 |
| A1075K | non-RRDR | 39.7 | 1 | 0 | 0.93 | 0.54 | 0.78 | 0.04 | 0.03 | -0.31 | 0.04 | 0.09 | -0.04 | -0.05 | -0.18 | -8.86 | 0.02 |
| A1075L | non-RRDR | 39.7 | 1 | 0 | 0.71 | 0.59 | 0.92 | -0.62 | 0.03 | -0.18 | 0.13 | 0.07 | -0.03 | -0.06 | -0.13 | -8.84 | 0.04 |
| A1075M | non-RRDR | 39.7 | 1 | 0 | 0.83 | 0.57 | 0.88 | -0.45 | 0.01 | -0.18 | 0.14 | 0.06 | -0.02 | -0.06 | -0.12 | -8.89 | -0.01 |
| A1075N | non-RRDR | 39.7 | 1 | 0 | 1.03 | 0.58 | 0.79 | 0.10 | 0.02 | -0.31 | 0.03 | 0.11 | -0.03 | -0.07 | -0.19 | -8.89 | -0.01 |
| A1075P | non-RRDR | 39.7 | 1 | 0 | 0.92 | 0.55 | 0.79 | -0.02 | 0.01 | -0.30 | 0.05 | 0.08 | -0.03 | -0.03 | -0.17 | -8.85 | 0.03 |
| A1075Q | non-RRDR | 39.7 | 1 | 0 | 0.94 | 0.54 | 0.77 | 0.10 | 0.02 | -0.32 | 0.04 | 0.10 | -0.04 | -0.07 | -0.19 | -8.81 | 0.07 |
| A1075R | non-RRDR | 39.7 | 1 | 0 | 0.91 | 0.54 | 0.79 | -0.03 | 0.01 | -0.30 | 0.05 | 0.08 | -0.03 | -0.03 | -0.18 | -8.88 | 0 |
| A1075S | non-RRDR | 39.7 | 1 | 0 | 0.79 | 0.50 | 0.66 | -0.02 | 0.03 | -0.28 | 0.04 | 0.06 | -0.02 | -0.01 | -0.17 | -8.83 | 0.05 |
| A1075T | non-RRDR | 39.7 | 1 | 0 | 0.96 | 0.57 | 0.76 | -0.03 | 0.02 | -0.28 | 0.06 | 0.06 | -0.01 | -0.03 | -0.16 | -8.92 | -0.04 |
| A1075V | non-RRDR | 39.7 | 1 | 0 | 0.84 | 0.57 | 0.86 | -0.44 | 0.03 | -0.17 | 0.14 | 0.05 | -0.03 | -0.07 | -0.11 | -8.78 | 0.1 |
| A1075W | non-RRDR | 39.7 | 1 | 0 | 0.91 | 0.59 | 0.88 | -0.37 | -0.04 | -0.19 | 0.09 | 0.07 | 0.00 | -0.02 | -0.10 | -8.90 | -0.02 |
| A1075Y | non-RRDR | 39.7 | 1 | 0 | 0.69 | 0.59 | 0.91 | -0.64 | 0.02 | -0.17 | 0.12 | 0.08 | -0.03 | -0.06 | -0.12 | -8.86 | 0.02 |
| V169A | RRDR | 11.99 | 2 | 1 | 2.16 | 0.54 | 0.62 | 0.56 | -0.03 | -0.18 | 0.00 | 0.02 | -0.05 | 0.52 | 0.15 | -8.89 | -0.01 |
| V169C | RRDR | 11.99 | 2 | 1 | 1.67 | 0.59 | 0.69 | 0.08 | -0.03 | -0.28 | -0.04 | 0.09 | -0.06 | 0.52 | 0.11 | -8.89 | -0.01 |
| V169D | RRDR | 11.99 | 2 | 1 | 2.62 | 0.81 | 0.70 | 0.88 | -0.06 | -0.27 | -0.01 | 0.04 | -0.10 | 0.49 | 0.15 | -8.82 | 0.06 |
| V169E | RRDR | 11.99 | 2 | 1 | 2.55 | 0.80 | 0.64 | 0.88 | -0.05 | -0.28 | -0.01 | 0.04 | -0.10 | 0.49 | 0.15 | -8.86 | 0.02 |
| V169F | RRDR | 11.99 | 2 | 1 | 1.21 | 0.62 | 0.74 | -0.62 | -0.04 | -0.20 | 0.12 | 0.11 | -0.06 | 0.45 | 0.08 | -8.82 | 0.06 |
| V169G | RRDR | 11.99 | 2 | 1 | 2.62 | 0.81 | 0.71 | 0.84 | -0.06 | -0.26 | -0.01 | 0.05 | -0.09 | 0.48 | 0.15 | -8.85 | 0.03 |
| V169H | RRDR | 11.99 | 2 | 1 | 2.60 | 0.82 | 0.71 | 0.80 | -0.06 | -0.26 | 0.00 | 0.05 | -0.07 | 0.48 | 0.15 | -8.88 | 0 |
| V169I | RRDR | 11.99 | 2 | 1 | -0.13 | -0.51 | 0.51 | -0.17 | -0.01 | -0.08 | 0.00 | -0.02 | 0.07 | 0.01 | 0.07 | -8.84 | 0.04 |
| V169K | RRDR | 11.99 | 2 | 1 | 2.45 | 0.69 | 0.67 | 0.84 | -0.05 | -0.26 | -0.03 | 0.06 | -0.09 | 0.48 | 0.15 | -8.81 | 0.07 |
| V169L | RRDR | 11.99 | 2 | 1 | 1.11 | 0.51 | 0.47 | -0.32 | 0.00 | -0.16 | 0.00 | 0.14 | -0.01 | 0.43 | 0.05 | -8.81 | 0.07 |
| V169M | RRDR | 11.99 | 2 | 1 | 1.40 | 0.51 | 0.58 | -0.17 | -0.01 | -0.21 | 0.03 | 0.16 | -0.05 | 0.51 | 0.05 | -8.9 | -0.02 |
| V169N | RRDR | 11.99 | 2 | 1 | 2.59 | 0.81 | 0.70 | 0.85 | -0.06 | -0.28 | -0.01 | 0.05 | -0.09 | 0.49 | 0.15 | -8.87 | 0.01 |
| V169P | RRDR | 11.99 | 2 | 1 | 2.29 | 0.66 | 0.67 | 0.64 | -0.03 | -0.22 | -0.02 | 0.04 | -0.06 | 0.46 | 0.14 | -8.82 | 0.06 |
| V169Q | RRDR | 11.99 | 2 | 1 | 2.42 | 0.69 | 0.67 | 0.83 | -0.05 | -0.27 | -0.03 | 0.06 | -0.10 | 0.48 | 0.14 | -8.87 | 0.01 |
| V169R | RRDR | 11.99 | 2 | 1 | 2.49 | 0.69 | 0.74 | 0.78 | -0.06 | -0.24 | -0.02 | 0.06 | -0.06 | 0.47 | 0.15 | -8.89 | -0.01 |
| V169S | RRDR | 11.99 | 2 | 1 | 2.45 | 0.70 | 0.64 | 0.78 | -0.04 | -0.24 | -0.02 | 0.08 | -0.07 | 0.47 | 0.15 | -8.84 | 0.04 |
| V169T | RRDR | 11.99 | 2 | 1 | 1.99 | 0.55 | 0.62 | 0.40 | -0.01 | -0.24 | 0.01 | 0.05 | -0.04 | 0.51 | 0.14 | -8.84 | 0.04 |
| V169W | RRDR | 11.99 | 2 | 1 | 1.68 | 0.59 | 0.74 | 0.01 | -0.01 | -0.24 | 0.00 | 0.12 | -0.08 | 0.48 | 0.08 | -8.88 | 0 |
| V169Y | RRDR | 11.99 | 2 | 1 | 1.21 | 0.58 | 0.73 | -0.57 | -0.03 | -0.20 | 0.10 | 0.14 | -0.06 | 0.44 | 0.07 | -8.83 | 0.05 |
| L430A | RRDR | 9.16 | 4 | 1 | 1.89 | 0.47 | 0.64 | 0.81 | -0.07 | -0.33 | -0.07 | 0.02 | -0.12 | 0.38 | 0.16 | -8.77 | 0.11 |
| L430C | RRDR | 9.16 | 4 | 1 | 1.27 | 0.56 | 0.61 | 0.09 | -0.05 | -0.32 | -0.06 | -0.03 | -0.06 | 0.37 | 0.16 | -8.82 | 0.06 |
| L430D | RRDR | 9.16 | 4 | 1 | 2.37 | 0.68 | 0.76 | 0.89 | -0.06 | -0.32 | -0.04 | 0.00 | -0.12 | 0.43 | 0.14 | -8.77 | 0.11 |
| L430E | RRDR | 9.16 | 4 | 1 | 2.39 | 0.69 | 0.77 | 0.90 | -0.07 | -0.31 | -0.03 | 0.00 | -0.12 | 0.42 | 0.14 | -8.75 | 0.13 |
| L430F | RRDR | 9.16 | 4 | 1 | 0.94 | 0.49 | 0.75 | -0.47 | -0.03 | -0.23 | 0.04 | -0.08 | -0.06 | 0.38 | 0.16 | -8.99 | -0.11 |
| L430G | RRDR | 9.16 | 4 | 1 | 2.32 | 0.69 | 0.77 | 0.84 | -0.07 | -0.31 | -0.04 | -0.01 | -0.12 | 0.43 | 0.14 | -8.72 | 0.16 |
| L430H | RRDR | 9.16 | 4 | 1 | 2.14 | 0.51 | 0.82 | 0.84 | -0.08 | -0.32 | -0.07 | 0.03 | -0.12 | 0.39 | 0.14 | -8.93 | -0.05 |
| L430I | RRDR | 9.16 | 4 | 1 | 0.91 | 0.28 | 0.45 | -0.11 | -0.01 | -0.18 | 0.00 | -0.05 | 0.02 | 0.32 | 0.20 | -8.93 | -0.05 |
| L430K | RRDR | 9.16 | 4 | 1 | 2.04 | 0.50 | 0.72 | 0.85 | -0.07 | -0.32 | -0.08 | 0.02 | -0.11 | 0.39 | 0.15 | -8.84 | 0.04 |
| L430M | RRDR | 9.16 | 4 | 1 | 0.97 | -0.02 | 0.66 | -0.04 | 0.02 | -0.16 | 0.03 | -0.04 | 0.05 | 0.27 | 0.20 | -8.84 | 0.04 |
| L430N | RRDR | 9.16 | 4 | 1 | 2.37 | 0.69 | 0.78 | 0.87 | -0.07 | -0.31 | -0.03 | 0.00 | -0.12 | 0.42 | 0.14 | -8.85 | 0.03 |
| L430P | RRDR | 9.16 | 4 | 1 | 2.31 | 0.69 | 0.78 | 0.83 | -0.07 | -0.31 | -0.03 | -0.01 | -0.12 | 0.41 | 0.14 | -8.86 | 0.02 |
| L430Q | RRDR | 9.16 | 4 | 1 | 2.06 | 0.50 | 0.72 | 0.88 | -0.07 | -0.33 | -0.07 | 0.03 | -0.11 | 0.39 | 0.14 | -8.23 | 0.65 |
| L430R | RRDR | 9.16 | 4 | 1 | 2.02 | 0.50 | 0.72 | 0.84 | -0.08 | -0.32 | -0.08 | 0.03 | -0.11 | 0.38 | 0.14 | -8.85 | 0.03 |
| L430S | RRDR | 9.16 | 4 | 1 | 2.00 | 0.50 | 0.72 | 0.83 | -0.08 | -0.32 | -0.08 | 0.02 | -0.11 | 0.38 | 0.14 | -8.81 | 0.07 |
| L430T | RRDR | 9.16 | 4 | 1 | 1.95 | 0.49 | 0.63 | 0.79 | -0.06 | -0.31 | -0.05 | 0.01 | -0.09 | 0.37 | 0.16 | -8.84 | 0.04 |
| L430V | RRDR | 9.16 | 4 | 1 | 1.14 | 0.44 | 0.62 | -0.11 | -0.02 | -0.25 | 0.01 | -0.07 | -0.05 | 0.41 | 0.16 | -8.8 | 0.08 |
| L430W | RRDR | 9.16 | 4 | 1 | 1.22 | 0.55 | 0.65 | 0.11 | -0.06 | -0.34 | -0.07 | -0.04 | -0.08 | 0.35 | 0.16 | -8.92 | -0.04 |
| L430Y | RRDR | 9.16 | 4 | 1 | 0.99 | 0.59 | 0.66 | -0.36 | -0.05 | -0.27 | 0.05 | -0.09 | -0.06 | 0.33 | 0.20 | -8.87 | 0.01 |
| G485A | RRDR | 11.51 | 1 | 0 | 0.86 | 0.50 | 0.87 | -0.12 | 0.01 | -0.25 | 0.05 | 0.11 | -0.13 | 0.00 | -0.17 | -8.94 | -0.06 |
| G485C | RRDR | 11.51 | 1 | 0 | 1.05 | 0.56 | 1.02 | -0.34 | -0.02 | -0.18 | 0.12 | 0.13 | -0.12 | -0.03 | -0.09 | -8.91 | -0.03 |
| G485D | RRDR | 11.51 | 1 | 0 | 0.63 | 0.51 | 0.86 | 0.06 | -0.01 | -0.37 | 0.04 | 0.07 | -0.19 | -0.10 | -0.24 | -8.75 | 0.13 |
| G485E | RRDR | 11.51 | 1 | 0 | 0.81 | 0.52 | 0.91 | 0.04 | 0.00 | -0.29 | 0.05 | 0.08 | -0.17 | -0.09 | -0.23 | -9.25 | -0.37 |
| G485F | RRDR | 11.51 | 1 | 0 | 0.94 | 0.58 | 1.04 | -0.56 | -0.01 | -0.15 | 0.12 | 0.12 | -0.08 | -0.04 | -0.07 | -9.41 | -0.53 |
| G485H | RRDR | 11.51 | 1 | 0 | 0.89 | 0.53 | 0.93 | -0.03 | -0.01 | -0.26 | 0.06 | 0.11 | -0.16 | -0.07 | -0.21 | -9.17 | -0.29 |
| G485I | RRDR | 11.51 | 1 | 0 | 1.04 | 0.62 | 1.04 | -0.59 | 0.01 | -0.14 | 0.14 | 0.13 | -0.09 | -0.03 | -0.05 | -8.9 | -0.02 |
| G485K | RRDR | 11.51 | 1 | 0 | 0.90 | 0.52 | 0.93 | -0.01 | -0.01 | -0.27 | 0.07 | 0.11 | -0.16 | -0.06 | -0.22 | -9.22 | -0.34 |
| G485L | RRDR | 11.51 | 1 | 0 | 1.05 | 0.62 | 1.04 | -0.59 | 0.01 | -0.14 | 0.14 | 0.13 | -0.09 | -0.03 | -0.05 | -9.17 | -0.29 |
| G485M | RRDR | 11.51 | 1 | 0 | 1.02 | 0.58 | 1.06 | -0.55 | 0.00 | -0.15 | 0.13 | 0.13 | -0.08 | -0.03 | -0.06 | -9.25 | -0.37 |
| G485N | RRDR | 11.51 | 1 | 0 | 0.73 | 0.57 | 0.82 | -0.02 | 0.00 | -0.31 | 0.04 | 0.07 | -0.18 | -0.06 | -0.21 | -9 | -0.12 |
| G485P | RRDR | 11.51 | 1 | 0 | 0.91 | 0.53 | 0.93 | -0.09 | -0.01 | -0.25 | 0.08 | 0.12 | -0.15 | -0.06 | -0.19 | -9.02 | -0.14 |
| G485Q | RRDR | 11.51 | 1 | 0 | 0.87 | 0.53 | 0.94 | 0.00 | 0.00 | -0.28 | 0.05 | 0.10 | -0.17 | -0.07 | -0.22 | -9.24 | -0.36 |
| G485R | RRDR | 11.51 | 1 | 0 | 0.89 | 0.53 | 0.93 | -0.05 | -0.01 | -0.26 | 0.07 | 0.11 | -0.16 | -0.07 | -0.21 | -8.94 | -0.06 |
| G485S | RRDR | 11.51 | 1 | 0 | 0.82 | 0.57 | 0.82 | -0.05 | -0.01 | -0.29 | 0.06 | 0.10 | -0.16 | -0.04 | -0.19 | -8.97 | -0.09 |
| G485T | RRDR | 11.51 | 1 | 0 | 0.93 | 0.55 | 0.93 | -0.16 | -0.01 | -0.24 | 0.09 | 0.13 | -0.12 | -0.02 | -0.18 | -9.04 | -0.16 |
| G485V | RRDR | 11.51 | 1 | 0 | 1.15 | 0.57 | 1.06 | -0.38 | -0.01 | -0.14 | 0.13 | 0.13 | -0.09 | -0.05 | -0.06 | -8.94 | -0.06 |
| G485W | RRDR | 11.51 | 1 | 0 | 0.96 | 0.56 | 0.97 | -0.37 | -0.02 | -0.17 | 0.11 | 0.13 | -0.11 | -0.03 | -0.11 | -9.23 | -0.35 |
| G485Y | RRDR | 11.51 | 1 | 0 | 0.92 | 0.58 | 1.05 | -0.64 | 0.01 | -0.15 | 0.13 | 0.12 | -0.08 | -0.04 | -0.06 | -9.45 | -0.57 |
| Q608A | RRDR | 13.82 | 4 | 1 | 0.84 | 0.56 | 0.67 | -0.27 | -0.04 | -0.27 | 0.05 | -0.08 | -0.01 | 0.12 | 0.12 | -8.05 | 0.83 |
| Q608C | RRDR | 13.82 | 4 | 1 | 0.56 | 0.49 | 0.69 | -0.57 | -0.06 | -0.20 | 0.10 | -0.06 | 0.01 | 0.09 | 0.08 | -8.75 | 0.13 |
| Q608D | RRDR | 13.82 | 4 | 1 | 0.94 | 0.53 | 0.64 | -0.01 | -0.02 | -0.33 | 0.04 | -0.06 | -0.02 | 0.06 | 0.10 | -8.78 | 0.1 |
| Q608E | RRDR | 13.82 | 4 | 1 | 0.79 | 0.27 | 0.67 | -0.04 | -0.03 | -0.28 | 0.01 | -0.05 | 0.00 | 0.15 | 0.09 | -8.73 | 0.15 |
| Q608F | RRDR | 13.82 | 4 | 1 | 0.53 | 0.50 | 0.75 | -0.78 | -0.04 | -0.14 | 0.14 | -0.05 | 0.03 | 0.04 | 0.08 | -8.72 | 0.16 |
| Q608G | RRDR | 13.82 | 4 | 1 | 0.90 | 0.56 | 0.69 | -0.19 | -0.06 | -0.29 | 0.05 | -0.08 | 0.00 | 0.10 | 0.12 | -8.12 | 0.76 |
| Q608H | RRDR | 13.82 | 4 | 1 | 0.84 | 0.55 | 0.69 | -0.28 | -0.03 | -0.27 | 0.06 | -0.07 | -0.01 | 0.10 | 0.12 | -8.76 | 0.12 |
| Q608I | RRDR | 13.82 | 4 | 1 | 0.48 | 0.50 | 0.75 | -0.84 | -0.04 | -0.14 | 0.15 | -0.05 | 0.03 | 0.03 | 0.08 | -8.85 | 0.03 |
| Q608K | RRDR | 13.82 | 4 | 1 | 0.83 | 0.43 | 0.68 | -0.18 | -0.03 | -0.27 | 0.05 | -0.07 | 0.02 | 0.10 | 0.11 | -8.91 | -0.03 |
| Q608L | RRDR | 13.82 | 4 | 1 | 0.50 | 0.53 | 0.76 | -0.83 | -0.03 | -0.17 | 0.14 | -0.04 | 0.03 | 0.03 | 0.09 | -8.91 | -0.03 |
| Q608M | RRDR | 13.82 | 4 | 1 | 0.53 | 0.59 | 0.73 | -0.86 | -0.01 | -0.16 | 0.15 | -0.05 | 0.02 | 0.03 | 0.09 | -8.1 | 0.78 |
| Q608N | RRDR | 13.82 | 4 | 1 | 1.01 | 0.54 | 0.70 | -0.08 | -0.04 | -0.30 | 0.07 | -0.08 | -0.01 | 0.10 | 0.11 | -8.79 | 0.09 |
| Q608P | RRDR | 13.82 | 4 | 1 | 0.85 | 0.56 | 0.66 | -0.26 | -0.04 | -0.27 | 0.05 | -0.09 | -0.01 | 0.12 | 0.13 | -8.65 | 0.23 |
| Q608R | RRDR | 13.82 | 4 | 1 | 0.79 | 0.42 | 0.69 | -0.25 | -0.03 | -0.25 | 0.04 | -0.07 | 0.01 | 0.12 | 0.11 | -8.75 | 0.13 |
| Q608S | RRDR | 13.82 | 4 | 1 | 0.84 | 0.54 | 0.69 | -0.28 | -0.04 | -0.27 | 0.05 | -0.07 | -0.01 | 0.10 | 0.12 | -8.74 | 0.14 |
| Q608T | RRDR | 13.82 | 4 | 1 | 0.80 | 0.57 | 0.66 | -0.34 | -0.04 | -0.25 | 0.07 | -0.08 | -0.02 | 0.12 | 0.12 | -8.56 | 0.32 |
| Q608V | RRDR | 13.82 | 4 | 1 | 0.51 | 0.52 | 0.76 | -0.80 | -0.03 | -0.17 | 0.14 | -0.05 | 0.03 | 0.03 | 0.08 | -8.16 | 0.72 |
| Q608W | RRDR | 13.82 | 4 | 1 | 0.62 | 0.52 | 0.75 | -0.63 | -0.04 | -0.18 | 0.13 | -0.06 | 0.02 | 0.03 | 0.09 | -8.78 | 0.1 |
| Q608Y | RRDR | 13.82 | 4 | 1 | 0.46 | 0.53 | 0.70 | -0.82 | -0.03 | -0.15 | 0.14 | -0.04 | 0.02 | 0.02 | 0.10 | -8.7 | 0.18 |

Table S7. The predictive results for the classifiers on the total of 380 (20×19) mutated RpoB mutants in Rif resistance-determining regions (RRDRs) or non-RRDRs. P: positive, N: negative. P_N_ and P_P_ denote the probability values of being negative or positive mutation, respectively. NB: Naïve Bayes; DT: decision tree; kNN: k nearest neighbors; PNN: probabilistic neural network; SVM: support vector machine; MC: majority consensus

| Mutation | Region | DT | | | kNN | | | NB | | | PNN | | | SVM | | | MC | | |
| --- | --- | --- | --- | --- | --- | --- | --- | --- | --- | --- | --- | --- | --- | --- | --- | --- | --- | --- | --- |
|  |  | P_N_ | P_P_ | Prediction | P_N_ | P_P_ | Prediction | P_N_ | P_P_ | Prediction | P_N_ | P_P_ | Prediction | P_N_ | P_P_ | Prediction | P_N_ | P_P_ | Prediction |
| L38A | Non-RRDR | 0.00 | 1.00 | P | 0.63 | 0.37 | N | 0.97 | 0.03 | N | 0.65 | 0.35 | N | 0.63 | 0.37 | N | 0.80 | 0.20 | N |
| L38C | Non-RRDR | 0.00 | 1.00 | P | 0.62 | 0.38 | N | 0.99 | 0.01 | N | 0.57 | 0.43 | N | 0.69 | 0.31 | N | 0.80 | 0.20 | N |
| L38D | Non-RRDR | 0.00 | 1.00 | P | 0.63 | 0.37 | N | 0.70 | 0.30 | N | 0.65 | 0.35 | N | 0.54 | 0.46 | N | 0.80 | 0.20 | N |
| L38E | Non-RRDR | 0.00 | 1.00 | P | 0.64 | 0.36 | N | 0.93 | 0.07 | N | 0.67 | 0.33 | N | 0.59 | 0.41 | N | 0.80 | 0.20 | N |
| L38F | Non-RRDR | 0.00 | 1.00 | P | 0.62 | 0.38 | N | 1.00 | 0.00 | N | 0.59 | 0.41 | N | 0.74 | 0.26 | N | 0.80 | 0.20 | N |
| L38G | Non-RRDR | 0.00 | 1.00 | P | 0.63 | 0.37 | N | 0.95 | 0.05 | N | 0.63 | 0.37 | N | 0.56 | 0.44 | N | 0.80 | 0.20 | N |
| L38H | Non-RRDR | 0.00 | 1.00 | P | 0.63 | 0.37 | N | 0.97 | 0.03 | N | 0.66 | 0.34 | N | 0.62 | 0.38 | N | 0.80 | 0.20 | N |
| L38I | Non-RRDR | 0.00 | 1.00 | P | 0.38 | 0.62 | P | 1.00 | 0.00 | N | 0.81 | 0.19 | N | 0.86 | 0.14 | N | 0.60 | 0.40 | N |
| L38K | Non-RRDR | 0.00 | 1.00 | P | 0.63 | 0.37 | N | 0.96 | 0.04 | N | 0.65 | 0.35 | N | 0.61 | 0.39 | N | 0.80 | 0.20 | N |
| L38M | Non-RRDR | 0.00 | 1.00 | P | 0.62 | 0.38 | N | 1.00 | 0.00 | N | 0.58 | 0.42 | N | 0.72 | 0.28 | N | 0.80 | 0.20 | N |
| L38N | Non-RRDR | 0.00 | 1.00 | P | 0.63 | 0.37 | N | 0.86 | 0.14 | N | 0.66 | 0.34 | N | 0.60 | 0.40 | N | 0.80 | 0.20 | N |
| L38P | Non-RRDR | 0.00 | 1.00 | P | 0.63 | 0.37 | N | 0.98 | 0.02 | N | 0.65 | 0.35 | N | 0.63 | 0.37 | N | 0.80 | 0.20 | N |
| L38Q | Non-RRDR | 0.00 | 1.00 | P | 0.65 | 0.35 | N | 1.00 | 0.00 | N | 0.71 | 0.29 | N | 0.67 | 0.33 | N | 0.80 | 0.20 | N |
| L38R | Non-RRDR | 0.00 | 1.00 | P | 0.63 | 0.37 | N | 0.88 | 0.12 | N | 0.66 | 0.34 | N | 0.63 | 0.37 | N | 0.80 | 0.20 | N |
| L38S | Non-RRDR | 0.00 | 1.00 | P | 0.63 | 0.37 | N | 0.96 | 0.04 | N | 0.65 | 0.35 | N | 0.62 | 0.38 | N | 0.80 | 0.20 | N |
| L38T | Non-RRDR | 0.00 | 1.00 | P | 0.63 | 0.37 | N | 0.98 | 0.02 | N | 0.63 | 0.37 | N | 0.63 | 0.37 | N | 0.80 | 0.20 | N |
| L38V | Non-RRDR | 0.92 | 0.08 | N | 0.37 | 0.63 | P | 1.00 | 0.00 | N | 0.69 | 0.31 | N | 0.80 | 0.20 | N | 0.80 | 0.20 | N |
| L38W | Non-RRDR | 0.00 | 1.00 | P | 0.62 | 0.38 | N | 0.99 | 0.01 | N | 0.57 | 0.43 | N | 0.68 | 0.32 | N | 0.80 | 0.20 | N |
| L38Y | Non-RRDR | 0.00 | 1.00 | P | 0.61 | 0.39 | N | 1.00 | 0.00 | N | 0.56 | 0.44 | N | 0.72 | 0.28 | N | 0.80 | 0.20 | N |
| K119A | Non-RRDR | 0.33 | 0.67 | P | 0.20 | 0.80 | P | 0.98 | 0.02 | N | 0.39 | 0.61 | P | 0.47 | 0.53 | P | 0.20 | 0.80 | P |
| K119C | Non-RRDR | 0.92 | 0.08 | N | 0.21 | 0.79 | P | 0.98 | 0.02 | N | 0.42 | 0.58 | P | 0.51 | 0.49 | N | 0.60 | 0.40 | N |
| K119D | Non-RRDR | 0.00 | 1.00 | P | 0.18 | 0.82 | P | 0.95 | 0.05 | N | 0.35 | 0.65 | P | 0.46 | 0.54 | P | 0.20 | 0.80 | P |
| K119E | Non-RRDR | 0.00 | 1.00 | P | 0.18 | 0.82 | P | 0.99 | 0.01 | N | 0.36 | 0.64 | P | 0.47 | 0.53 | P | 0.20 | 0.80 | P |
| K119F | Non-RRDR | 0.33 | 0.67 | P | 0.25 | 0.75 | P | 0.93 | 0.07 | N | 0.46 | 0.54 | P | 0.51 | 0.49 | N | 0.40 | 0.60 | P |
| K119G | Non-RRDR | 0.92 | 0.08 | N | 0.22 | 0.78 | P | 0.98 | 0.02 | N | 0.36 | 0.64 | P | 0.41 | 0.59 | P | 0.40 | 0.60 | P |
| K119H | Non-RRDR | 0.67 | 0.33 | N | 0.58 | 0.42 | N | 1.00 | 0.00 | N | 0.64 | 0.36 | N | 0.61 | 0.39 | N | 1.00 | 0.00 | N |
| K119I | Non-RRDR | 0.92 | 0.08 | N | 0.25 | 0.75 | P | 0.97 | 0.03 | N | 0.47 | 0.53 | P | 0.52 | 0.48 | N | 0.60 | 0.40 | N |
| K119L | Non-RRDR | 0.33 | 0.67 | P | 0.25 | 0.75 | P | 0.72 | 0.28 | N | 0.46 | 0.54 | P | 0.51 | 0.49 | N | 0.40 | 0.60 | P |
| K119M | Non-RRDR | 0.92 | 0.08 | N | 0.25 | 0.75 | P | 0.98 | 0.02 | N | 0.46 | 0.54 | P | 0.52 | 0.48 | N | 0.60 | 0.40 | N |
| K119N | Non-RRDR | 0.92 | 0.08 | N | 0.19 | 0.81 | P | 0.99 | 0.01 | N | 0.36 | 0.64 | P | 0.46 | 0.54 | P | 0.40 | 0.60 | P |
| K119P | Non-RRDR | 0.33 | 0.67 | P | 0.21 | 0.79 | P | 0.97 | 0.03 | N | 0.38 | 0.62 | P | 0.44 | 0.56 | P | 0.20 | 0.80 | P |
| K119Q | Non-RRDR | 0.92 | 0.08 | N | 0.19 | 0.81 | P | 1.00 | 0.00 | N | 0.39 | 0.61 | P | 0.53 | 0.47 | N | 0.60 | 0.40 | N |
| K119R | Non-RRDR | 1.00 | 0.00 | N | 0.63 | 0.37 | N | 1.00 | 0.00 | N | 0.82 | 0.18 | N | 0.76 | 0.24 | N | 1.00 | 0.00 | N |
| K119S | Non-RRDR | 0.92 | 0.08 | N | 0.19 | 0.81 | P | 0.90 | 0.10 | N | 0.37 | 0.63 | P | 0.46 | 0.54 | P | 0.40 | 0.60 | P |
| K119T | Non-RRDR | 0.92 | 0.08 | N | 0.20 | 0.80 | P | 0.99 | 0.01 | N | 0.40 | 0.60 | P | 0.48 | 0.52 | P | 0.40 | 0.60 | P |
| K119V | Non-RRDR | 0.33 | 0.67 | P | 0.24 | 0.76 | P | 0.98 | 0.02 | N | 0.44 | 0.56 | P | 0.49 | 0.51 | P | 0.20 | 0.80 | P |
| K119W | Non-RRDR | 0.33 | 0.67 | P | 0.21 | 0.79 | P | 0.99 | 0.01 | N | 0.41 | 0.59 | P | 0.50 | 0.50 | P | 0.20 | 0.80 | P |
| K119Y | Non-RRDR | 0.92 | 0.08 | N | 0.25 | 0.75 | P | 0.92 | 0.08 | N | 0.48 | 0.52 | P | 0.52 | 0.48 | N | 0.60 | 0.40 | N |
| E132A | Non-RRDR | 0.92 | 0.08 | N | 1.00 | 0.00 | N | 0.97 | 0.03 | N | 0.95 | 0.05 | N | 0.76 | 0.24 | N | 1.00 | 0.00 | N |
| E132C | Non-RRDR | 0.92 | 0.08 | N | 1.00 | 0.00 | N | 0.08 | 0.92 | P | 0.95 | 0.05 | N | 0.74 | 0.26 | N | 0.80 | 0.20 | N |
| E132D | Non-RRDR | 0.92 | 0.08 | N | 1.00 | 0.00 | N | 1.00 | 0.00 | N | 0.94 | 0.06 | N | 0.75 | 0.25 | N | 1.00 | 0.00 | N |
| E132F | Non-RRDR | 0.92 | 0.08 | N | 1.00 | 0.00 | N | 0.98 | 0.02 | N | 0.95 | 0.05 | N | 0.74 | 0.26 | N | 1.00 | 0.00 | N |
| E132G | Non-RRDR | 0.33 | 0.67 | P | 1.00 | 0.00 | N | 0.98 | 0.02 | N | 0.94 | 0.06 | N | 0.72 | 0.28 | N | 0.80 | 0.20 | N |
| E132H | Non-RRDR | 0.92 | 0.08 | N | 1.00 | 0.00 | N | 0.99 | 0.01 | N | 0.94 | 0.06 | N | 0.76 | 0.24 | N | 1.00 | 0.00 | N |
| E132I | Non-RRDR | 0.92 | 0.08 | N | 1.00 | 0.00 | N | 0.98 | 0.02 | N | 0.95 | 0.05 | N | 0.76 | 0.24 | N | 1.00 | 0.00 | N |
| E132K | Non-RRDR | 0.92 | 0.08 | N | 1.00 | 0.00 | N | 1.00 | 0.00 | N | 0.92 | 0.08 | N | 0.83 | 0.17 | N | 1.00 | 0.00 | N |
| E132L | Non-RRDR | 0.92 | 0.08 | N | 1.00 | 0.00 | N | 0.98 | 0.02 | N | 0.95 | 0.05 | N | 0.76 | 0.24 | N | 1.00 | 0.00 | N |
| E132M | Non-RRDR | 0.92 | 0.08 | N | 1.00 | 0.00 | N | 0.99 | 0.01 | N | 0.95 | 0.05 | N | 0.79 | 0.21 | N | 1.00 | 0.00 | N |
| E132N | Non-RRDR | 0.92 | 0.08 | N | 1.00 | 0.00 | N | 1.00 | 0.00 | N | 0.95 | 0.05 | N | 0.76 | 0.24 | N | 1.00 | 0.00 | N |
| E132P | Non-RRDR | 0.33 | 0.67 | P | 1.00 | 0.00 | N | 0.98 | 0.02 | N | 0.95 | 0.05 | N | 0.74 | 0.26 | N | 0.80 | 0.20 | N |
| E132Q | Non-RRDR | 0.67 | 0.33 | N | 0.74 | 0.26 | N | 1.00 | 0.00 | N | 0.88 | 0.12 | N | 0.83 | 0.17 | N | 1.00 | 0.00 | N |
| E132R | Non-RRDR | 0.00 | 1.00 | P | 0.70 | 0.30 | N | 1.00 | 0.00 | N | 0.92 | 0.08 | N | 0.91 | 0.09 | N | 0.80 | 0.20 | N |
| E132S | Non-RRDR | 0.92 | 0.08 | N | 1.00 | 0.00 | N | 0.99 | 0.01 | N | 0.94 | 0.06 | N | 0.75 | 0.25 | N | 1.00 | 0.00 | N |
| E132T | Non-RRDR | 0.92 | 0.08 | N | 1.00 | 0.00 | N | 0.93 | 0.07 | N | 0.95 | 0.05 | N | 0.76 | 0.24 | N | 1.00 | 0.00 | N |
| E132V | Non-RRDR | 0.92 | 0.08 | N | 1.00 | 0.00 | N | 0.91 | 0.09 | N | 0.95 | 0.05 | N | 0.76 | 0.24 | N | 1.00 | 0.00 | N |
| E132W | Non-RRDR | 0.92 | 0.08 | N | 1.00 | 0.00 | N | 0.96 | 0.04 | N | 0.95 | 0.05 | N | 0.75 | 0.25 | N | 1.00 | 0.00 | N |
| E132Y | Non-RRDR | 0.92 | 0.08 | N | 1.00 | 0.00 | N | 0.99 | 0.01 | N | 0.95 | 0.05 | N | 0.75 | 0.25 | N | 1.00 | 0.00 | N |
| I185A | Non-RRDR | 0.00 | 1.00 | P | 0.69 | 0.31 | N | 1.00 | 0.00 | N | 0.95 | 0.05 | N | 0.67 | 0.33 | N | 0.80 | 0.20 | N |
| I185C | Non-RRDR | 0.00 | 1.00 | P | 0.68 | 0.32 | N | 1.00 | 0.00 | N | 0.92 | 0.08 | N | 0.67 | 0.33 | N | 0.80 | 0.20 | N |
| I185D | Non-RRDR | 0.00 | 1.00 | P | 0.68 | 0.32 | N | 0.71 | 0.29 | N | 0.95 | 0.05 | N | 0.52 | 0.48 | N | 0.80 | 0.20 | N |
| I185E | Non-RRDR | 0.00 | 1.00 | P | 0.68 | 0.32 | N | 0.86 | 0.14 | N | 0.95 | 0.05 | N | 0.58 | 0.42 | N | 0.80 | 0.20 | N |
| I185F | Non-RRDR | 0.92 | 0.08 | N | 0.67 | 0.33 | N | 1.00 | 0.00 | N | 0.92 | 0.08 | N | 0.73 | 0.27 | N | 1.00 | 0.00 | N |
| I185G | Non-RRDR | 0.00 | 1.00 | P | 0.68 | 0.32 | N | 0.04 | 0.96 | P | 0.95 | 0.05 | N | 0.52 | 0.48 | N | 0.60 | 0.40 | N |
| I185H | Non-RRDR | 0.00 | 1.00 | P | 0.68 | 0.32 | N | 1.00 | 0.00 | N | 0.97 | 0.03 | N | 0.68 | 0.32 | N | 0.80 | 0.20 | N |
| I185K | Non-RRDR | 0.00 | 1.00 | P | 0.68 | 0.32 | N | 0.99 | 0.01 | N | 0.95 | 0.05 | N | 0.63 | 0.37 | N | 0.80 | 0.20 | N |
| I185L | Non-RRDR | 0.92 | 0.08 | N | 0.69 | 0.31 | N | 1.00 | 0.00 | N | 0.94 | 0.06 | N | 0.75 | 0.25 | N | 1.00 | 0.00 | N |
| I185M | Non-RRDR | 0.92 | 0.08 | N | 0.69 | 0.31 | N | 1.00 | 0.00 | N | 0.95 | 0.05 | N | 0.76 | 0.24 | N | 1.00 | 0.00 | N |
| I185N | Non-RRDR | 0.00 | 1.00 | P | 0.68 | 0.32 | N | 0.77 | 0.23 | N | 0.95 | 0.05 | N | 0.55 | 0.45 | N | 0.80 | 0.20 | N |
| I185P | Non-RRDR | 0.00 | 1.00 | P | 0.68 | 0.32 | N | 1.00 | 0.00 | N | 0.95 | 0.05 | N | 0.66 | 0.34 | N | 0.80 | 0.20 | N |
| I185Q | Non-RRDR | 0.00 | 1.00 | P | 0.68 | 0.32 | N | 0.97 | 0.03 | N | 0.95 | 0.05 | N | 0.63 | 0.37 | N | 0.80 | 0.20 | N |
| I185R | Non-RRDR | 0.00 | 1.00 | P | 0.68 | 0.32 | N | 0.99 | 0.01 | N | 0.95 | 0.05 | N | 0.63 | 0.37 | N | 0.80 | 0.20 | N |
| I185S | Non-RRDR | 0.00 | 1.00 | P | 0.68 | 0.32 | N | 0.97 | 0.03 | N | 0.95 | 0.05 | N | 0.59 | 0.41 | N | 0.80 | 0.20 | N |
| I185T | Non-RRDR | 0.00 | 1.00 | P | 0.69 | 0.31 | N | 1.00 | 0.00 | N | 0.95 | 0.05 | N | 0.70 | 0.30 | N | 0.80 | 0.20 | N |
| I185V | Non-RRDR | 0.92 | 0.08 | N | 0.70 | 0.30 | N | 1.00 | 0.00 | N | 0.96 | 0.04 | N | 0.78 | 0.22 | N | 1.00 | 0.00 | N |
| I185W | Non-RRDR | 0.00 | 1.00 | P | 0.67 | 0.33 | N | 0.99 | 0.01 | N | 0.91 | 0.09 | N | 0.64 | 0.36 | N | 0.80 | 0.20 | N |
| I185Y | Non-RRDR | 0.92 | 0.08 | N | 0.68 | 0.32 | N | 1.00 | 0.00 | N | 0.95 | 0.05 | N | 0.78 | 0.22 | N | 1.00 | 0.00 | N |
| G236A | Non-RRDR | 0.00 | 1.00 | P | 0.28 | 0.72 | P | 0.97 | 0.03 | N | 0.08 | 0.92 | P | 0.76 | 0.24 | N | 0.40 | 0.60 | P |
| G236C | Non-RRDR | 0.00 | 1.00 | P | 0.28 | 0.72 | P | 0.85 | 0.15 | N | 0.07 | 0.93 | P | 0.71 | 0.29 | N | 0.40 | 0.60 | P |
| G236D | Non-RRDR | 0.00 | 1.00 | P | 0.28 | 0.72 | P | 0.99 | 0.01 | N | 0.08 | 0.92 | P | 0.77 | 0.23 | N | 0.40 | 0.60 | P |
| G236E | Non-RRDR | 0.00 | 1.00 | P | 0.28 | 0.72 | P | 0.99 | 0.01 | N | 0.07 | 0.93 | P | 0.74 | 0.26 | N | 0.40 | 0.60 | P |
| G236F | Non-RRDR | 0.00 | 1.00 | P | 0.28 | 0.72 | P | 0.95 | 0.05 | N | 0.07 | 0.93 | P | 0.72 | 0.28 | N | 0.40 | 0.60 | P |
| G236H | Non-RRDR | 0.00 | 1.00 | P | 0.28 | 0.72 | P | 0.99 | 0.01 | N | 0.07 | 0.93 | P | 0.73 | 0.27 | N | 0.40 | 0.60 | P |
| G236I | Non-RRDR | 0.00 | 1.00 | P | 0.28 | 0.72 | P | 0.92 | 0.08 | N | 0.07 | 0.93 | P | 0.71 | 0.29 | N | 0.40 | 0.60 | P |
| G236K | Non-RRDR | 0.00 | 1.00 | P | 0.28 | 0.72 | P | 0.99 | 0.01 | N | 0.07 | 0.93 | P | 0.74 | 0.26 | N | 0.40 | 0.60 | P |
| G236L | Non-RRDR | 0.00 | 1.00 | P | 0.28 | 0.72 | P | 0.93 | 0.07 | N | 0.07 | 0.93 | P | 0.72 | 0.28 | N | 0.40 | 0.60 | P |
| G236M | Non-RRDR | 0.00 | 1.00 | P | 0.28 | 0.72 | P | 0.81 | 0.19 | N | 0.07 | 0.93 | P | 0.71 | 0.29 | N | 0.40 | 0.60 | P |
| G236N | Non-RRDR | 0.00 | 1.00 | P | 0.28 | 0.72 | P | 0.99 | 0.01 | N | 0.08 | 0.92 | P | 0.77 | 0.23 | N | 0.40 | 0.60 | P |
| G236P | Non-RRDR | 0.00 | 1.00 | P | 0.28 | 0.72 | P | 0.96 | 0.04 | N | 0.07 | 0.93 | P | 0.73 | 0.27 | N | 0.40 | 0.60 | P |
| G236Q | Non-RRDR | 0.00 | 1.00 | P | 0.28 | 0.72 | P | 0.71 | 0.29 | N | 0.07 | 0.93 | P | 0.73 | 0.27 | N | 0.40 | 0.60 | P |
| G236R | Non-RRDR | 0.00 | 1.00 | P | 0.28 | 0.72 | P | 0.97 | 0.03 | N | 0.07 | 0.93 | P | 0.73 | 0.27 | N | 0.40 | 0.60 | P |
| G236S | Non-RRDR | 0.00 | 1.00 | P | 0.28 | 0.72 | P | 0.72 | 0.28 | N | 0.08 | 0.92 | P | 0.76 | 0.24 | N | 0.40 | 0.60 | P |
| G236T | Non-RRDR | 0.00 | 1.00 | P | 0.28 | 0.72 | P | 0.92 | 0.08 | N | 0.07 | 0.93 | P | 0.73 | 0.27 | N | 0.40 | 0.60 | P |
| G236V | Non-RRDR | 0.00 | 1.00 | P | 0.28 | 0.72 | P | 0.65 | 0.35 | N | 0.06 | 0.94 | P | 0.67 | 0.33 | N | 0.40 | 0.60 | P |
| G236W | Non-RRDR | 0.00 | 1.00 | P | 0.28 | 0.72 | P | 0.94 | 0.06 | N | 0.07 | 0.93 | P | 0.72 | 0.28 | N | 0.40 | 0.60 | P |
| G236Y | Non-RRDR | 0.00 | 1.00 | P | 0.28 | 0.72 | P | 0.95 | 0.05 | N | 0.07 | 0.93 | P | 0.73 | 0.27 | N | 0.40 | 0.60 | P |
| E244A | Non-RRDR | 0.00 | 1.00 | P | 0.25 | 0.75 | P | 1.00 | 0.00 | N | 0.33 | 0.67 | P | 0.88 | 0.12 | N | 0.40 | 0.60 | P |
| E244C | Non-RRDR | 0.00 | 1.00 | P | 0.24 | 0.76 | P | 1.00 | 0.00 | N | 0.32 | 0.68 | P | 0.86 | 0.14 | N | 0.40 | 0.60 | P |
| E244D | Non-RRDR | 0.67 | 0.33 | N | 0.27 | 0.73 | P | 1.00 | 0.00 | N | 0.34 | 0.66 | P | 0.90 | 0.10 | N | 0.60 | 0.40 | N |
| E244F | Non-RRDR | 0.00 | 1.00 | P | 0.24 | 0.76 | P | 1.00 | 0.00 | N | 0.32 | 0.68 | P | 0.86 | 0.14 | N | 0.40 | 0.60 | P |
| E244G | Non-RRDR | 0.00 | 1.00 | P | 0.24 | 0.76 | P | 1.00 | 0.00 | N | 0.32 | 0.68 | P | 0.86 | 0.14 | N | 0.40 | 0.60 | P |
| E244H | Non-RRDR | 0.00 | 1.00 | P | 0.25 | 0.75 | P | 1.00 | 0.00 | N | 0.33 | 0.67 | P | 0.88 | 0.12 | N | 0.40 | 0.60 | P |
| E244I | Non-RRDR | 0.00 | 1.00 | P | 0.24 | 0.76 | P | 1.00 | 0.00 | N | 0.32 | 0.68 | P | 0.86 | 0.14 | N | 0.40 | 0.60 | P |
| E244K | Non-RRDR | 0.00 | 1.00 | P | 0.25 | 0.75 | P | 1.00 | 0.00 | N | 0.33 | 0.67 | P | 0.88 | 0.12 | N | 0.40 | 0.60 | P |
| E244L | Non-RRDR | 0.00 | 1.00 | P | 0.24 | 0.76 | P | 1.00 | 0.00 | N | 0.31 | 0.69 | P | 0.86 | 0.14 | N | 0.40 | 0.60 | P |
| E244M | Non-RRDR | 0.00 | 1.00 | P | 0.24 | 0.76 | P | 1.00 | 0.00 | N | 0.32 | 0.68 | P | 0.87 | 0.13 | N | 0.40 | 0.60 | P |
| E244N | Non-RRDR | 0.00 | 1.00 | P | 0.26 | 0.74 | P | 1.00 | 0.00 | N | 0.33 | 0.67 | P | 0.90 | 0.10 | N | 0.40 | 0.60 | P |
| E244P | Non-RRDR | 0.00 | 1.00 | P | 0.24 | 0.76 | P | 1.00 | 0.00 | N | 0.32 | 0.68 | P | 0.86 | 0.14 | N | 0.40 | 0.60 | P |
| E244Q | Non-RRDR | 0.00 | 1.00 | P | 0.25 | 0.75 | P | 1.00 | 0.00 | N | 0.33 | 0.67 | P | 0.89 | 0.11 | N | 0.40 | 0.60 | P |
| E244R | Non-RRDR | 0.00 | 1.00 | P | 0.24 | 0.76 | P | 1.00 | 0.00 | N | 0.32 | 0.68 | P | 0.87 | 0.13 | N | 0.40 | 0.60 | P |
| E244S | Non-RRDR | 0.00 | 1.00 | P | 0.24 | 0.76 | P | 1.00 | 0.00 | N | 0.32 | 0.68 | P | 0.87 | 0.13 | N | 0.40 | 0.60 | P |
| E244T | Non-RRDR | 0.00 | 1.00 | P | 0.24 | 0.76 | P | 1.00 | 0.00 | N | 0.32 | 0.68 | P | 0.86 | 0.14 | N | 0.40 | 0.60 | P |
| E244V | Non-RRDR | 0.00 | 1.00 | P | 0.24 | 0.76 | P | 1.00 | 0.00 | N | 0.32 | 0.68 | P | 0.87 | 0.13 | N | 0.40 | 0.60 | P |
| E244W | Non-RRDR | 0.00 | 1.00 | P | 0.24 | 0.76 | P | 1.00 | 0.00 | N | 0.31 | 0.69 | P | 0.86 | 0.14 | N | 0.40 | 0.60 | P |
| E244Y | Non-RRDR | 0.00 | 1.00 | P | 0.24 | 0.76 | P | 1.00 | 0.00 | N | 0.32 | 0.68 | P | 0.86 | 0.14 | N | 0.40 | 0.60 | P |
| I336A | Non-RRDR | 1.00 | 0.00 | N | 1.00 | 0.00 | N | 0.20 | 0.80 | P | 0.94 | 0.06 | N | 0.43 | 0.57 | P | 0.60 | 0.40 | N |
| I336C | Non-RRDR | 1.00 | 0.00 | N | 0.67 | 0.33 | N | 0.80 | 0.20 | N | 0.89 | 0.11 | N | 0.57 | 0.43 | N | 1.00 | 0.00 | N |
| I336D | Non-RRDR | 1.00 | 0.00 | N | 1.00 | 0.00 | N | 0.12 | 0.88 | P | 0.94 | 0.06 | N | 0.42 | 0.58 | P | 0.60 | 0.40 | N |
| I336E | Non-RRDR | 1.00 | 0.00 | N | 1.00 | 0.00 | N | 0.07 | 0.93 | P | 0.94 | 0.06 | N | 0.42 | 0.58 | P | 0.60 | 0.40 | N |
| I336F | Non-RRDR | 1.00 | 0.00 | N | 0.63 | 0.37 | N | 0.79 | 0.21 | N | 0.82 | 0.18 | N | 0.67 | 0.33 | N | 1.00 | 0.00 | N |
| I336G | Non-RRDR | 1.00 | 0.00 | N | 1.00 | 0.00 | N | 0.09 | 0.91 | P | 0.94 | 0.06 | N | 0.42 | 0.58 | P | 0.60 | 0.40 | N |
| I336H | Non-RRDR | 1.00 | 0.00 | N | 0.68 | 0.32 | N | 0.16 | 0.84 | P | 0.89 | 0.11 | N | 0.51 | 0.49 | N | 0.80 | 0.20 | N |
| I336K | Non-RRDR | 1.00 | 0.00 | N | 1.00 | 0.00 | N | 0.22 | 0.78 | P | 0.94 | 0.06 | N | 0.44 | 0.56 | P | 0.60 | 0.40 | N |
| I336L | Non-RRDR | 0.00 | 1.00 | P | 0.52 | 0.48 | N | 1.00 | 0.00 | N | 0.62 | 0.38 | N | 0.70 | 0.30 | N | 0.80 | 0.20 | N |
| I336M | Non-RRDR | 0.00 | 1.00 | P | 0.57 | 0.43 | N | 0.99 | 0.01 | N | 0.66 | 0.34 | N | 0.67 | 0.33 | N | 0.80 | 0.20 | N |
| I336N | Non-RRDR | 1.00 | 0.00 | N | 1.00 | 0.00 | N | 0.19 | 0.81 | P | 0.94 | 0.06 | N | 0.43 | 0.57 | P | 0.60 | 0.40 | N |
| I336P | Non-RRDR | 1.00 | 0.00 | N | 1.00 | 0.00 | N | 0.13 | 0.87 | P | 0.94 | 0.06 | N | 0.44 | 0.56 | P | 0.60 | 0.40 | N |
| I336Q | Non-RRDR | 1.00 | 0.00 | N | 1.00 | 0.00 | N | 0.10 | 0.90 | P | 0.94 | 0.06 | N | 0.43 | 0.57 | P | 0.60 | 0.40 | N |
| I336R | Non-RRDR | 1.00 | 0.00 | N | 0.69 | 0.31 | N | 0.11 | 0.89 | P | 0.93 | 0.07 | N | 0.46 | 0.54 | P | 0.60 | 0.40 | N |
| I336S | Non-RRDR | 1.00 | 0.00 | N | 1.00 | 0.00 | N | 0.23 | 0.77 | P | 0.94 | 0.06 | N | 0.44 | 0.56 | P | 0.60 | 0.40 | N |
| I336T | Non-RRDR | 1.00 | 0.00 | N | 0.69 | 0.31 | N | 0.41 | 0.59 | P | 0.94 | 0.06 | N | 0.45 | 0.55 | P | 0.60 | 0.40 | N |
| I336V | Non-RRDR | 0.92 | 0.08 | N | 0.50 | 0.50 | N | 1.00 | 0.00 | N | 0.60 | 0.40 | N | 0.72 | 0.28 | N | 1.00 | 0.00 | N |
| I336W | Non-RRDR | 1.00 | 0.00 | N | 0.67 | 0.33 | N | 0.37 | 0.63 | P | 0.89 | 0.11 | N | 0.58 | 0.42 | N | 0.80 | 0.20 | N |
| I336Y | Non-RRDR | 1.00 | 0.00 | N | 0.66 | 0.34 | N | 0.69 | 0.31 | N | 0.87 | 0.13 | N | 0.61 | 0.39 | N | 1.00 | 0.00 | N |
| R373A | Non-RRDR | 0.75 | 0.25 | N | 0.25 | 0.75 | P | 0.14 | 0.86 | P | 0.10 | 0.90 | P | 0.31 | 0.69 | P | 0.20 | 0.80 | P |
| R373C | Non-RRDR | 0.00 | 1.00 | P | 0.26 | 0.74 | P | 0.47 | 0.53 | P | 0.10 | 0.90 | P | 0.32 | 0.68 | P | 0.00 | 1.00 | P |
| R373D | Non-RRDR | 0.04 | 0.96 | P | 0.25 | 0.75 | P | 0.36 | 0.64 | P | 0.10 | 0.90 | P | 0.31 | 0.69 | P | 0.00 | 1.00 | P |
| R373E | Non-RRDR | 0.04 | 0.96 | P | 0.25 | 0.75 | P | 0.52 | 0.48 | N | 0.10 | 0.90 | P | 0.32 | 0.68 | P | 0.20 | 0.80 | P |
| R373F | Non-RRDR | 0.00 | 1.00 | P | 0.27 | 0.73 | P | 0.03 | 0.97 | P | 0.09 | 0.91 | P | 0.33 | 0.67 | P | 0.00 | 1.00 | P |
| R373G | Non-RRDR | 0.75 | 0.25 | N | 0.24 | 0.76 | P | 0.07 | 0.93 | P | 0.09 | 0.91 | P | 0.30 | 0.70 | P | 0.20 | 0.80 | P |
| R373H | Non-RRDR | 0.75 | 0.25 | N | 0.25 | 0.75 | P | 0.19 | 0.81 | P | 0.10 | 0.90 | P | 0.30 | 0.70 | P | 0.20 | 0.80 | P |
| R373I | Non-RRDR | 0.00 | 1.00 | P | 0.27 | 0.73 | P | 0.25 | 0.75 | P | 0.09 | 0.91 | P | 0.33 | 0.67 | P | 0.00 | 1.00 | P |
| R373K | Non-RRDR | 0.00 | 1.00 | P | 0.28 | 0.72 | P | 0.54 | 0.46 | N | 0.13 | 0.87 | P | 0.33 | 0.67 | P | 0.20 | 0.80 | P |
| R373L | Non-RRDR | 0.75 | 0.25 | N | 0.27 | 0.73 | P | 0.34 | 0.66 | P | 0.09 | 0.91 | P | 0.34 | 0.66 | P | 0.20 | 0.80 | P |
| R373M | Non-RRDR | 0.75 | 0.25 | N | 0.26 | 0.74 | P | 0.10 | 0.90 | P | 0.09 | 0.91 | P | 0.32 | 0.68 | P | 0.20 | 0.80 | P |
| R373N | Non-RRDR | 0.75 | 0.25 | N | 0.24 | 0.76 | P | 0.34 | 0.66 | P | 0.09 | 0.91 | P | 0.28 | 0.72 | P | 0.20 | 0.80 | P |
| R373P | Non-RRDR | 0.75 | 0.25 | N | 0.25 | 0.75 | P | 0.36 | 0.64 | P | 0.10 | 0.90 | P | 0.31 | 0.69 | P | 0.20 | 0.80 | P |
| R373Q | Non-RRDR | 0.75 | 0.25 | N | 0.25 | 0.75 | P | 0.29 | 0.71 | P | 0.10 | 0.90 | P | 0.30 | 0.70 | P | 0.20 | 0.80 | P |
| R373S | Non-RRDR | 0.75 | 0.25 | N | 0.24 | 0.76 | P | 0.08 | 0.92 | P | 0.09 | 0.91 | P | 0.29 | 0.71 | P | 0.20 | 0.80 | P |
| R373T | Non-RRDR | 0.75 | 0.25 | N | 0.25 | 0.75 | P | 0.33 | 0.67 | P | 0.10 | 0.90 | P | 0.32 | 0.68 | P | 0.20 | 0.80 | P |
| R373V | Non-RRDR | 0.00 | 1.00 | P | 0.26 | 0.74 | P | 0.06 | 0.94 | P | 0.09 | 0.91 | P | 0.30 | 0.70 | P | 0.00 | 1.00 | P |
| R373W | Non-RRDR | 0.00 | 1.00 | P | 0.26 | 0.74 | P | 0.02 | 0.98 | P | 0.10 | 0.90 | P | 0.32 | 0.68 | P | 0.00 | 1.00 | P |
| R373Y | Non-RRDR | 0.00 | 1.00 | P | 0.27 | 0.73 | P | 0.07 | 0.93 | P | 0.09 | 0.91 | P | 0.33 | 0.67 | P | 0.00 | 1.00 | P |
| T400A | Non-RRDR | 0.92 | 0.08 | N | 0.37 | 0.63 | P | 1.00 | 0.00 | N | 0.53 | 0.47 | N | 0.78 | 0.22 | N | 0.80 | 0.20 | N |
| T400C | Non-RRDR | 0.92 | 0.08 | N | 0.43 | 0.57 | P | 0.99 | 0.01 | N | 0.57 | 0.43 | N | 0.78 | 0.22 | N | 0.80 | 0.20 | N |
| T400D | Non-RRDR | 0.00 | 1.00 | P | 0.42 | 0.58 | P | 1.00 | 0.00 | N | 0.54 | 0.46 | N | 0.76 | 0.24 | N | 0.60 | 0.40 | N |
| T400E | Non-RRDR | 0.00 | 1.00 | P | 0.41 | 0.59 | P | 1.00 | 0.00 | N | 0.54 | 0.46 | N | 0.76 | 0.24 | N | 0.60 | 0.40 | N |
| T400F | Non-RRDR | 0.92 | 0.08 | N | 0.30 | 0.70 | P | 0.00 | 1.00 | P | 0.60 | 0.40 | N | 0.81 | 0.19 | N | 0.60 | 0.40 | N |
| T400G | Non-RRDR | 0.92 | 0.08 | N | 0.41 | 0.59 | P | 0.99 | 0.01 | N | 0.54 | 0.46 | N | 0.75 | 0.25 | N | 0.80 | 0.20 | N |
| T400H | Non-RRDR | 0.92 | 0.08 | N | 0.41 | 0.59 | P | 1.00 | 0.00 | N | 0.54 | 0.46 | N | 0.75 | 0.25 | N | 0.80 | 0.20 | N |
| T400I | Non-RRDR | 0.00 | 1.00 | P | 0.01 | 0.99 | P | 1.00 | 0.00 | N | 0.32 | 0.68 | P | 0.91 | 0.09 | N | 0.40 | 0.60 | P |
| T400K | Non-RRDR | 0.92 | 0.08 | N | 0.41 | 0.59 | P | 1.00 | 0.00 | N | 0.54 | 0.46 | N | 0.75 | 0.25 | N | 0.80 | 0.20 | N |
| T400L | Non-RRDR | 0.92 | 0.08 | N | 0.23 | 0.77 | P | 1.00 | 0.00 | N | 0.43 | 0.57 | P | 0.88 | 0.12 | N | 0.60 | 0.40 | N |
| T400M | Non-RRDR | 0.92 | 0.08 | N | 0.23 | 0.77 | P | 1.00 | 0.00 | N | 0.43 | 0.57 | P | 0.86 | 0.14 | N | 0.60 | 0.40 | N |
| T400N | Non-RRDR | 0.00 | 1.00 | P | 0.41 | 0.59 | P | 1.00 | 0.00 | N | 0.54 | 0.46 | N | 0.76 | 0.24 | N | 0.60 | 0.40 | N |
| T400P | Non-RRDR | 0.92 | 0.08 | N | 0.42 | 0.58 | P | 0.99 | 0.01 | N | 0.54 | 0.46 | N | 0.75 | 0.25 | N | 0.80 | 0.20 | N |
| T400Q | Non-RRDR | 0.92 | 0.08 | N | 0.41 | 0.59 | P | 1.00 | 0.00 | N | 0.54 | 0.46 | N | 0.76 | 0.24 | N | 0.80 | 0.20 | N |
| T400R | Non-RRDR | 0.92 | 0.08 | N | 0.42 | 0.58 | P | 0.98 | 0.02 | N | 0.54 | 0.46 | N | 0.75 | 0.25 | N | 0.80 | 0.20 | N |
| T400S | Non-RRDR | 0.92 | 0.08 | N | 0.19 | 0.81 | P | 1.00 | 0.00 | N | 0.50 | 0.50 | N | 0.79 | 0.21 | N | 0.80 | 0.20 | N |
| T400V | Non-RRDR | 0.92 | 0.08 | N | 0.20 | 0.80 | P | 1.00 | 0.00 | N | 0.37 | 0.63 | P | 0.88 | 0.12 | N | 0.60 | 0.40 | N |
| T400W | Non-RRDR | 0.33 | 0.67 | P | 0.47 | 0.53 | P | 1.00 | 0.00 | N | 0.59 | 0.41 | N | 0.77 | 0.23 | N | 0.60 | 0.40 | N |
| T400Y | Non-RRDR | 0.92 | 0.08 | N | 0.28 | 0.72 | P | 1.00 | 0.00 | N | 0.59 | 0.41 | N | 0.81 | 0.19 | N | 0.80 | 0.20 | N |
| G475A | Non-RRDR | 1.00 | 0.00 | N | 0.38 | 0.62 | P | 0.08 | 0.92 | P | 0.65 | 0.35 | N | 0.39 | 0.61 | P | 0.40 | 0.60 | P |
| G475C | Non-RRDR | 1.00 | 0.00 | N | 0.37 | 0.63 | P | 0.10 | 0.90 | P | 0.61 | 0.39 | N | 0.33 | 0.67 | P | 0.40 | 0.60 | P |
| G475D | Non-RRDR | 1.00 | 0.00 | N | 0.37 | 0.63 | P | 0.23 | 0.77 | P | 0.57 | 0.43 | N | 0.35 | 0.65 | P | 0.40 | 0.60 | P |
| G475E | Non-RRDR | 1.00 | 0.00 | N | 0.37 | 0.63 | P | 0.19 | 0.81 | P | 0.56 | 0.44 | N | 0.33 | 0.67 | P | 0.40 | 0.60 | P |
| G475F | Non-RRDR | 1.00 | 0.00 | N | 0.67 | 0.33 | N | 0.29 | 0.71 | P | 0.67 | 0.33 | N | 0.37 | 0.63 | P | 0.60 | 0.40 | N |
| G475H | Non-RRDR | 1.00 | 0.00 | N | 0.37 | 0.63 | P | 0.00 | 1.00 | P | 0.59 | 0.41 | N | 0.34 | 0.66 | P | 0.40 | 0.60 | P |
| G475I | Non-RRDR | 1.00 | 0.00 | N | 0.67 | 0.33 | N | 0.28 | 0.72 | P | 0.67 | 0.33 | N | 0.37 | 0.63 | P | 0.60 | 0.40 | N |
| G475K | Non-RRDR | 1.00 | 0.00 | N | 0.37 | 0.63 | P | 0.18 | 0.82 | P | 0.60 | 0.40 | N | 0.35 | 0.65 | P | 0.40 | 0.60 | P |
| G475L | Non-RRDR | 1.00 | 0.00 | N | 0.67 | 0.33 | N | 0.18 | 0.82 | P | 0.66 | 0.34 | N | 0.36 | 0.64 | P | 0.60 | 0.40 | N |
| G475M | Non-RRDR | 1.00 | 0.00 | N | 0.38 | 0.62 | P | 0.23 | 0.77 | P | 0.66 | 0.34 | N | 0.35 | 0.65 | P | 0.40 | 0.60 | P |
| G475N | Non-RRDR | 1.00 | 0.00 | N | 0.37 | 0.63 | P | 0.18 | 0.82 | P | 0.58 | 0.42 | N | 0.35 | 0.65 | P | 0.40 | 0.60 | P |
| G475P | Non-RRDR | 1.00 | 0.00 | N | 0.37 | 0.63 | P | 0.10 | 0.90 | P | 0.59 | 0.41 | N | 0.34 | 0.66 | P | 0.40 | 0.60 | P |
| G475Q | Non-RRDR | 1.00 | 0.00 | N | 0.37 | 0.63 | P | 0.05 | 0.95 | P | 0.58 | 0.42 | N | 0.34 | 0.66 | P | 0.40 | 0.60 | P |
| G475R | Non-RRDR | 1.00 | 0.00 | N | 0.37 | 0.63 | P | 0.11 | 0.89 | P | 0.59 | 0.41 | N | 0.34 | 0.66 | P | 0.40 | 0.60 | P |
| G475S | Non-RRDR | 1.00 | 0.00 | N | 0.37 | 0.63 | P | 0.14 | 0.86 | P | 0.61 | 0.39 | N | 0.36 | 0.64 | P | 0.40 | 0.60 | P |
| G475T | Non-RRDR | 1.00 | 0.00 | N | 0.38 | 0.62 | P | 0.24 | 0.76 | P | 0.64 | 0.36 | N | 0.37 | 0.63 | P | 0.40 | 0.60 | P |
| G475V | Non-RRDR | 1.00 | 0.00 | N | 0.37 | 0.63 | P | 0.17 | 0.83 | P | 0.62 | 0.38 | N | 0.33 | 0.67 | P | 0.40 | 0.60 | P |
| G475W | Non-RRDR | 1.00 | 0.00 | N | 0.37 | 0.63 | P | 0.09 | 0.91 | P | 0.63 | 0.37 | N | 0.35 | 0.65 | P | 0.40 | 0.60 | P |
| G475Y | Non-RRDR | 1.00 | 0.00 | N | 0.67 | 0.33 | N | 0.01 | 0.99 | P | 0.68 | 0.32 | N | 0.38 | 0.62 | P | 0.60 | 0.40 | N |
| P589A | Non-RRDR | 1.00 | 0.00 | N | 0.74 | 0.26 | N | 0.17 | 0.83 | P | 0.88 | 0.12 | N | 0.37 | 0.63 | P | 0.60 | 0.40 | N |
| P589C | Non-RRDR | 1.00 | 0.00 | N | 0.75 | 0.25 | N | 0.35 | 0.65 | P | 0.89 | 0.11 | N | 0.38 | 0.62 | P | 0.60 | 0.40 | N |
| P589D | Non-RRDR | 1.00 | 0.00 | N | 0.71 | 0.29 | N | 0.34 | 0.66 | P | 0.85 | 0.15 | N | 0.35 | 0.65 | P | 0.60 | 0.40 | N |
| P589E | Non-RRDR | 1.00 | 0.00 | N | 0.71 | 0.29 | N | 0.23 | 0.77 | P | 0.84 | 0.16 | N | 0.34 | 0.66 | P | 0.60 | 0.40 | N |
| P589F | Non-RRDR | 1.00 | 0.00 | N | 0.73 | 0.27 | N | 0.26 | 0.74 | P | 0.92 | 0.08 | N | 0.45 | 0.55 | P | 0.60 | 0.40 | N |
| P589G | Non-RRDR | 1.00 | 0.00 | N | 0.73 | 0.27 | N | 0.04 | 0.96 | P | 0.86 | 0.14 | N | 0.35 | 0.65 | P | 0.60 | 0.40 | N |
| P589H | Non-RRDR | 1.00 | 0.00 | N | 0.73 | 0.27 | N | 0.24 | 0.76 | P | 0.87 | 0.13 | N | 0.35 | 0.65 | P | 0.60 | 0.40 | N |
| P589I | Non-RRDR | 1.00 | 0.00 | N | 0.73 | 0.27 | N | 0.24 | 0.76 | P | 0.92 | 0.08 | N | 0.43 | 0.57 | P | 0.60 | 0.40 | N |
| P589K | Non-RRDR | 1.00 | 0.00 | N | 0.73 | 0.27 | N | 0.20 | 0.80 | P | 0.87 | 0.13 | N | 0.36 | 0.64 | P | 0.60 | 0.40 | N |
| P589L | Non-RRDR | 1.00 | 0.00 | N | 0.74 | 0.26 | N | 0.06 | 0.94 | P | 0.91 | 0.09 | N | 0.43 | 0.57 | P | 0.60 | 0.40 | N |
| P589M | Non-RRDR | 1.00 | 0.00 | N | 0.74 | 0.26 | N | 0.05 | 0.95 | P | 0.91 | 0.09 | N | 0.41 | 0.59 | P | 0.60 | 0.40 | N |
| P589N | Non-RRDR | 1.00 | 0.00 | N | 0.71 | 0.29 | N | 0.27 | 0.73 | P | 0.85 | 0.15 | N | 0.34 | 0.66 | P | 0.60 | 0.40 | N |
| P589Q | Non-RRDR | 1.00 | 0.00 | N | 0.71 | 0.29 | N | 0.23 | 0.77 | P | 0.85 | 0.15 | N | 0.35 | 0.65 | P | 0.60 | 0.40 | N |
| P589R | Non-RRDR | 1.00 | 0.00 | N | 0.73 | 0.27 | N | 0.16 | 0.84 | P | 0.87 | 0.13 | N | 0.35 | 0.65 | P | 0.60 | 0.40 | N |
| P589S | Non-RRDR | 1.00 | 0.00 | N | 0.73 | 0.27 | N | 0.08 | 0.92 | P | 0.87 | 0.13 | N | 0.36 | 0.64 | P | 0.60 | 0.40 | N |
| P589T | Non-RRDR | 1.00 | 0.00 | N | 0.74 | 0.26 | N | 0.21 | 0.79 | P | 0.88 | 0.12 | N | 0.38 | 0.62 | P | 0.60 | 0.40 | N |
| P589V | Non-RRDR | 1.00 | 0.00 | N | 0.74 | 0.26 | N | 0.11 | 0.89 | P | 0.91 | 0.09 | N | 0.40 | 0.60 | P | 0.60 | 0.40 | N |
| P589W | Non-RRDR | 1.00 | 0.00 | N | 0.75 | 0.25 | N | 0.46 | 0.54 | P | 0.90 | 0.10 | N | 0.42 | 0.58 | P | 0.60 | 0.40 | N |
| P589Y | Non-RRDR | 1.00 | 0.00 | N | 0.73 | 0.27 | N | 0.23 | 0.77 | P | 0.92 | 0.08 | N | 0.44 | 0.56 | P | 0.60 | 0.40 | N |
| E748A | Non-RRDR | 0.04 | 0.96 | P | 0.32 | 0.68 | P | 0.82 | 0.18 | N | 0.13 | 0.87 | P | 0.46 | 0.54 | P | 0.20 | 0.80 | P |
| E748C | Non-RRDR | 0.04 | 0.96 | P | 0.32 | 0.68 | P | 0.93 | 0.07 | N | 0.12 | 0.88 | P | 0.44 | 0.56 | P | 0.20 | 0.80 | P |
| E748D | Non-RRDR | 0.00 | 1.00 | P | 0.33 | 0.67 | P | 1.00 | 0.00 | N | 0.16 | 0.84 | P | 0.48 | 0.52 | P | 0.20 | 0.80 | P |
| E748F | Non-RRDR | 0.04 | 0.96 | P | 0.31 | 0.69 | P | 0.88 | 0.12 | N | 0.12 | 0.88 | P | 0.45 | 0.55 | P | 0.20 | 0.80 | P |
| E748G | Non-RRDR | 0.04 | 0.96 | P | 0.32 | 0.68 | P | 0.34 | 0.66 | P | 0.13 | 0.87 | P | 0.42 | 0.58 | P | 0.00 | 1.00 | P |
| E748H | Non-RRDR | 0.04 | 0.96 | P | 0.32 | 0.68 | P | 0.32 | 0.68 | P | 0.13 | 0.87 | P | 0.44 | 0.56 | P | 0.00 | 1.00 | P |
| E748I | Non-RRDR | 0.04 | 0.96 | P | 0.31 | 0.69 | P | 0.53 | 0.47 | N | 0.12 | 0.88 | P | 0.46 | 0.54 | P | 0.20 | 0.80 | P |
| E748K | Non-RRDR | 1.00 | 0.00 | N | 0.32 | 0.68 | P | 1.00 | 0.00 | N | 0.18 | 0.82 | P | 0.54 | 0.46 | N | 0.60 | 0.40 | N |
| E748L | Non-RRDR | 0.00 | 1.00 | P | 0.31 | 0.69 | P | 0.83 | 0.17 | N | 0.11 | 0.89 | P | 0.45 | 0.55 | P | 0.20 | 0.80 | P |
| E748M | Non-RRDR | 0.00 | 1.00 | P | 0.31 | 0.69 | P | 0.79 | 0.21 | N | 0.11 | 0.89 | P | 0.45 | 0.55 | P | 0.20 | 0.80 | P |
| E748N | Non-RRDR | 0.04 | 0.96 | P | 0.32 | 0.68 | P | 0.96 | 0.04 | N | 0.13 | 0.87 | P | 0.44 | 0.56 | P | 0.20 | 0.80 | P |
| E748P | Non-RRDR | 0.04 | 0.96 | P | 0.32 | 0.68 | P | 0.94 | 0.06 | N | 0.13 | 0.87 | P | 0.43 | 0.57 | P | 0.20 | 0.80 | P |
| E748Q | Non-RRDR | 0.00 | 1.00 | P | 0.32 | 0.68 | P | 1.00 | 0.00 | N | 0.17 | 0.83 | P | 0.52 | 0.48 | N | 0.40 | 0.60 | P |
| E748R | Non-RRDR | 0.00 | 1.00 | P | 0.32 | 0.68 | P | 0.99 | 0.01 | N | 0.15 | 0.85 | P | 0.49 | 0.51 | P | 0.20 | 0.80 | P |
| E748S | Non-RRDR | 0.04 | 0.96 | P | 0.32 | 0.68 | P | 0.97 | 0.03 | N | 0.14 | 0.86 | P | 0.46 | 0.54 | P | 0.20 | 0.80 | P |
| E748T | Non-RRDR | 0.04 | 0.96 | P | 0.32 | 0.68 | P | 0.92 | 0.08 | N | 0.13 | 0.87 | P | 0.46 | 0.54 | P | 0.20 | 0.80 | P |
| E748V | Non-RRDR | 0.00 | 1.00 | P | 0.31 | 0.69 | P | 0.83 | 0.17 | N | 0.12 | 0.88 | P | 0.46 | 0.54 | P | 0.20 | 0.80 | P |
| E748W | Non-RRDR | 0.00 | 1.00 | P | 0.31 | 0.69 | P | 0.88 | 0.12 | N | 0.12 | 0.88 | P | 0.45 | 0.55 | P | 0.20 | 0.80 | P |
| E748Y | Non-RRDR | 0.00 | 1.00 | P | 0.31 | 0.69 | P | 0.87 | 0.13 | N | 0.12 | 0.88 | P | 0.45 | 0.55 | P | 0.20 | 0.80 | P |
| A760C | Non-RRDR | 0.33 | 0.67 | P | 0.62 | 0.38 | N | 0.99 | 0.01 | N | 0.57 | 0.43 | N | 0.66 | 0.34 | N | 0.80 | 0.20 | N |
| A760D | Non-RRDR | 0.92 | 0.08 | N | 0.63 | 0.37 | N | 1.00 | 0.00 | N | 0.63 | 0.37 | N | 0.69 | 0.31 | N | 1.00 | 0.00 | N |
| A760E | Non-RRDR | 0.33 | 0.67 | P | 0.63 | 0.37 | N | 1.00 | 0.00 | N | 0.64 | 0.36 | N | 0.70 | 0.30 | N | 0.80 | 0.20 | N |
| A760F | Non-RRDR | 0.33 | 0.67 | P | 0.62 | 0.38 | N | 0.98 | 0.02 | N | 0.61 | 0.39 | N | 0.70 | 0.30 | N | 0.80 | 0.20 | N |
| A760G | Non-RRDR | 0.33 | 0.67 | P | 0.62 | 0.38 | N | 0.99 | 0.01 | N | 0.58 | 0.42 | N | 0.64 | 0.36 | N | 0.80 | 0.20 | N |
| A760H | Non-RRDR | 0.92 | 0.08 | N | 0.62 | 0.38 | N | 0.99 | 0.01 | N | 0.59 | 0.41 | N | 0.67 | 0.33 | N | 1.00 | 0.00 | N |
| A760I | Non-RRDR | 0.33 | 0.67 | P | 0.62 | 0.38 | N | 0.98 | 0.02 | N | 0.61 | 0.39 | N | 0.70 | 0.30 | N | 0.80 | 0.20 | N |
| A760K | Non-RRDR | 0.92 | 0.08 | N | 0.63 | 0.37 | N | 0.99 | 0.01 | N | 0.61 | 0.39 | N | 0.68 | 0.32 | N | 1.00 | 0.00 | N |
| A760L | Non-RRDR | 0.33 | 0.67 | P | 0.62 | 0.38 | N | 0.98 | 0.02 | N | 0.59 | 0.41 | N | 0.69 | 0.31 | N | 0.80 | 0.20 | N |
| A760M | Non-RRDR | 0.33 | 0.67 | P | 0.62 | 0.38 | N | 0.97 | 0.03 | N | 0.57 | 0.43 | N | 0.66 | 0.34 | N | 0.80 | 0.20 | N |
| A760N | Non-RRDR | 0.33 | 0.67 | P | 0.33 | 0.67 | P | 0.99 | 0.01 | N | 0.59 | 0.41 | N | 0.64 | 0.36 | N | 0.60 | 0.40 | N |
| A760P | Non-RRDR | 0.92 | 0.08 | N | 0.37 | 0.63 | P | 1.00 | 0.00 | N | 0.92 | 0.08 | N | 0.88 | 0.12 | N | 0.80 | 0.20 | N |
| A760Q | Non-RRDR | 0.33 | 0.67 | P | 0.63 | 0.37 | N | 1.00 | 0.00 | N | 0.61 | 0.39 | N | 0.67 | 0.33 | N | 0.80 | 0.20 | N |
| A760R | Non-RRDR | 0.33 | 0.67 | P | 0.62 | 0.38 | N | 0.98 | 0.02 | N | 0.58 | 0.42 | N | 0.65 | 0.35 | N | 0.80 | 0.20 | N |
| A760S | Non-RRDR | 0.92 | 0.08 | N | 0.64 | 0.36 | N | 1.00 | 0.00 | N | 0.71 | 0.29 | N | 0.77 | 0.23 | N | 1.00 | 0.00 | N |
| A760T | Non-RRDR | 0.33 | 0.67 | P | 0.62 | 0.38 | N | 1.00 | 0.00 | N | 0.61 | 0.39 | N | 0.69 | 0.31 | N | 0.80 | 0.20 | N |
| A760V | Non-RRDR | 0.33 | 0.67 | P | 0.62 | 0.38 | N | 0.83 | 0.17 | N | 0.58 | 0.42 | N | 0.66 | 0.34 | N | 0.80 | 0.20 | N |
| A760W | Non-RRDR | 0.33 | 0.67 | P | 0.62 | 0.38 | N | 0.90 | 0.10 | N | 0.57 | 0.43 | N | 0.65 | 0.35 | N | 0.80 | 0.20 | N |
| A760Y | Non-RRDR | 0.33 | 0.67 | P | 0.62 | 0.38 | N | 0.97 | 0.03 | N | 0.62 | 0.38 | N | 0.71 | 0.29 | N | 0.80 | 0.20 | N |
| E959A | Non-RRDR | 0.92 | 0.08 | N | 0.80 | 0.20 | N | 1.00 | 0.00 | N | 0.79 | 0.21 | N | 0.84 | 0.16 | N | 1.00 | 0.00 | N |
| E959C | Non-RRDR | 0.92 | 0.08 | N | 0.80 | 0.20 | N | 1.00 | 0.00 | N | 0.82 | 0.18 | N | 0.83 | 0.17 | N | 1.00 | 0.00 | N |
| E959D | Non-RRDR | 0.92 | 0.08 | N | 0.76 | 0.24 | N | 1.00 | 0.00 | N | 0.71 | 0.29 | N | 0.86 | 0.14 | N | 1.00 | 0.00 | N |
| E959F | Non-RRDR | 0.92 | 0.08 | N | 0.85 | 0.15 | N | 1.00 | 0.00 | N | 0.82 | 0.18 | N | 0.85 | 0.15 | N | 1.00 | 0.00 | N |
| E959G | Non-RRDR | 0.92 | 0.08 | N | 0.48 | 0.52 | P | 1.00 | 0.00 | N | 0.67 | 0.33 | N | 0.89 | 0.11 | N | 0.80 | 0.20 | N |
| E959H | Non-RRDR | 0.92 | 0.08 | N | 0.81 | 0.19 | N | 1.00 | 0.00 | N | 0.79 | 0.21 | N | 0.85 | 0.15 | N | 1.00 | 0.00 | N |
| E959I | Non-RRDR | 0.92 | 0.08 | N | 0.85 | 0.15 | N | 1.00 | 0.00 | N | 0.82 | 0.18 | N | 0.85 | 0.15 | N | 1.00 | 0.00 | N |
| E959K | Non-RRDR | 0.92 | 0.08 | N | 0.82 | 0.18 | N | 1.00 | 0.00 | N | 0.78 | 0.22 | N | 0.86 | 0.14 | N | 1.00 | 0.00 | N |
| E959L | Non-RRDR | 0.92 | 0.08 | N | 0.85 | 0.15 | N | 1.00 | 0.00 | N | 0.82 | 0.18 | N | 0.85 | 0.15 | N | 1.00 | 0.00 | N |
| E959M | Non-RRDR | 0.92 | 0.08 | N | 0.86 | 0.14 | N | 1.00 | 0.00 | N | 0.82 | 0.18 | N | 0.86 | 0.14 | N | 1.00 | 0.00 | N |
| E959N | Non-RRDR | 0.92 | 0.08 | N | 0.81 | 0.19 | N | 1.00 | 0.00 | N | 0.79 | 0.21 | N | 0.85 | 0.15 | N | 1.00 | 0.00 | N |
| E959P | Non-RRDR | 0.00 | 1.00 | P | 0.79 | 0.21 | N | 1.00 | 0.00 | N | 0.82 | 0.18 | N | 0.83 | 0.17 | N | 0.80 | 0.20 | N |
| E959Q | Non-RRDR | 0.92 | 0.08 | N | 0.81 | 0.19 | N | 1.00 | 0.00 | N | 0.78 | 0.22 | N | 0.86 | 0.14 | N | 1.00 | 0.00 | N |
| E959R | Non-RRDR | 0.92 | 0.08 | N | 0.81 | 0.19 | N | 1.00 | 0.00 | N | 0.79 | 0.21 | N | 0.85 | 0.15 | N | 1.00 | 0.00 | N |
| E959S | Non-RRDR | 0.92 | 0.08 | N | 0.83 | 0.17 | N | 1.00 | 0.00 | N | 0.79 | 0.21 | N | 0.86 | 0.14 | N | 1.00 | 0.00 | N |
| E959T | Non-RRDR | 0.92 | 0.08 | N | 0.80 | 0.20 | N | 1.00 | 0.00 | N | 0.79 | 0.21 | N | 0.84 | 0.16 | N | 1.00 | 0.00 | N |
| E959V | Non-RRDR | 0.92 | 0.08 | N | 0.86 | 0.14 | N | 1.00 | 0.00 | N | 0.82 | 0.18 | N | 0.86 | 0.14 | N | 1.00 | 0.00 | N |
| E959W | Non-RRDR | 0.92 | 0.08 | N | 0.82 | 0.18 | N | 1.00 | 0.00 | N | 0.83 | 0.17 | N | 0.84 | 0.16 | N | 1.00 | 0.00 | N |
| E959Y | Non-RRDR | 0.92 | 0.08 | N | 0.86 | 0.14 | N | 1.00 | 0.00 | N | 0.82 | 0.18 | N | 0.85 | 0.15 | N | 1.00 | 0.00 | N |
| D993A | Non-RRDR | 0.00 | 1.00 | P | 0.29 | 0.71 | P | 1.00 | 0.00 | N | 0.06 | 0.94 | P | 0.84 | 0.16 | N | 0.40 | 0.60 | P |
| D993C | Non-RRDR | 0.00 | 1.00 | P | 0.29 | 0.71 | P | 1.00 | 0.00 | N | 0.06 | 0.94 | P | 0.84 | 0.16 | N | 0.40 | 0.60 | P |
| D993E | Non-RRDR | 0.00 | 1.00 | P | 0.30 | 0.70 | P | 1.00 | 0.00 | N | 0.08 | 0.92 | P | 0.91 | 0.09 | N | 0.40 | 0.60 | P |
| D993F | Non-RRDR | 0.00 | 1.00 | P | 0.30 | 0.70 | P | 0.99 | 0.01 | N | 0.06 | 0.94 | P | 0.85 | 0.15 | N | 0.40 | 0.60 | P |
| D993G | Non-RRDR | 0.00 | 1.00 | P | 0.29 | 0.71 | P | 1.00 | 0.00 | N | 0.06 | 0.94 | P | 0.84 | 0.16 | N | 0.40 | 0.60 | P |
| D993H | Non-RRDR | 0.67 | 0.33 | N | 0.29 | 0.71 | P | 1.00 | 0.00 | N | 0.06 | 0.94 | P | 0.84 | 0.16 | N | 0.60 | 0.40 | N |
| D993I | Non-RRDR | 0.00 | 1.00 | P | 0.30 | 0.70 | P | 1.00 | 0.00 | N | 0.06 | 0.94 | P | 0.87 | 0.13 | N | 0.40 | 0.60 | P |
| D993K | Non-RRDR | 0.00 | 1.00 | P | 0.30 | 0.70 | P | 1.00 | 0.00 | N | 0.07 | 0.93 | P | 0.89 | 0.11 | N | 0.40 | 0.60 | P |
| D993L | Non-RRDR | 0.67 | 0.33 | N | 0.30 | 0.70 | P | 1.00 | 0.00 | N | 0.06 | 0.94 | P | 0.87 | 0.13 | N | 0.60 | 0.40 | N |
| D993M | Non-RRDR | 0.67 | 0.33 | N | 0.30 | 0.70 | P | 1.00 | 0.00 | N | 0.06 | 0.94 | P | 0.88 | 0.12 | N | 0.60 | 0.40 | N |
| D993N | Non-RRDR | 0.00 | 1.00 | P | 0.30 | 0.70 | P | 1.00 | 0.00 | N | 0.07 | 0.93 | P | 0.87 | 0.13 | N | 0.40 | 0.60 | P |
| D993P | Non-RRDR | 0.00 | 1.00 | P | 0.29 | 0.71 | P | 1.00 | 0.00 | N | 0.06 | 0.94 | P | 0.84 | 0.16 | N | 0.40 | 0.60 | P |
| D993Q | Non-RRDR | 0.00 | 1.00 | P | 0.30 | 0.70 | P | 1.00 | 0.00 | N | 0.07 | 0.93 | P | 0.89 | 0.11 | N | 0.40 | 0.60 | P |
| D993R | Non-RRDR | 0.00 | 1.00 | P | 0.30 | 0.70 | P | 0.99 | 0.01 | N | 0.07 | 0.93 | P | 0.88 | 0.12 | N | 0.40 | 0.60 | P |
| D993S | Non-RRDR | 0.00 | 1.00 | P | 0.29 | 0.71 | P | 1.00 | 0.00 | N | 0.06 | 0.94 | P | 0.85 | 0.15 | N | 0.40 | 0.60 | P |
| D993T | Non-RRDR | 0.00 | 1.00 | P | 0.29 | 0.71 | P | 1.00 | 0.00 | N | 0.06 | 0.94 | P | 0.85 | 0.15 | N | 0.40 | 0.60 | P |
| D993V | Non-RRDR | 0.00 | 1.00 | P | 0.30 | 0.70 | P | 1.00 | 0.00 | N | 0.06 | 0.94 | P | 0.88 | 0.12 | N | 0.40 | 0.60 | P |
| D993W | Non-RRDR | 0.67 | 0.33 | N | 0.30 | 0.70 | P | 1.00 | 0.00 | N | 0.06 | 0.94 | P | 0.85 | 0.15 | N | 0.60 | 0.40 | N |
| D993Y | Non-RRDR | 0.00 | 1.00 | P | 0.30 | 0.70 | P | 1.00 | 0.00 | N | 0.06 | 0.94 | P | 0.85 | 0.15 | N | 0.40 | 0.60 | P |
| A1075C | Non-RRDR | 0.92 | 0.08 | N | 0.65 | 0.35 | N | 0.99 | 0.01 | N | 0.43 | 0.57 | P | 0.64 | 0.36 | N | 0.80 | 0.20 | N |
| A1075D | Non-RRDR | 0.92 | 0.08 | N | 0.65 | 0.35 | N | 0.98 | 0.02 | N | 0.45 | 0.55 | P | 0.64 | 0.36 | N | 0.80 | 0.20 | N |
| A1075E | Non-RRDR | 0.00 | 1.00 | P | 0.65 | 0.35 | N | 0.77 | 0.23 | N | 0.46 | 0.54 | P | 0.66 | 0.34 | N | 0.60 | 0.40 | N |
| A1075F | Non-RRDR | 0.33 | 0.67 | P | 0.40 | 0.60 | P | 0.96 | 0.04 | N | 0.45 | 0.55 | P | 0.66 | 0.34 | N | 0.40 | 0.60 | P |
| A1075G | Non-RRDR | 0.92 | 0.08 | N | 0.65 | 0.35 | N | 0.98 | 0.02 | N | 0.42 | 0.58 | P | 0.63 | 0.37 | N | 0.80 | 0.20 | N |
| A1075H | Non-RRDR | 0.92 | 0.08 | N | 0.38 | 0.62 | P | 0.98 | 0.02 | N | 0.42 | 0.58 | P | 0.61 | 0.39 | N | 0.60 | 0.40 | N |
| A1075I | Non-RRDR | 0.33 | 0.67 | P | 0.40 | 0.60 | P | 0.97 | 0.03 | N | 0.45 | 0.55 | P | 0.65 | 0.35 | N | 0.40 | 0.60 | P |
| A1075K | Non-RRDR | 0.92 | 0.08 | N | 0.65 | 0.35 | N | 0.97 | 0.03 | N | 0.43 | 0.57 | P | 0.62 | 0.38 | N | 0.80 | 0.20 | N |
| A1075L | Non-RRDR | 0.33 | 0.67 | P | 0.40 | 0.60 | P | 0.98 | 0.02 | N | 0.45 | 0.55 | P | 0.65 | 0.35 | N | 0.40 | 0.60 | P |
| A1075M | Non-RRDR | 0.92 | 0.08 | N | 0.40 | 0.60 | P | 0.97 | 0.03 | N | 0.43 | 0.57 | P | 0.63 | 0.37 | N | 0.60 | 0.40 | N |
| A1075N | Non-RRDR | 0.92 | 0.08 | N | 0.37 | 0.63 | P | 0.92 | 0.08 | N | 0.42 | 0.58 | P | 0.60 | 0.40 | N | 0.60 | 0.40 | N |
| A1075P | Non-RRDR | 0.92 | 0.08 | N | 0.38 | 0.62 | P | 0.98 | 0.02 | N | 0.43 | 0.57 | P | 0.63 | 0.37 | N | 0.60 | 0.40 | N |
| A1075Q | Non-RRDR | 0.92 | 0.08 | N | 0.65 | 0.35 | N | 0.94 | 0.06 | N | 0.43 | 0.57 | P | 0.62 | 0.38 | N | 0.80 | 0.20 | N |
| A1075R | Non-RRDR | 0.92 | 0.08 | N | 0.66 | 0.34 | N | 0.97 | 0.03 | N | 0.43 | 0.57 | P | 0.63 | 0.37 | N | 0.80 | 0.20 | N |
| A1075S | Non-RRDR | 0.92 | 0.08 | N | 0.65 | 0.35 | N | 0.99 | 0.01 | N | 0.44 | 0.56 | P | 0.66 | 0.34 | N | 0.80 | 0.20 | N |
| A1075T | Non-RRDR | 0.92 | 0.08 | N | 0.38 | 0.62 | P | 0.80 | 0.20 | N | 0.42 | 0.58 | P | 0.62 | 0.38 | N | 0.60 | 0.40 | N |
| A1075V | Non-RRDR | 0.92 | 0.08 | N | 0.40 | 0.60 | P | 0.84 | 0.16 | N | 0.43 | 0.57 | P | 0.63 | 0.37 | N | 0.60 | 0.40 | N |
| A1075W | Non-RRDR | 0.33 | 0.67 | P | 0.40 | 0.60 | P | 0.96 | 0.04 | N | 0.42 | 0.58 | P | 0.63 | 0.37 | N | 0.40 | 0.60 | P |
| A1075Y | Non-RRDR | 0.33 | 0.67 | P | 0.40 | 0.60 | P | 0.98 | 0.02 | N | 0.45 | 0.55 | P | 0.66 | 0.34 | N | 0.40 | 0.60 | P |
| V169A | RRDR | 0.04 | 0.96 | P | 0.00 | 1.00 | P | 0.00 | 1.00 | P | 0.00 | 1.00 | P | 0.13 | 0.87 | P | 0.00 | 1.00 | P |
| V169C | RRDR | 0.04 | 0.96 | P | 0.00 | 1.00 | P | 0.00 | 1.00 | P | 0.00 | 1.00 | P | 0.18 | 0.82 | P | 0.00 | 1.00 | P |
| V169D | RRDR | 0.04 | 0.96 | P | 0.00 | 1.00 | P | 0.00 | 1.00 | P | 0.00 | 1.00 | P | 0.09 | 0.91 | P | 0.00 | 1.00 | P |
| V169E | RRDR | 0.04 | 0.96 | P | 0.00 | 1.00 | P | 0.00 | 1.00 | P | 0.00 | 1.00 | P | 0.10 | 0.90 | P | 0.00 | 1.00 | P |
| V169F | RRDR | 0.00 | 1.00 | P | 0.00 | 1.00 | P | 0.00 | 1.00 | P | 0.00 | 1.00 | P | 0.23 | 0.77 | P | 0.00 | 1.00 | P |
| V169G | RRDR | 0.04 | 0.96 | P | 0.00 | 1.00 | P | 0.00 | 1.00 | P | 0.00 | 1.00 | P | 0.09 | 0.91 | P | 0.00 | 1.00 | P |
| V169H | RRDR | 0.04 | 0.96 | P | 0.00 | 1.00 | P | 0.00 | 1.00 | P | 0.00 | 1.00 | P | 0.09 | 0.91 | P | 0.00 | 1.00 | P |
| V169I | RRDR | 1.00 | 0.00 | N | 0.00 | 1.00 | P | 0.01 | 0.99 | P | 0.01 | 0.99 | P | 0.48 | 0.52 | P | 0.20 | 0.80 | P |
| V169K | RRDR | 0.04 | 0.96 | P | 0.00 | 1.00 | P | 0.00 | 1.00 | P | 0.00 | 1.00 | P | 0.11 | 0.89 | P | 0.00 | 1.00 | P |
| V169L | RRDR | 0.04 | 0.96 | P | 0.00 | 1.00 | P | 0.00 | 1.00 | P | 0.00 | 1.00 | P | 0.26 | 0.74 | P | 0.00 | 1.00 | P |
| V169M | RRDR | 0.04 | 0.96 | P | 0.00 | 1.00 | P | 0.00 | 1.00 | P | 0.00 | 1.00 | P | 0.22 | 0.78 | P | 0.00 | 1.00 | P |
| V169N | RRDR | 0.04 | 0.96 | P | 0.00 | 1.00 | P | 0.00 | 1.00 | P | 0.00 | 1.00 | P | 0.09 | 0.91 | P | 0.00 | 1.00 | P |
| V169P | RRDR | 0.04 | 0.96 | P | 0.00 | 1.00 | P | 0.00 | 1.00 | P | 0.00 | 1.00 | P | 0.12 | 0.88 | P | 0.00 | 1.00 | P |
| V169Q | RRDR | 0.04 | 0.96 | P | 0.00 | 1.00 | P | 0.00 | 1.00 | P | 0.00 | 1.00 | P | 0.11 | 0.89 | P | 0.00 | 1.00 | P |
| V169R | RRDR | 0.00 | 1.00 | P | 0.00 | 1.00 | P | 0.00 | 1.00 | P | 0.00 | 1.00 | P | 0.10 | 0.90 | P | 0.00 | 1.00 | P |
| V169S | RRDR | 0.04 | 0.96 | P | 0.00 | 1.00 | P | 0.00 | 1.00 | P | 0.00 | 1.00 | P | 0.11 | 0.89 | P | 0.00 | 1.00 | P |
| V169T | RRDR | 0.04 | 0.96 | P | 0.00 | 1.00 | P | 0.00 | 1.00 | P | 0.00 | 1.00 | P | 0.15 | 0.85 | P | 0.00 | 1.00 | P |
| V169W | RRDR | 0.00 | 1.00 | P | 0.00 | 1.00 | P | 0.00 | 1.00 | P | 0.00 | 1.00 | P | 0.18 | 0.82 | P | 0.00 | 1.00 | P |
| V169Y | RRDR | 0.00 | 1.00 | P | 0.00 | 1.00 | P | 0.00 | 1.00 | P | 0.00 | 1.00 | P | 0.23 | 0.77 | P | 0.00 | 1.00 | P |
| L430A | RRDR | 0.04 | 0.96 | P | 0.00 | 1.00 | P | 0.00 | 1.00 | P | 0.03 | 0.97 | P | 0.14 | 0.86 | P | 0.00 | 1.00 | P |
| L430C | RRDR | 0.04 | 0.96 | P | 0.31 | 0.69 | P | 0.00 | 1.00 | P | 0.09 | 0.91 | P | 0.20 | 0.80 | P | 0.00 | 1.00 | P |
| L430D | RRDR | 0.00 | 1.00 | P | 0.00 | 1.00 | P | 0.00 | 1.00 | P | 0.01 | 0.99 | P | 0.10 | 0.90 | P | 0.00 | 1.00 | P |
| L430E | RRDR | 0.00 | 1.00 | P | 0.00 | 1.00 | P | 0.00 | 1.00 | P | 0.01 | 0.99 | P | 0.09 | 0.91 | P | 0.00 | 1.00 | P |
| L430F | RRDR | 0.00 | 1.00 | P | 0.39 | 0.61 | P | 0.00 | 1.00 | P | 0.12 | 0.88 | P | 0.25 | 0.75 | P | 0.00 | 1.00 | P |
| L430G | RRDR | 0.00 | 1.00 | P | 0.00 | 1.00 | P | 0.00 | 1.00 | P | 0.02 | 0.98 | P | 0.10 | 0.90 | P | 0.00 | 1.00 | P |
| L430H | RRDR | 0.00 | 1.00 | P | 0.00 | 1.00 | P | 0.00 | 1.00 | P | 0.02 | 0.98 | P | 0.11 | 0.89 | P | 0.00 | 1.00 | P |
| L430I | RRDR | 0.75 | 0.25 | N | 0.38 | 0.62 | P | 0.00 | 1.00 | P | 0.12 | 0.88 | P | 0.26 | 0.74 | P | 0.20 | 0.80 | P |
| L430K | RRDR | 0.04 | 0.96 | P | 0.00 | 1.00 | P | 0.00 | 1.00 | P | 0.03 | 0.97 | P | 0.12 | 0.88 | P | 0.00 | 1.00 | P |
| L430M | RRDR | 0.04 | 0.96 | P | 0.38 | 0.62 | P | 0.00 | 1.00 | P | 0.11 | 0.89 | P | 0.25 | 0.75 | P | 0.00 | 1.00 | P |
| L430N | RRDR | 0.00 | 1.00 | P | 0.00 | 1.00 | P | 0.00 | 1.00 | P | 0.01 | 0.99 | P | 0.10 | 0.90 | P | 0.00 | 1.00 | P |
| L430P | RRDR | 0.00 | 1.00 | P | 0.00 | 1.00 | P | 0.00 | 1.00 | P | 0.02 | 0.98 | P | 0.10 | 0.90 | P | 0.00 | 1.00 | P |
| L430Q | RRDR | 0.04 | 0.96 | P | 0.00 | 1.00 | P | 0.00 | 1.00 | P | 0.02 | 0.98 | P | 0.12 | 0.88 | P | 0.00 | 1.00 | P |
| L430R | RRDR | 0.04 | 0.96 | P | 0.00 | 1.00 | P | 0.00 | 1.00 | P | 0.03 | 0.97 | P | 0.12 | 0.88 | P | 0.00 | 1.00 | P |
| L430S | RRDR | 0.04 | 0.96 | P | 0.00 | 1.00 | P | 0.00 | 1.00 | P | 0.03 | 0.97 | P | 0.13 | 0.87 | P | 0.00 | 1.00 | P |
| L430T | RRDR | 0.04 | 0.96 | P | 0.00 | 1.00 | P | 0.00 | 1.00 | P | 0.03 | 0.97 | P | 0.13 | 0.87 | P | 0.00 | 1.00 | P |
| L430V | RRDR | 0.75 | 0.25 | N | 0.36 | 0.64 | P | 0.00 | 1.00 | P | 0.11 | 0.89 | P | 0.23 | 0.77 | P | 0.20 | 0.80 | P |
| L430W | RRDR | 0.75 | 0.25 | N | 0.32 | 0.68 | P | 0.00 | 1.00 | P | 0.10 | 0.90 | P | 0.21 | 0.79 | P | 0.20 | 0.80 | P |
| L430Y | RRDR | 0.75 | 0.25 | N | 0.38 | 0.62 | P | 0.00 | 1.00 | P | 0.12 | 0.88 | P | 0.24 | 0.76 | P | 0.20 | 0.80 | P |
| G485A | RRDR | 0.00 | 1.00 | P | 0.00 | 1.00 | P | 0.00 | 1.00 | P | 0.00 | 1.00 | P | 0.29 | 0.71 | P | 0.00 | 1.00 | P |
| G485C | RRDR | 0.00 | 1.00 | P | 0.00 | 1.00 | P | 0.00 | 1.00 | P | 0.00 | 1.00 | P | 0.24 | 0.76 | P | 0.00 | 1.00 | P |
| G485D | RRDR | 0.00 | 1.00 | P | 0.00 | 1.00 | P | 0.00 | 1.00 | P | 0.00 | 1.00 | P | 0.32 | 0.68 | P | 0.00 | 1.00 | P |
| G485E | RRDR | 0.00 | 1.00 | P | 0.00 | 1.00 | P | 0.00 | 1.00 | P | 0.00 | 1.00 | P | 0.29 | 0.71 | P | 0.00 | 1.00 | P |
| G485F | RRDR | 0.00 | 1.00 | P | 0.00 | 1.00 | P | 0.00 | 1.00 | P | 0.00 | 1.00 | P | 0.26 | 0.74 | P | 0.00 | 1.00 | P |
| G485H | RRDR | 0.00 | 1.00 | P | 0.00 | 1.00 | P | 0.00 | 1.00 | P | 0.00 | 1.00 | P | 0.27 | 0.73 | P | 0.00 | 1.00 | P |
| G485I | RRDR | 0.00 | 1.00 | P | 0.00 | 1.00 | P | 0.00 | 1.00 | P | 0.00 | 1.00 | P | 0.24 | 0.76 | P | 0.00 | 1.00 | P |
| G485K | RRDR | 0.00 | 1.00 | P | 0.00 | 1.00 | P | 0.00 | 1.00 | P | 0.00 | 1.00 | P | 0.27 | 0.73 | P | 0.00 | 1.00 | P |
| G485L | RRDR | 0.00 | 1.00 | P | 0.00 | 1.00 | P | 0.00 | 1.00 | P | 0.00 | 1.00 | P | 0.24 | 0.76 | P | 0.00 | 1.00 | P |
| G485M | RRDR | 0.00 | 1.00 | P | 0.00 | 1.00 | P | 0.00 | 1.00 | P | 0.00 | 1.00 | P | 0.25 | 0.75 | P | 0.00 | 1.00 | P |
| G485N | RRDR | 0.00 | 1.00 | P | 0.00 | 1.00 | P | 0.00 | 1.00 | P | 0.00 | 1.00 | P | 0.30 | 0.70 | P | 0.00 | 1.00 | P |
| G485P | RRDR | 0.00 | 1.00 | P | 0.00 | 1.00 | P | 0.00 | 1.00 | P | 0.00 | 1.00 | P | 0.27 | 0.73 | P | 0.00 | 1.00 | P |
| G485Q | RRDR | 0.00 | 1.00 | P | 0.00 | 1.00 | P | 0.00 | 1.00 | P | 0.00 | 1.00 | P | 0.28 | 0.72 | P | 0.00 | 1.00 | P |
| G485R | RRDR | 0.00 | 1.00 | P | 0.00 | 1.00 | P | 0.00 | 1.00 | P | 0.00 | 1.00 | P | 0.27 | 0.73 | P | 0.00 | 1.00 | P |
| G485S | RRDR | 0.00 | 1.00 | P | 0.00 | 1.00 | P | 0.00 | 1.00 | P | 0.00 | 1.00 | P | 0.29 | 0.71 | P | 0.00 | 1.00 | P |
| G485T | RRDR | 0.00 | 1.00 | P | 0.00 | 1.00 | P | 0.00 | 1.00 | P | 0.00 | 1.00 | P | 0.27 | 0.73 | P | 0.00 | 1.00 | P |
| G485V | RRDR | 0.00 | 1.00 | P | 0.00 | 1.00 | P | 0.00 | 1.00 | P | 0.00 | 1.00 | P | 0.23 | 0.77 | P | 0.00 | 1.00 | P |
| G485W | RRDR | 0.00 | 1.00 | P | 0.00 | 1.00 | P | 0.00 | 1.00 | P | 0.00 | 1.00 | P | 0.26 | 0.74 | P | 0.00 | 1.00 | P |
| G485Y | RRDR | 0.00 | 1.00 | P | 0.00 | 1.00 | P | 0.00 | 1.00 | P | 0.00 | 1.00 | P | 0.26 | 0.74 | P | 0.00 | 1.00 | P |
| Q608A | RRDR | 0.75 | 0.25 | N | 0.00 | 1.00 | P | 0.00 | 1.00 | P | 0.02 | 0.98 | P | 0.30 | 0.70 | P | 0.20 | 0.80 | P |
| Q608C | RRDR | 0.75 | 0.25 | N | 0.00 | 1.00 | P | 0.00 | 1.00 | P | 0.02 | 0.98 | P | 0.34 | 0.66 | P | 0.20 | 0.80 | P |
| Q608D | RRDR | 0.75 | 0.25 | N | 0.00 | 1.00 | P | 0.00 | 1.00 | P | 0.02 | 0.98 | P | 0.28 | 0.72 | P | 0.20 | 0.80 | P |
| Q608E | RRDR | 0.75 | 0.25 | N | 0.00 | 1.00 | P | 0.00 | 1.00 | P | 0.02 | 0.98 | P | 0.32 | 0.68 | P | 0.20 | 0.80 | P |
| Q608F | RRDR | 0.00 | 1.00 | P | 0.00 | 1.00 | P | 0.00 | 1.00 | P | 0.02 | 0.98 | P | 0.34 | 0.66 | P | 0.00 | 1.00 | P |
| Q608G | RRDR | 0.75 | 0.25 | N | 0.00 | 1.00 | P | 0.00 | 1.00 | P | 0.02 | 0.98 | P | 0.29 | 0.71 | P | 0.20 | 0.80 | P |
| Q608H | RRDR | 0.75 | 0.25 | N | 0.00 | 1.00 | P | 0.00 | 1.00 | P | 0.02 | 0.98 | P | 0.30 | 0.70 | P | 0.20 | 0.80 | P |
| Q608I | RRDR | 0.00 | 1.00 | P | 0.00 | 1.00 | P | 0.00 | 1.00 | P | 0.02 | 0.98 | P | 0.35 | 0.65 | P | 0.00 | 1.00 | P |
| Q608K | RRDR | 0.75 | 0.25 | N | 0.00 | 1.00 | P | 0.00 | 1.00 | P | 0.02 | 0.98 | P | 0.30 | 0.70 | P | 0.20 | 0.80 | P |
| Q608L | RRDR | 0.00 | 1.00 | P | 0.00 | 1.00 | P | 0.00 | 1.00 | P | 0.02 | 0.98 | P | 0.34 | 0.66 | P | 0.00 | 1.00 | P |
| Q608M | RRDR | 0.75 | 0.25 | N | 0.00 | 1.00 | P | 0.00 | 1.00 | P | 0.02 | 0.98 | P | 0.34 | 0.66 | P | 0.20 | 0.80 | P |
| Q608N | RRDR | 0.75 | 0.25 | N | 0.00 | 1.00 | P | 0.00 | 1.00 | P | 0.01 | 0.99 | P | 0.27 | 0.73 | P | 0.20 | 0.80 | P |
| Q608P | RRDR | 0.75 | 0.25 | N | 0.00 | 1.00 | P | 0.00 | 1.00 | P | 0.02 | 0.98 | P | 0.30 | 0.70 | P | 0.20 | 0.80 | P |
| Q608R | RRDR | 0.75 | 0.25 | N | 0.00 | 1.00 | P | 0.00 | 1.00 | P | 0.02 | 0.98 | P | 0.31 | 0.69 | P | 0.20 | 0.80 | P |
| Q608S | RRDR | 0.75 | 0.25 | N | 0.00 | 1.00 | P | 0.00 | 1.00 | P | 0.02 | 0.98 | P | 0.30 | 0.70 | P | 0.20 | 0.80 | P |
| Q608T | RRDR | 0.75 | 0.25 | N | 0.00 | 1.00 | P | 0.00 | 1.00 | P | 0.02 | 0.98 | P | 0.30 | 0.70 | P | 0.20 | 0.80 | P |
| Q608V | RRDR | 0.00 | 1.00 | P | 0.00 | 1.00 | P | 0.00 | 1.00 | P | 0.02 | 0.98 | P | 0.34 | 0.66 | P | 0.00 | 1.00 | P |
| Q608W | RRDR | 0.00 | 1.00 | P | 0.00 | 1.00 | P | 0.00 | 1.00 | P | 0.02 | 0.98 | P | 0.32 | 0.68 | P | 0.00 | 1.00 | P |
| Q608Y | RRDR | 0.75 | 0.25 | N | 0.00 | 1.00 | P | 0.00 | 1.00 | P | 0.02 | 0.98 | P | 0.35 | 0.65 | P | 0.20 | 0.80 | P |

Table S8. The parameters for the gathered 89 KatG mutants, including 40 positive and 49 negative mutants.

| Mutation | Distance | SS | Location | PremPS | PSSM | DCS | DOMH | P_L | P_FWY | P_RKDE | N_Hydro | N_Charg | SASA_pro | SASA_sol | DE | ΔE | Type |
| --- | --- | --- | --- | --- | --- | --- | --- | --- | --- | --- | --- | --- | --- | --- | --- | --- | --- |
| H97R | 10.28 | 1 | 1 | 1.65 | 0.59 | 0.83 | -0.15 | -0.04 | 0.03 | -0.13 | 0.11 | -0.04 | 0.25 | 0.19 | -4.85 | 0.05 | P |
| R104Q | 5.78 | 3 | 0 | 1.48 | 0.54 | 0.67 | 0.05 | 0.03 | 0.03 | -0.06 | 0.06 | -0.06 | -0.18 | 0.41 | -4.88 | 0.02 | P |
| G123E | 22.58 | 4 | 1 | 1.47 | 0.57 | 1.02 | 0.19 | -0.02 | 0.05 | -0.23 | 0.05 | 0.01 | 0.11 | -0.28 | -4.73 | 0.17 | P |
| G125D | 20.35 | 1 | 1 | 1.38 | 0.62 | 0.94 | 0.27 | -0.05 | 0.00 | -0.23 | 0.03 | 0.00 | 0.20 | -0.40 | -4.78 | 0.12 | P |
| Q127E | 15.52 | 3 | 1 | 1.29 | 0.42 | 0.57 | -0.07 | -0.02 | -0.01 | -0.13 | -0.01 | 0.02 | 0.39 | 0.12 | -4.78 | 0.12 | P |
| N133T | 18.16 | 3 | 1 | 0.86 | 0.62 | 0.69 | -0.53 | -0.08 | -0.01 | -0.22 | 0.05 | 0.01 | 0.26 | 0.07 | -4.83 | 0.07 | P |
| N138S | 12.28 | 3 | 1 | 1.20 | 0.58 | 0.74 | -0.35 | -0.08 | 0.05 | -0.18 | -0.06 | -0.05 | 0.47 | 0.08 | -4.84 | 0.06 | P |
| S140N | 9.98 | 4 | 1 | -0.64 | -0.44 | -0.43 | 0.52 | 0.06 | 0.03 | 0.10 | 0.06 | -0.07 | -0.32 | -0.15 | -5.13 | -0.23 | P |
| S140R | 9.98 | 4 | 1 | 0.95 | 0.64 | 0.44 | 0.00 | -0.01 | 0.00 | -0.17 | -0.04 | 0.03 | 0.32 | -0.27 | -4.88 | 0.02 | P |
| L141F | 10.21 | 3 | 1 | 1.50 | 0.63 | 0.79 | -0.55 | -0.02 | 0.01 | -0.14 | -0.08 | -0.02 | 0.68 | 0.20 | -4.71 | 0.19 | P |
| D142G | 13.91 | 3 | 1 | 0.30 | 0.51 | 0.84 | -0.49 | 0.01 | -0.06 | -0.27 | -0.04 | -0.08 | 0.25 | -0.38 | -4.82 | 0.08 | P |
| L159P | 23.84 | 1 | 1 | 2.35 | 0.67 | 0.64 | 0.92 | -0.14 | -0.15 | -0.21 | 0.04 | -0.04 | 0.51 | 0.12 | -4.82 | 0.08 | P |
| M176T | 12.01 | 4 | 1 | 1.84 | 0.53 | 0.67 | 0.47 | -0.06 | -0.13 | -0.13 | 0.01 | 0.04 | 0.30 | 0.14 | -4.79 | 0.11 | P |
| T180K | 18.73 | 1 | 1 | 1.43 | 0.56 | 0.78 | 0.18 | -0.09 | 0.00 | -0.26 | 0.11 | 0.05 | 0.23 | -0.13 | -4.74 | 0.16 | P |
| G182R | 21.49 | 1 | 1 | 1.32 | 0.63 | 0.97 | -0.10 | -0.06 | 0.00 | -0.19 | 0.13 | 0.05 | 0.12 | -0.23 | -4.83 | 0.07 | P |
| W191G | 28.88 | 1 | 0 | 1.43 | 0.66 | 0.71 | 0.16 | 0.03 | -0.01 | -0.02 | 0.02 | 0.01 | -0.38 | 0.24 | -4.75 | 0.15 | P |
| W191R | 28.88 | 1 | 0 | 1.46 | 0.66 | 0.74 | 0.16 | 0.03 | -0.01 | -0.02 | 0.02 | 0.01 | -0.37 | 0.24 | -4.76 | 0.14 | P |
| P232S | 13.56 | 4 | 0 | 0.97 | 0.51 | 0.81 | -0.01 | -0.05 | 0.06 | -0.21 | -0.04 | -0.09 | 0.06 | -0.06 | -4.87 | 0.03 | P |
| P232R | 13.56 | 4 | 0 | 0.99 | 0.52 | 0.86 | -0.02 | -0.06 | 0.04 | -0.21 | -0.04 | -0.09 | 0.05 | -0.06 | -4.90 | 0.00 | P |
| G279D | 16.45 | 1 | 1 | 1.27 | 0.30 | 0.89 | 0.27 | 0.00 | -0.01 | -0.19 | 0.00 | -0.01 | 0.36 | -0.34 | -4.88 | 0.02 | P |
| G285D | 20.12 | 1 | 0 | 1.42 | 0.57 | 0.98 | 0.22 | 0.02 | 0.05 | -0.17 | 0.09 | 0.03 | -0.13 | -0.24 | -4.82 | 0.08 | P |
| G297V | 20.86 | 4 | 0 | 1.11 | 0.57 | 0.97 | -0.40 | 0.03 | -0.05 | -0.08 | 0.04 | 0.02 | 0.00 | 0.01 | -4.89 | 0.01 | P |
| G299S | 17.34 | 1 | 1 | 1.29 | 0.60 | 0.93 | -0.15 | -0.02 | 0.03 | -0.14 | 0.05 | -0.02 | 0.32 | -0.29 | -4.77 | 0.13 | P |
| W300C | 15.36 | 1 | 1 | 1.69 | 0.54 | 0.77 | 0.02 | -0.02 | 0.02 | -0.08 | 0.03 | 0.00 | 0.06 | 0.36 | -4.78 | 0.12 | P |
| G309D | 12.01 | 3 | 1 | 1.22 | 0.63 | 0.86 | 0.29 | -0.05 | -0.01 | -0.24 | -0.04 | -0.01 | 0.19 | -0.39 | -4.86 | 0.04 | P |
| S315T | 10.43 | 1 | 0 | 0.49 | 0.48 | 0.72 | -0.15 | 0.01 | 0.01 | -0.15 | -0.02 | -0.12 | -0.06 | -0.23 | -4.79 | 0.11 | P |
| S315N | 10.43 | 1 | 0 | 0.65 | 0.47 | 0.69 | 0.11 | 0.00 | 0.02 | -0.15 | -0.02 | -0.13 | -0.11 | -0.24 | -4.97 | -0.07 | P |
| S315R | 10.43 | 1 | 0 | 0.77 | 0.56 | 0.82 | 0.02 | 0.01 | 0.03 | -0.14 | 0.00 | -0.16 | -0.13 | -0.24 | -4.98 | -0.08 | P |
| S315I | 10.43 | 1 | 0 | 0.54 | 0.57 | 0.91 | -0.58 | 0.02 | -0.04 | -0.08 | 0.01 | -0.08 | -0.02 | -0.17 | -4.91 | -0.01 | P |
| G316D | 12.45 | 1 | 1 | 1.45 | 0.63 | 0.92 | 0.27 | -0.05 | -0.01 | -0.22 | 0.02 | -0.01 | 0.30 | -0.40 | -4.92 | -0.02 | P |
| W328L | 14.50 | 1 | 1 | 0.63 | 0.43 | 0.58 | -0.48 | 0.02 | -0.04 | -0.02 | -0.03 | -0.01 | -0.09 | 0.28 | -4.87 | 0.03 | P |
| S383P | 18.99 | 4 | 1 | 1.33 | 0.65 | 0.71 | -0.07 | 0.05 | 0.03 | -0.16 | -0.11 | 0.00 | 0.50 | -0.27 | -4.82 | 0.08 | P |
| D387H | 23.97 | 1 | 1 | 0.36 | 0.58 | 0.82 | -0.45 | -0.02 | -0.07 | -0.23 | 0.03 | -0.07 | 0.16 | -0.38 | -4.79 | 0.11 | P |
| D419H | 23.38 | 3 | 1 | 0.39 | 0.47 | 0.83 | -0.48 | -0.04 | -0.05 | -0.27 | 0.05 | 0.03 | 0.19 | -0.34 | -4.80 | 0.10 | P |
| M420T | 25.35 | 3 | 1 | 2.25 | 0.65 | 0.70 | 0.42 | -0.08 | -0.15 | -0.10 | 0.06 | 0.06 | 0.59 | 0.11 | -4.80 | 0.10 | P |
| S481L | 41.27 | 4 | 1 | 1.01 | 0.67 | 0.96 | -0.75 | 0.02 | -0.02 | -0.15 | 0.19 | 0.02 | 0.27 | -0.20 | -4.94 | -0.04 | P |
| R489S | 30.98 | 1 | 1 | 1.60 | 0.58 | 0.69 | -0.19 | -0.07 | -0.01 | -0.10 | 0.01 | -0.02 | 0.32 | 0.38 | -4.77 | 0.13 | P |
| V633A | 58.18 | 3 | 1 | 1.21 | -0.71 | 1.07 | 0.54 | 0.05 | -0.04 | -0.12 | 0.09 | 0.03 | 0.22 | 0.09 | -4.94 | -0.04 | P |
| S700P | 52.67 | 4 | 0 | 1.02 | 0.57 | 0.84 | 0.01 | -0.01 | 0.02 | -0.01 | 0.12 | 0.04 | -0.30 | -0.24 | -4.79 | 0.11 | P |
| L704S | 49.00 | 3 | 1 | 2.27 | 0.67 | 0.68 | 0.93 | -0.14 | -0.15 | -0.19 | -0.04 | -0.04 | 0.43 | 0.11 | -4.81 | 0.09 | P |
| Q36P | 40.25 | 3 | 0 | 0.94 | 0.60 | 0.61 | -0.27 | 0.01 | 0.04 | -0.08 | 0.01 | 0.03 | -0.09 | 0.07 | -4.88 | 0.02 | N |
| V47I | 36.28 | 3 | 1 | -0.89 | -0.56 | 0.02 | -0.12 | -0.11 | -0.03 | -0.08 | 0.03 | -0.01 | -0.10 | 0.08 | -4.87 | 0.03 | N |
| V68G | 28.61 | 3 | 1 | 2.62 | 0.91 | 0.67 | 0.93 | -0.08 | -0.19 | -0.06 | 0.00 | -0.05 | 0.40 | 0.09 | -4.84 | 0.06 | N |
| T77R | 20.39 | 3 | 0 | -0.31 | 0.00 | -0.64 | 0.29 | 0.01 | 0.05 | 0.14 | 0.01 | 0.01 | -0.12 | -0.05 | -4.86 | 0.04 | N |
| Q88E | 16.78 | 1 | 0 | 0.83 | 0.40 | 0.65 | 0.00 | 0.04 | 0.04 | -0.10 | -0.05 | 0.00 | -0.18 | 0.03 | -4.86 | 0.04 | N |
| G121S | 19.64 | 1 | 1 | 1.09 | 0.60 | 0.91 | -0.13 | -0.04 | 0.03 | -0.19 | 0.07 | -0.02 | 0.12 | -0.27 | -4.8 | 0.10 | N |
| G123R | 22.58 | 4 | 1 | 1.36 | 0.40 | 1.28 | -0.06 | -0.03 | 0.02 | -0.19 | 0.07 | 0.00 | 0.06 | -0.20 | -4.87 | 0.03 | N |
| G124A | 22.77 | 4 | 1 | -0.71 | 0.05 | -0.48 | 0.09 | 0.07 | 0.06 | 0.17 | -0.03 | -0.02 | -0.30 | -0.31 | -4.78 | 0.12 | N |
| G124D | 22.77 | 4 | 1 | 0.60 | 0.36 | 0.05 | 0.42 | 0.02 | 0.05 | -0.10 | 0.02 | 0.01 | 0.09 | -0.33 | -4.8 | 0.10 | N |
| D194N | 26.62 | 1 | 0 | 0.51 | 0.46 | 0.58 | -0.17 | 0.00 | 0.01 | -0.21 | 0.10 | -0.06 | 0.07 | -0.28 | -4.79 | 0.11 | N |
| L205R | 20.12 | 4 | 0 | 1.30 | 0.56 | 0.70 | 0.80 | 0.02 | -0.05 | -0.04 | 0.02 | 0.07 | -0.86 | 0.08 | -4.81 | 0.09 | N |
| D215E | 31.45 | 2 | 0 | -0.28 | -0.40 | 0.05 | 0.02 | -0.07 | -0.01 | -0.03 | 0.03 | 0.01 | 0.10 | 0.02 | -4.72 | 0.18 | N |
| G237A | 21.09 | 3 | 0 | 0.80 | 0.39 | 1.05 | -0.13 | -0.03 | 0.02 | -0.20 | -0.07 | -0.13 | 0.11 | -0.22 | -4.81 | 0.09 | N |
| M257I | 20.58 | 1 | 1 | 1.54 | 0.56 | 0.73 | -0.23 | -0.08 | 0.00 | -0.13 | 0.15 | -0.05 | 0.36 | 0.24 | -4.89 | 0.01 | N |
| V260I | 22.23 | 3 | 0 | 0.33 | 0.58 | 0.24 | -0.25 | -0.05 | -0.03 | -0.23 | -0.02 | -0.01 | 0.11 | -0.01 | -4.87 | 0.03 | N |
| G285V | 20.11 | 1 | 0 | 1.12 | 0.57 | 1.04 | -0.43 | 0.04 | -0.03 | -0.13 | 0.08 | 0.07 | -0.06 | -0.02 | -4.75 | 0.15 | N |
| Q295A | 22.59 | 3 | 1 | 0.61 | -0.37 | 1.08 | -0.24 | 0.04 | 0.04 | -0.13 | -0.06 | 0.03 | 0.09 | 0.13 | -4.93 | -0.03 | N |
| T308A | 14.40 | 1 | 1 | 0.33 | 0.61 | -0.13 | 0.02 | 0.01 | 0.02 | -0.14 | -0.02 | 0.03 | -0.02 | -0.05 | -4.89 | 0.01 | N |
| N323S | 14.28 | 1 | 0 | -0.27 | 0.02 | 0.03 | -0.24 | -0.10 | 0.00 | -0.08 | 0.01 | 0.02 | 0.00 | 0.08 | -4.78 | 0.12 | N |
| E340D | 23.90 | 1 | 0 | 0.25 | -0.55 | 0.85 | 0.05 | 0.00 | 0.00 | -0.06 | 0.07 | 0.00 | -0.24 | 0.13 | -4.87 | 0.03 | N |
| T394A | 20.68 | 3 | 1 | 0.37 | -0.38 | 0.56 | 0.03 | 0.06 | 0.01 | -0.07 | 0.04 | 0.01 | 0.23 | -0.11 | -4.84 | 0.06 | N |
| L398R | 22.55 | 3 | 0 | 1.17 | -0.18 | 0.63 | 0.79 | -0.03 | -0.01 | -0.07 | 0.00 | -0.07 | -0.08 | 0.19 | -4.88 | 0.02 | N |
| D406G | 22.59 | 3 | 0 | 0.55 | 0.58 | 0.71 | -0.30 | -0.02 | -0.02 | -0.03 | -0.01 | 0.01 | -0.18 | -0.19 | -4.84 | 0.06 | N |
| P432T | 27.38 | 1 | 1 | 1.07 | 0.52 | 0.85 | -0.13 | -0.08 | 0.02 | -0.19 | 0.09 | -0.05 | 0.10 | -0.06 | -4.8 | 0.10 | N |
| K433T | 32.84 | 1 | 0 | -0.07 | 0.30 | 0.32 | -0.27 | -0.05 | 0.01 | -0.07 | 0.07 | -0.03 | -0.44 | 0.11 | -4.82 | 0.08 | N |
| V445I | 44.66 | 1 | 0 | 0.03 | 0.32 | 0.27 | -0.26 | -0.03 | 0.02 | -0.12 | 0.04 | 0.00 | -0.21 | 0.01 | -4.85 | 0.05 | N |
| S446N | 47.54 | 1 | 0 | -0.11 | -0.32 | -0.21 | 0.33 | -0.05 | 0.00 | 0.04 | 0.01 | 0.00 | 0.28 | -0.19 | -4.82 | 0.08 | N |
| V469L | 58.62 | 3 | 1 | 0.61 | 0.32 | 0.42 | -0.27 | -0.07 | 0.00 | -0.23 | -0.03 | 0.02 | 0.39 | 0.04 | -4.85 | 0.05 | N |
| V473L | 52.79 | 3 | 1 | 1.14 | 0.57 | 0.52 | -0.35 | -0.04 | 0.00 | -0.16 | -0.04 | 0.01 | 0.59 | 0.04 | -4.91 | -0.01 | N |
| T475I | 49.99 | 3 | 1 | 0.84 | 0.67 | 0.86 | -0.64 | 0.00 | 0.01 | -0.19 | 0.01 | 0.04 | 0.11 | -0.05 | -4.89 | 0.01 | N |
| V507I | 45.56 | 4 | 1 | 0.25 | 0.34 | 0.26 | -0.27 | -0.01 | -0.04 | -0.26 | 0.02 | -0.04 | 0.22 | 0.05 | -4.75 | 0.15 | N |
| P510A | 51.07 | 4 | 0 | 0.92 | 0.55 | 0.89 | -0.10 | -0.06 | 0.02 | -0.20 | -0.04 | -0.07 | 0.01 | -0.07 | -4.83 | 0.07 | N |
| D511N | 54.67 | 4 | 0 | -0.14 | 0.32 | 0.39 | -0.23 | -0.04 | 0.00 | -0.11 | 0.00 | -0.02 | -0.34 | -0.11 | -4.77 | 0.13 | N |
| R519H | 57.42 | 3 | 0 | 0.44 | 0.45 | 0.30 | 0.04 | 0.02 | 0.00 | -0.01 | -0.02 | 0.06 | -0.62 | 0.22 | -4.73 | 0.17 | N |
| E522K | 53.83 | 3 | 0 | 0.36 | -0.06 | 0.89 | -0.22 | -0.08 | 0.04 | -0.15 | -0.03 | -0.05 | -0.09 | 0.11 | -4.87 | 0.03 | N |
| E523D | 59.21 | 3 | 0 | 0.08 | 0.33 | -0.01 | 0.00 | -0.03 | 0.02 | -0.02 | -0.01 | -0.02 | -0.26 | 0.07 | -4.8 | 0.10 | N |
| Q525K | 54.19 | 3 | 1 | 0.89 | 0.00 | 0.90 | -0.14 | -0.01 | 0.04 | -0.14 | -0.05 | -0.01 | 0.18 | 0.13 | -4.8 | 0.10 | N |
| Q525S | 54.19 | 3 | 1 | 1.01 | 0.58 | 0.76 | -0.25 | -0.07 | 0.03 | -0.13 | -0.06 | -0.04 | 0.11 | 0.08 | -4.84 | 0.06 | N |
| S527L | 60.81 | 3 | 0 | 0.42 | 0.63 | 0.73 | -0.65 | 0.01 | -0.04 | -0.10 | -0.01 | 0.00 | 0.04 | -0.19 | -4.75 | 0.15 | N |
| G534S | 58.82 | 1 | 0 | 0.67 | -0.07 | 1.08 | 0.00 | 0.01 | 0.04 | -0.09 | -0.04 | -0.01 | -0.04 | -0.22 | -4.88 | 0.02 | N |
| K537E | 54.02 | 1 | 0 | 0.98 | 0.27 | 0.59 | 0.12 | 0.03 | 0.04 | -0.07 | 0.03 | -0.03 | -0.15 | 0.14 | -4.82 | 0.08 | N |
| K557N | 65.90 | 3 | 0 | 0.34 | 0.53 | 0.65 | -0.08 | -0.02 | -0.03 | 0.07 | 0.01 | -0.14 | -0.73 | 0.09 | -4.83 | 0.07 | N |
| N562H | 64.81 | 1 | 0 | -0.14 | 0.29 | 0.34 | -0.21 | -0.06 | 0.01 | -0.13 | 0.00 | -0.01 | -0.44 | 0.06 | -4.8 | 0.10 | N |
| L598R | 45.79 | 2 | 0 | 0.68 | 0.07 | 0.74 | 0.72 | 0.01 | -0.01 | -0.06 | 0.00 | 0.01 | -0.91 | 0.11 | -4.78 | 0.12 | N |
| M609T | 39.75 | 3 | 1 | 1.60 | 0.56 | 0.61 | 0.45 | -0.06 | -0.13 | -0.04 | 0.02 | 0.00 | 0.04 | 0.15 | -4.89 | 0.01 | N |
| T625A | 49.23 | 3 | 1 | 1.61 | 0.58 | 0.73 | -0.13 | 0.00 | 0.01 | -0.09 | 0.17 | -0.12 | 0.59 | -0.14 | -4.73 | 0.17 | N |
| T667I | 64.93 | 2 | 0 | 0.31 | 0.56 | 0.45 | -0.54 | -0.03 | -0.03 | -0.09 | 0.06 | 0.06 | -0.13 | -0.01 | -4.86 | 0.04 | N |
| L696Q | 54.13 | 3 | 1 | 1.97 | 0.67 | 0.71 | 0.97 | -0.10 | -0.15 | -0.07 | -0.07 | -0.01 | -0.09 | 0.12 | -4.81 | 0.09 | N |
| V739M | 41.25 | 3 | 0 | -0.14 | 0.39 | -0.49 | -0.09 | -0.04 | 0.03 | 0.01 | 0.01 | -0.01 | 0.00 | 0.05 | -4.78 | 0.12 | N |

Table S9. The predictive results for the classifiers on the mutation type of KatG protein. P: positive, N: negative. P_N_ and P_P_ denote the probability values of being negative or positive mutation, respectively. NB: Naïve Bayes; DT: decision tree; kNN: k nearest neighbors; PNN: probabilistic neural network; SVM: support vector machine; MC: majority consensus

| Mutation | Real type | DT | | | kNN | | | NB | | | PNN | | | SVM | | | MC | | |
| --- | --- | --- | --- | --- | --- | --- | --- | --- | --- | --- | --- | --- | --- | --- | --- | --- | --- | --- | --- |
|  |  | P_N_ | P_P_ | Prediction | P_N_ | P_P_ | Prediction | P_N_ | P_P_ | Prediction | P_N_ | P_P_ | Prediction | P_N_ | P_P_ | Prediction | P_N_ | P_P_ | Prediction |
| H97R | P | 0.00 | 1.00 | P | 0.45 | 0.55 | P | 0.17 | 0.83 | P | 0.07 | 0.93 | P | 0.14 | 0.86 | P | 0.00 | 1.00 | P |
| R104Q | P | 0.04 | 0.96 | P | 0.00 | 1.00 | P | 0.38 | 0.62 | P | 0.00 | 1.00 | P | 0.14 | 0.86 | P | 0.00 | 1.00 | P |
| G123E | P | 0.00 | 1.00 | P | 0.00 | 1.00 | P | 0.00 | 1.00 | P | 0.03 | 0.97 | P | 0.26 | 0.74 | P | 0.00 | 1.00 | P |
| G125D | P | 0.00 | 1.00 | P | 0.31 | 0.69 | P | 0.07 | 0.93 | P | 0.00 | 1.00 | P | 0.26 | 0.74 | P | 0.00 | 1.00 | P |
| Q127E | P | 0.04 | 0.96 | P | 0.43 | 0.57 | P | 0.04 | 0.96 | P | 0.13 | 0.87 | P | 0.25 | 0.75 | P | 0.00 | 1.00 | P |
| N133T | P | 0.04 | 0.96 | P | 0.41 | 0.59 | P | 0.84 | 0.16 | N | 0.31 | 0.69 | P | 0.34 | 0.66 | P | 0.20 | 0.80 | P |
| N138S | P | 0.00 | 1.00 | P | 0.00 | 1.00 | P | 0.42 | 0.58 | P | 0.00 | 1.00 | P | 0.23 | 0.77 | P | 0.00 | 1.00 | P |
| S140N | P | 1.00 | 0.00 | N | 0.36 | 0.64 | P | 0.08 | 0.92 | P | 0.04 | 0.96 | P | 0.51 | 0.49 | N | 0.40 | 0.60 | P |
| S140R | P | 0.75 | 0.25 | N | 0.35 | 0.65 | P | 0.81 | 0.19 | N | 0.09 | 0.91 | P | 0.24 | 0.76 | P | 0.40 | 0.60 | P |
| L141F | P | 0.00 | 1.00 | P | 0.00 | 1.00 | P | 0.00 | 1.00 | P | 0.02 | 0.98 | P | 0.19 | 0.81 | P | 0.00 | 1.00 | P |
| D142G | P | 0.00 | 1.00 | P | 0.00 | 1.00 | P | 0.09 | 0.91 | P | 0.01 | 0.99 | P | 0.38 | 0.62 | P | 0.00 | 1.00 | P |
| L159P | P | 0.00 | 1.00 | P | 0.29 | 0.71 | P | 0.03 | 0.97 | P | 0.27 | 0.73 | P | 0.19 | 0.81 | P | 0.00 | 1.00 | P |
| M176T | P | 0.04 | 0.96 | P | 0.00 | 1.00 | P | 0.01 | 0.99 | P | 0.00 | 1.00 | P | 0.15 | 0.85 | P | 0.00 | 1.00 | P |
| T180K | P | 0.00 | 1.00 | P | 0.13 | 0.87 | P | 0.00 | 1.00 | P | 0.01 | 0.99 | P | 0.24 | 0.76 | P | 0.00 | 1.00 | P |
| G182R | P | 0.00 | 1.00 | P | 0.36 | 0.64 | P | 0.60 | 0.40 | N | 0.07 | 0.93 | P | 0.28 | 0.72 | P | 0.20 | 0.80 | P |
| W191G | P | 0.00 | 1.00 | P | 1.00 | 0.00 | N | 0.02 | 0.98 | P | 1.00 | 0.00 | N | 0.35 | 0.65 | P | 0.40 | 0.60 | P |
| W191R | P | 0.00 | 1.00 | P | 1.00 | 0.00 | N | 0.07 | 0.93 | P | 1.00 | 0.00 | N | 0.35 | 0.65 | P | 0.40 | 0.60 | P |
| P232S | P | 0.00 | 1.00 | P | 0.00 | 1.00 | P | 0.94 | 0.06 | N | 0.02 | 0.98 | P | 0.28 | 0.72 | P | 0.20 | 0.80 | P |
| P232R | P | 0.00 | 1.00 | P | 0.00 | 1.00 | P | 0.90 | 0.10 | N | 0.02 | 0.98 | P | 0.28 | 0.72 | P | 0.20 | 0.80 | P |
| G279D | P | 0.00 | 1.00 | P | 0.00 | 1.00 | P | 0.76 | 0.24 | N | 0.00 | 1.00 | P | 0.26 | 0.74 | P | 0.20 | 0.80 | P |
| G285D | P | 0.00 | 1.00 | P | 0.30 | 0.70 | P | 0.89 | 0.11 | N | 0.00 | 1.00 | P | 0.26 | 0.74 | P | 0.20 | 0.80 | P |
| G297V | P | 0.00 | 1.00 | P | 0.29 | 0.71 | P | 0.97 | 0.03 | N | 0.03 | 0.97 | P | 0.34 | 0.66 | P | 0.20 | 0.80 | P |
| G299S | P | 0.00 | 1.00 | P | 0.00 | 1.00 | P | 0.01 | 0.99 | P | 0.01 | 0.99 | P | 0.25 | 0.75 | P | 0.00 | 1.00 | P |
| W300C | P | 0.00 | 1.00 | P | 0.00 | 1.00 | P | 0.02 | 0.98 | P | 0.00 | 1.00 | P | 0.17 | 0.83 | P | 0.00 | 1.00 | P |
| G309D | P | 0.00 | 1.00 | P | 0.00 | 1.00 | P | 0.67 | 0.33 | N | 0.00 | 1.00 | P | 0.21 | 0.79 | P | 0.20 | 0.80 | P |
| S315T | P | 0.04 | 0.96 | P | 0.00 | 1.00 | P | 0.07 | 0.93 | P | 0.01 | 0.99 | P | 0.31 | 0.69 | P | 0.00 | 1.00 | P |
| S315N | P | 0.04 | 0.96 | P | 0.32 | 0.68 | P | 0.00 | 1.00 | P | 0.01 | 0.99 | P | 0.29 | 0.71 | P | 0.00 | 1.00 | P |
| S315R | P | 0.00 | 1.00 | P | 0.00 | 1.00 | P | 0.00 | 1.00 | P | 0.01 | 0.99 | P | 0.26 | 0.74 | P | 0.00 | 1.00 | P |
| S315I | P | 0.00 | 1.00 | P | 0.00 | 1.00 | P | 0.46 | 0.54 | P | 0.01 | 0.99 | P | 0.30 | 0.70 | P | 0.00 | 1.00 | P |
| G316D | P | 0.00 | 1.00 | P | 0.00 | 1.00 | P | 0.20 | 0.80 | P | 0.00 | 1.00 | P | 0.19 | 0.81 | P | 0.00 | 1.00 | P |
| W328L | P | 0.04 | 0.96 | P | 0.00 | 1.00 | P | 0.87 | 0.13 | N | 0.00 | 1.00 | P | 0.31 | 0.69 | P | 0.20 | 0.80 | P |
| S383P | P | 0.75 | 0.25 | N | 0.47 | 0.53 | P | 0.23 | 0.77 | P | 0.37 | 0.63 | P | 0.29 | 0.71 | P | 0.20 | 0.80 | P |
| D387H | P | 0.92 | 0.08 | N | 0.67 | 0.33 | N | 0.14 | 0.86 | P | 0.74 | 0.26 | N | 0.49 | 0.51 | P | 0.60 | 0.40 | N |
| D419H | P | 0.92 | 0.08 | N | 0.28 | 0.72 | P | 0.41 | 0.59 | P | 0.39 | 0.61 | P | 0.49 | 0.51 | P | 0.2 | 0.8 | P |
| M420T | P | 0.00 | 1.00 | P | 0.33 | 0.67 | P | 0.01 | 0.99 | P | 0.69 | 0.31 | N | 0.22 | 0.78 | P | 0.2 | 0.8 | P |
| S481L | P | 0.00 | 1.00 | P | 0.33 | 0.67 | P | 0.07 | 0.93 | P | 0.78 | 0.22 | N | 0.61 | 0.39 | N | 0.4 | 0.6 | P |
| R489S | P | 0.00 | 1.00 | P | 1.00 | 0.00 | N | 0.03 | 0.97 | P | 0.96 | 0.04 | N | 0.37 | 0.63 | P | 0.4 | 0.6 | P |
| V633A | P | 0.00 | 1.00 | P | 0.44 | 0.56 | P | 1.00 | 0.00 | N | 0.54 | 0.46 | N | 0.80 | 0.20 | N | 0.6 | 0.4 | N |
| S700P | P | 0.00 | 1.00 | P | 0.28 | 0.72 | P | 0.96 | 0.04 | N | 0.08 | 0.92 | P | 0.74 | 0.26 | N | 0.4 | 0.6 | P |
| L704S | P | 1.00 | 0.00 | N | 0.69 | 0.31 | N | 0.19 | 0.81 | P | 0.46 | 0.54 | P | 0.50 | 0.50 | N | 0.6 | 0.4 | N |
| Q36P | N | 0.33 | 0.67 | P | 0.29 | 0.71 | P | 1.00 | 0.00 | N | 0.59 | 0.41 | N | 0.63 | 0.37 | N | 0.6 | 0.4 | N |
| V47I | N | 0.67 | 0.33 | N | 1.00 | 0.00 | N | 1.00 | 0.00 | N | 0.96 | 0.04 | N | 0.85 | 0.15 | N | 1 | 0 | N |
| V68G | N | 1.00 | 0.00 | N | 0.69 | 0.31 | N | 0.02 | 0.98 | P | 0.98 | 0.02 | N | 0.19 | 0.81 | P | 0.6 | 0.4 | N |
| T77R | N | 0.04 | 0.96 | P | 0.33 | 0.67 | P | 1.00 | 0.00 | N | 0.03 | 0.97 | P | 0.64 | 0.36 | N | 0.4 | 0.6 | P |
| Q88E | N | 0.75 | 0.25 | N | 0.00 | 1.00 | P | 0.99 | 0.01 | N | 0.00 | 1.00 | P | 0.34 | 0.66 | P | 0.4 | 0.6 | P |
| G121S | N | 0.00 | 1.00 | P | 0.22 | 0.78 | P | 0.41 | 0.59 | P | 0.01 | 0.99 | P | 0.29 | 0.71 | P | 0 | 1 | P |
| G123R | N | 0.00 | 1.00 | P | 0.00 | 1.00 | P | 0.97 | 0.03 | N | 0.04 | 0.96 | P | 0.28 | 0.72 | P | 0.2 | 0.8 | P |
| G124A | N | 0.92 | 0.08 | N | 0.35 | 0.65 | P | 1.00 | 0.00 | N | 0.29 | 0.71 | P | 0.67 | 0.33 | N | 0.6 | 0.4 | N |
| G124D | N | 0.92 | 0.08 | N | 0.00 | 1.00 | P | 0.96 | 0.04 | N | 0.11 | 0.89 | P | 0.45 | 0.55 | P | 0.4 | 0.6 | P |
| D194N | N | 0.92 | 0.08 | N | 1.00 | 0.00 | N | 0.76 | 0.24 | N | 1.00 | 0.00 | N | 0.54 | 0.46 | N | 1 | 0 | N |
| L205R | N | 0.04 | 0.96 | P | 0.34 | 0.66 | P | 0.27 | 0.73 | P | 0.07 | 0.93 | P | 0.24 | 0.76 | P | 0 | 1 | P |
| D215E | N | 0.92 | 0.08 | N | 0.56 | 0.44 | N | 0.65 | 0.35 | N | 0.62 | 0.38 | N | 0.77 | 0.23 | N | 1 | 0 | N |
| G237A | N | 0.00 | 1.00 | P | 0.31 | 0.69 | P | 0.33 | 0.67 | P | 0.02 | 0.98 | P | 0.41 | 0.59 | P | 0 | 1 | P |
| M257I | N | 0.00 | 1.00 | P | 0.33 | 0.67 | P | 0.22 | 0.78 | P | 0.00 | 1.00 | P | 0.26 | 0.74 | P | 0 | 1 | P |
| V260I | N | 0.04 | 0.96 | P | 0.29 | 0.71 | P | 1.00 | 0.00 | N | 0.13 | 0.87 | P | 0.54 | 0.46 | N | 0.4 | 0.6 | P |
| G285V | N | 0.00 | 1.00 | P | 0.30 | 0.70 | P | 0.00 | 1.00 | P | 0.00 | 1.00 | P | 0.32 | 0.68 | P | 0 | 1 | P |
| Q295A | N | 1.00 | 0.00 | N | 0.28 | 0.72 | P | 0.98 | 0.02 | N | 0.16 | 0.84 | P | 0.45 | 0.55 | P | 0.4 | 0.6 | P |
| T308A | N | 0.04 | 0.96 | P | 0.00 | 1.00 | P | 1.00 | 0.00 | N | 0.00 | 1.00 | P | 0.38 | 0.62 | P | 0.2 | 0.8 | P |
| N323S | N | 0.04 | 0.96 | P | 0.00 | 1.00 | P | 0.99 | 0.01 | N | 0.01 | 0.99 | P | 0.55 | 0.45 | N | 0.4 | 0.6 | P |
| E340D | N | 0.67 | 0.33 | N | 0.66 | 0.34 | N | 1.00 | 0.00 | N | 0.79 | 0.21 | N | 0.56 | 0.44 | N | 1 | 0 | N |
| T394A | N | 1.00 | 0.00 | N | 0.31 | 0.69 | P | 1.00 | 0.00 | N | 0.06 | 0.94 | P | 0.49 | 0.51 | P | 0.4 | 0.6 | P |
| L398R | N | 1.00 | 0.00 | N | 0.31 | 0.69 | P | 0.98 | 0.02 | N | 0.11 | 0.89 | P | 0.37 | 0.63 | P | 0.4 | 0.6 | P |
| D406G | N | 0.04 | 0.96 | P | 0.28 | 0.72 | P | 0.99 | 0.01 | N | 0.18 | 0.82 | P | 0.45 | 0.55 | P | 0.2 | 0.8 | P |
| P432T | N | 0.92 | 0.08 | N | 1.00 | 0.00 | N | 0.63 | 0.37 | N | 1.00 | 0.00 | N | 0.40 | 0.60 | P | 0.8 | 0.2 | N |
| K433T | N | 0.00 | 1.00 | P | 0.66 | 0.34 | N | 1.00 | 0.00 | N | 0.71 | 0.29 | N | 0.70 | 0.30 | N | 0.8 | 0.2 | N |
| V445I | N | 0.92 | 0.08 | N | 1.00 | 0.00 | N | 1.00 | 0.00 | N | 0.96 | 0.04 | N | 0.82 | 0.18 | N | 1 | 0 | N |
| S446N | N | 0.00 | 1.00 | P | 0.44 | 0.56 | P | 1.00 | 0.00 | N | 0.57 | 0.43 | N | 0.89 | 0.11 | N | 0.6 | 0.4 | N |
| V469L | N | 1.00 | 0.00 | N | 0.51 | 0.49 | N | 1.00 | 0.00 | N | 0.61 | 0.39 | N | 0.87 | 0.13 | N | 1 | 0 | N |
| V473L | N | 1.00 | 0.00 | N | 0.27 | 0.73 | P | 0.97 | 0.03 | N | 0.08 | 0.92 | P | 0.77 | 0.23 | N | 0.6 | 0.4 | N |
| T475I | N | 0.00 | 1.00 | P | 0.69 | 0.31 | N | 0.99 | 0.01 | N | 0.60 | 0.40 | N | 0.74 | 0.26 | N | 0.8 | 0.2 | N |
| V507I | N | 0.92 | 0.08 | N | 0.29 | 0.71 | P | 0.22 | 0.78 | P | 0.39 | 0.61 | P | 0.80 | 0.20 | N | 0.4 | 0.6 | P |
| P510A | N | 0.67 | 0.33 | N | 0.67 | 0.33 | N | 1.00 | 0.00 | N | 0.25 | 0.75 | P | 0.77 | 0.23 | N | 0.8 | 0.2 | N |
| D511N | N | 0.67 | 0.33 | N | 0.16 | 0.84 | P | 1.00 | 0.00 | N | 0.17 | 0.83 | P | 0.90 | 0.10 | N | 0.6 | 0.4 | N |
| R519H | N | 0.00 | 1.00 | P | 0.37 | 0.63 | P | 0.08 | 0.92 | P | 0.45 | 0.55 | P | 0.85 | 0.15 | N | 0.2 | 0.8 | P |
| E522K | N | 0.67 | 0.33 | N | 0.18 | 0.82 | P | 1.00 | 0.00 | N | 0.10 | 0.90 | P | 0.86 | 0.14 | N | 0.6 | 0.4 | N |
| E523D | N | 0.67 | 0.33 | N | 0.64 | 0.36 | N | 1.00 | 0.00 | N | 0.70 | 0.30 | N | 0.91 | 0.09 | N | 1 | 0 | N |
| Q525K | N | 0.67 | 0.33 | N | 0.18 | 0.82 | P | 0.99 | 0.01 | N | 0.12 | 0.88 | P | 0.79 | 0.21 | N | 0.6 | 0.4 | N |
| Q525S | N | 0.67 | 0.33 | N | 0.17 | 0.83 | P | 1.00 | 0.00 | N | 0.11 | 0.89 | P | 0.76 | 0.24 | N | 0.6 | 0.4 | N |
| S527L | N | 0.00 | 1.00 | P | 0.64 | 0.36 | N | 0.35 | 0.65 | P | 0.83 | 0.17 | N | 0.89 | 0.11 | N | 0.6 | 0.4 | N |
| G534S | N | 0.67 | 0.33 | N | 0.43 | 0.57 | P | 1.00 | 0.00 | N | 0.57 | 0.43 | N | 0.86 | 0.14 | N | 0.8 | 0.2 | N |
| K537E | N | 0.67 | 0.33 | N | 0.24 | 0.76 | P | 1.00 | 0.00 | N | 0.09 | 0.91 | P | 0.79 | 0.21 | N | 0.6 | 0.4 | N |
| K557N | N | 0.00 | 1.00 | P | 0.52 | 0.48 | N | 0.86 | 0.14 | N | 0.99 | 0.01 | N | 0.90 | 0.10 | N | 0.8 | 0.2 | N |
| N562H | N | 0.00 | 1.00 | P | 0.51 | 0.49 | N | 1.00 | 0.00 | N | 0.97 | 0.03 | N | 0.94 | 0.06 | N | 0.8 | 0.2 | N |
| L598R | N | 0.00 | 1.00 | P | 0.34 | 0.66 | P | 0.89 | 0.11 | N | 0.69 | 0.31 | N | 0.69 | 0.31 | N | 0.6 | 0.4 | N |
| M609T | N | 0.92 | 0.08 | N | 0.28 | 0.72 | P | 0.95 | 0.05 | N | 0.60 | 0.40 | N | 0.47 | 0.53 | P | 0.6 | 0.4 | N |
| T625A | N | 0.00 | 1.00 | P | 1.00 | 0.00 | N | 0.00 | 1.00 | P | 0.62 | 0.38 | N | 0.63 | 0.37 | N | 0.6 | 0.4 | N |
| T667I | N | 0.00 | 1.00 | P | 0.53 | 0.47 | N | 1.00 | 0.00 | N | 0.98 | 0.02 | N | 0.92 | 0.08 | N | 0.8 | 0.2 | N |
| L696Q | N | 0.67 | 0.33 | N | 0.22 | 0.78 | P | 0.48 | 0.52 | P | 0.11 | 0.89 | P | 0.58 | 0.42 | N | 0.4 | 0.6 | P |
| V739M | N | 0.92 | 0.08 | N | 0.71 | 0.29 | N | 1.00 | 0.00 | N | 0.87 | 0.13 | N | 0.83 | 0.17 | N | 1 | 0 | N |
